# Cardiomyocyte Microvesicles Contain DNA/RNA and Convey Biological Messages to Target Cells

**DOI:** 10.1371/journal.pone.0034653

**Published:** 2012-04-10

**Authors:** Anders Waldenström, Nina Gennebäck, Urban Hellman, Gunnar Ronquist

**Affiliations:** 1 Department of Public Health and Clinical Medicine/Medicine, Umeå University, Umeå, Sweden; 2 Heart Center, Umeå University Hospital, Umeå, Sweden; 3 Department of Medical Sciences, Clinical Chemistry, University Hospital of Uppsala, Uppsala, Sweden; Northwestern University, United States of America

## Abstract

**Background:**

Shedding microvesicles are membrane released vesicles derived directly from the plasma membrane. Exosomes are released membrane vesicles of late endosomal origin that share structural and biochemical characteristics with prostasomes. Microvesicles/exosomes can mediate messages between cells and affect various cell-related processes in their target cells. We describe newly detected microvesicles/exosomes from cardiomyocytes and depict some of their biological functions.

**Methodology/Principal Findings:**

Microvesicles/exosomes from media of cultured cardiomyocytes derived from adult mouse heart were isolated by differential centrifugation including preparative ultracentrifugation and identified by transmission electron microscopy and flow cytometry. They were surrounded by a bilayered membrane and flow cytometry revealed presence of both caveolin-3 and flotillin-1 while clathrin and annexin-2 were not detected. Microvesicle/exosome mRNA was identified and out of 1520 detected mRNA, 423 could be directly connected in a biological network. Furthermore, by a specific technique involving TDT polymerase, 343 different chromosomal DNA sequences were identified in the microvesicles/exosomes. Microvesicle/exosomal DNA transfer was possible into target fibroblasts, where exosomes stained for DNA were seen in the fibroblast cytosol and even in the nuclei. The gene expression was affected in fibroblasts transfected by microvesicles/exosomes and among 333 gene expression changes there were 175 upregulations and 158 downregulations compared with controls.

**Conclusions/Significance:**

Our study suggests that microvesicles/exosomes released from cardiomyocytes, where we propose that exosomes derived from cardiomyocytes could be denoted “cardiosomes”, can be involved in a metabolic course of events in target cells by facilitating an array of metabolism-related processes including gene expression changes.

## Introduction

Many cells have the capacity to release microvesicles of endocytic origin to the extracellular space. The first cellular system to be explored in this regard was the prostate acinar cells. We showed more than 30 years ago that human prostatic fluid and therewith seminal plasma contains membrane surrounded, nano-sized (40–490 nm), secretory granules and microvesicles [1], [2], [3]. Subsequent studies revealed that these extracellularly occurring organelles, now denoted prostasomes, had their intracellular correspondence to similar organelles inside another larger organelle, a so called storage vesicle [4]. These storage vesicles should be understood as being equivalent to multivesicular bodies (MVB) of late endosomal origin [4]. Accordingly, the release of prostasomes was the result of a fusion between the membrane surrounding the storage vesicle and the plasma membrane of the secretory cell of the prostate gland (exocytosis) [4], [5]. The prostasomal membrane displayed extraordinary properties with unusually high content of the phospholipid sphingomyelin and a high cholesterol/phospholipid ratio rendering the membrane highly ordered and stable [6]. Also, the protein composition is a mosaic of various molecules [7], [8], [9].

Exosomes constitute the correspondence to prostasomes in other cell types. Exosome is nowadays used as a family name where prostasome exemplifies a specific name denoting the cell type of origin. Exosomes were originally thought to provide unconventional means for removal of redundant membrane proteins from reticulocytes [10], [11]. After that they were demonstrated to be secretory products in the culture medium of various mammalian cell types including those of B-cell origin [12], dendritic cells [13], mast cells [14], T-cells [15], platelets [16], intestinal epithelial cells [17], Schwann cells [18], neuronal cells [19], tumour cells and tumour cell lines [20], [21], [22].

Since prostasomes can interact with spermatozoa [23], [24], [25] it was early suspected that they could exert intercellular messenger function in favour of the fertilization process [26], [27], [28]. Similarly, it has become clear that exosomes have signalling function [29], [30]. This is in line with the establishment of DNA presence in prostasomes [31] and microvesicles/exosomes [32] and RNA presence in other exosomes [33], [34], [35].

We now demonstrate that the cardiomyocyte, generally not considered secretory, releases microvesicles/exosomes with capacity to transfer genetic information to target cells.

## Materials and Methods

### Cell culture

HL-1, a cardiomyocyte cell line derived from adult mouse heart [36], was obtained from Dr. W.C. Claycomb (Louisiana State University Medical Center, New Orleans). HL-1 is a line of immortalized cells of cadiomyocyte origin that has been used as a complement to native cardiomyocytes in e.g. studies of cardiac gene regulation [37]. Cardiomyocytes were cultured in Claycomb medium (JRH. Biosciences) supplemented with 10% fetal bovine serum (JRH. Biosciences), 0.1 mmol/L norepinephrine (10 mmol/L norepinephrine (Sigma-Aldrich) was diluted 100-fold in 30 mmol/L ascorbic acid (Sigma-Aldrich)), 2 mmol/L L-glutamine (Life Technologies), and 100 U/mL penicillin, 100 µg/mL streptomycin (Life Technologies).

During culture, the medium was changed routinely every 24 h.

NIH 3T3 cells [38] (ATCC CRL-1658, LGC Standards AB) were cultured in Dulbecco's modified Eagle's medium (DMEM, Fisher Scientific) containing 10% calf serum (JRH, Biosciences), 2 mmol/L L-glutamine, 100 U/mL penicillin, and 100 µg/mL streptomycin.

All cultures were kept in an atmosphere of 95% air-5% CO_2_, 37°C at a relative humidity of approximately 95%.

After 72 h, cells were grown to confluence and all media were replaced with serum-free and antibiotic-free media for 24 or 48 h. Media were collected and the cells were then washed with ice-cold phosphate buffered saline (PBS) (without calcium and magnesium, Fisher Scientific), and subsequently harvested by scraping and placed in RNAlater (Qiagen).

### Microvesicle/exosome isolation

Media were centrifuged to remove cell debris, 3,000×g for 20 min at 4°C, repeated three times, followed by 10,000×g for 20 min at 4°C, repeated three times. The acquired supernatants were ultracentrifuged at 130,000×g (49,000 rpm) for 2 h at 4°C in an MLS-50 rotor and a Beckman Optima™ MAX-E Ultracentrifuge (Beckman Coulter) to separate microvesicle/exosome pellet and medium supernatant containing soluble molecules. Pellet was dissolved in PBS. A similar ultracentrifugation of the fetal bovine serum alone was carried out to rule out the presence of any microvesicles/exosomes in the culture medium.

### Flow cytometry analysis

Fluorescence-activated cell sorter (FACS) was used to detect proteins on microvesicle/exosome surfaces. Isolated microvesicles/exosomes were stained with 250 ng mouse anti-annexin-2, mouse anti-clathrin heavy chain, mouse anti-flotillin-1 and mouse anti-caveolin-3 (BD Biosciences) in 100 µL PBS, for 20 min, in the dark on ice. After adding 1.9 mL PBS and an additional ultracentrifugation to wash the pellet, it was resuspended in 100 µL PBS and 1 µL rat anti-mouse IgG phycoerythrin (PE) and incubated for 20 min, in the dark on ice. The ultracentrifugation was repeated and the pellet resuspended in PBS. Microvesicles/exosomes were analyzed on FACSCalibur (Becton Dickinson).

To evaluate if DNA is present inside or outside the microvesicles/exosomes, the FACS was used for analysis on DNA stained microvesicles/exosomes. 250 µL of microvesicles/exosomes (from a 1 mL microvesicle/exosome stock solution) was incubated with acridine orange (AO) (Invitrogen) to a final concentration of 20 µmol/L. AO is membrane permeable under ordinary conditions according to the manufacturer. Another 250 µL of the microvesicle/exosome stock solution was incubated with propidium iodide (PI) (Sigma) to a final PI concentration of 30 µmol/L. As a positive control an additional 250 µL of the microvesicle/exosome stock solution was incubated with PI and genomic DNA to a final PI concentration of 30 µmol/L. PI is not membrane permeable under ordinary conditions according to the manufacturer. As control, unstained microvesicles/exosomes, (from the stock solution) treated in the same way as the stained samples, was used. Prepared staining solutions (PI (30 µmol/L) and AO (20 µmol/L), respectively) were incubated for 90 min at 21°C, protected from light. The microvesicles/exosomes in the respective staining solution were pelleted by ultracentrifugation at 100,000×g for 2 h. Supernatants were discarded and pellets were washed twice with PBS and the pellets were resolved in PBS and adjusted to the same starting volume (1 mL).

The DNA-stained microvesicle/exosome samples were prepared for flow cytometric analysis by adding 500 µL of the samples to four tubes. The fluorescence was analyzed using a FACSCalibur (Becton Dickinson) at appropriate fluorescence emitting wavelengths; 525±15 nm for AO and 670/Long pass (LP) for PI.

### Electron microscopy

For electron microscopy the microvesicles/exosomes were fixed in a solution containing 3% glutaraldehyde in 75 mmol/L sodium cacodylate buffer (pH 7.4) with 4% polyvinylpyrolidone and 2 mmol/L CaCl_2_, for 6 h. They were subsequently rinsed in the same buffer for one hour, and then post-fixed in 1% osmium tetroxide over night at 4°C. After another rinse in buffer the sample was dehydrated in a graded series of acetone and then embedded in an epoxy resin.

Ultrathin sections (70 nm) were cut, and collected on formvar coated copper grids and then contrasted with uranyl acetate and lead citrate for electron microscopy performed with a JEOL 1200-EX (Jeol Ltd.).

### Identification of microvesicular/exosomal contents

DNA was isolated with GenElute Mammalian Genomic DNA Miniprep Kit (Sigma-Aldrich) from a microvesicular pellet prepared from 18 mL Claycomb medium after 48 h incubation with cardiomyocytes. To add a poly-T tail, DNA was incubated with 25 µL 100 mmol/L dGTP (Gibco BRL, Life Technologies) and terminal deoxynucleotidyl transferase (TdT) (Invitrogen) for 30 min at 37°C, according to manufacturer's protocol. The constructed cDNA was purified and transcribed to synthesize biotinylated cRNA with Illumina Totalprep RNA Amplification Kit (Ambion). Total RNA was isolated with RNEASY Mini Kit (Qiagen) from cardiomyocytes and microvesicular/exosomal pellets prepared from 18 mL Claycomb medium after 48 h incubation with cardiomyocytes. Aliquots of RNA were converted to biotinylated double-stranded cRNA according to the specifications of the Illumina Totalprep RNA Amplification Kit (Ambion). The labeled cRNA samples were hybridized to MouseRef-8 Expression Beadchip (Illumina), incubated with streptavidin-Cy3 and scanned on the Illumina Beadstation GX. Illumina Beadstudio software, version 3.3.7. was used to normalize intensity data with the Beadstudio cubic spline algorithm. Significance detection P-value was set at <0.01. All data are MIAME compliant and are available through NCBIs Gene Expression Omnibus (GEO) and are accessible through GEO Series accession number GSE21707. To avoid detecting false positive genes due to low signal intensity, a minimum signal intensity of >50 was utilized (2.5 times highest background signal). Identified mRNAs had to be detected both in cardiomyocytes and microvesicles/exosomes to be considered as positively detected. Since cardiomyocytes reasonably are the source of microvesicular/exosomal mRNA, they should themselves contain the same mRNA.

### Microvesicular/exosomal DNA transfer into target fibroblasts

Microvesicles/exosomes were stained with 20 µmol/L AO (Invitrogen) for 90 min, in the dark at room temperature. The sample was diluted to 4 mL and ultracentrifuged at 130,000×g (49,000 rpm) for 2 h at 4°C. The supernatant was removed to eliminate contamination of unincorporated AO. The AO-stained microvesicles/exosomes suspended in 1 mL PBS were then put in a dialysis bag with a 3,500 MWCO dialysis membrane (Spectra/Por) and dialysed against 300 mL PBS for 24 h with one change of dialysis buffer after 5 h. The sample was ultracentrifuged (130,000×g for 2 h at 4°C) and the pellet dissolved in DMEM and incubated for 3 h with fibroblasts, grown for 24 h on a cell culture microscope slide (Falcon). The slide was subsequently mounted with DAPI to stain fibroblast nuclei and studied in a Nikon Eclipse E800 confocal microscope. Light microscope was used to add a layer in images to visualize cell borders.

### Microvesicular/exosomal induced effects on target cells

Fibroblasts, grown on 6-well plates, were incubated for 48 h with serum-free and antibiotic-free Claycomb medium, previously incubated for 24 h with cardiomyocytes.

A part of the same Claycomb medium was ultracentrifuged, 130,000×g for 2 h at 4°C and the supernatant was also incubated for 48 h with fibroblasts. Isolated microvesicles/exosomes from cardiomyocytes incubated for 48 h in Claycomb media were dissolved in DMEM and incubated with fibroblasts for 48 h. These stimulated fibroblasts were compared to control fibroblasts incubated in fresh Claycomb medium and DMEM, respectively. RNA was prepared from fibroblasts, labelled and hybridized to MouseRef-8 Expression Beadchip (Illumina), as described above.

To determine differentially expressed genes microarray data were analyzed using gene expression module in Beadstudio software, version 3.3.7. Intensity data were normalized using the Beadstudio cubic spline algorithm. Significant differential expression was calculated using the Illumina Beadstudio software by applying normalization using the Beadstudio cubic spline algorithm and multiple testing corrections using Benjamini and Hochberg False Discovery Rate (FDR) [39], [40]. Detection P-value was set at <0.05. All data are MIAME compliant and are available through NCBIs Gene Expression Omnibus (GEO) and are accessible through GEO Series accession number GSE21677. The gene expression fold change of the stimulated cells was calculated as the average signal value relative to the average signal value for the control cells. A significant up-regulation was defined as a foldchange ≥1.5 and a significant down-regulation was defined as foldchange ≤0.67. Statistical significance was set at P<0.05. To avoid selecting genes with high foldchange due to low signal intensity, a minimum signal intensity value was used. For up-regulated genes the signal intensity was set at >50 in the stimulated cell group. For down-regulated genes the signal intensity was set at >50 in the control cell group. Filtering of the differentially expressed genes can be found in table 1.

**Table 1:** Filtering of differentially expressed genes.

Filtering step	Fb with Claycomb medium		Fb with Claycomb medium supernatant		Fb with ultra- centrifuged pellet	
DifferentialP-value<0.05	1384		501		656	
	695↑	689↓	201↑	300↓	280↑	376↓
	↓		↓		↓	
FDR	400		102		249	
	213↑	187↓	21↑	81↓	88↑	161↓
	↓		↓		↓	
Detection	335		96		209	
P-value<0.05	177↑	158↓	21↑	75↓	75↑	134↓
	↓		↓		↓	
Foldchange>1.5, <0.67	333		96		201	
	175↑	158↓	21↑	75↓	72↑	129↓
	↓		↓		↓	
Avg. sign.	**333**		**96**		**161**	
>50	175↑	158↓	21↑	75↓	65↑	96↓

Fibroblasts incubated for 48 h with Claycomb medium, previously incubated for 24 h with cardiomyocytes were compared to fibroblasts incubated with fresh Claycomb medium.

Fibroblasts incubated for 48 h with supernatant from ultracentrifuged Claycomb medium, previously incubated for 24 h with cardiomyocytes were compared to fibroblasts incubated with fresh Claycomb medium.

Fibroblasts incubated for 48 h with pellet from ultracentrifuged Claycomb medium, previously incubated for 48 h with cardiomyocytes. Pellet was dissolved in DMEM and compared to fibroblasts incubated with fresh DMEM.

False Discovery Rate (FDR) was used for corrections for multiple testing. Significant up-regulation was defined as a foldchange >1.5 and significant down-regulation was defined as foldchange <0.67. A minimum signal intensity value of 50 was utilized. Abbreviations: Cm, cardiomyocytes; Fb, fibroblasts; sup. supernatant after ultracentrifugation; Avg. sign., average signal; FDR, False Discovery Rate; ↑, up-regulated; ↓, down-regulated; DMEM, Dulbecco's modified Eagle's medium.

## Results

### Recovery, ultrastructural and biochemical characteristics of microvesicles/exosomes

**Figure 1:**
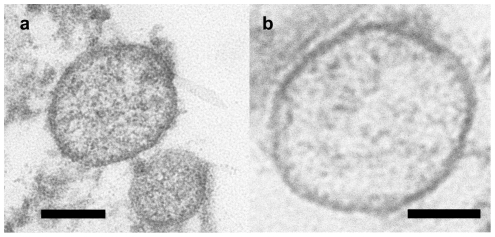
Transmission electron microscopy of purified microvesicles/exosomes. A) Microvesicles/exosomes displaying an electron dense appearance, and B) electron lucent appearance. Bar represents 100 nm.

**Figure 2:**
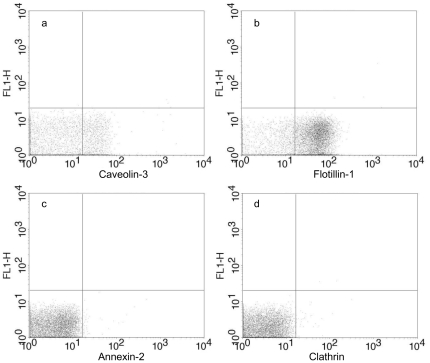
Detection of proteins on microvesicle/exosome surface with flow cytometry. Microvesicles/exosomes prepared from Claycomb culture medium was incubated with antibodies conjugated with phycoerythrin (PE). A) Mouse anti-caveolin-3, was detected on approximately 30% of the microvesicles/exosomes. B) Mouse anti-flotillin-1, was detected on approximately 80% of the microvesicles/exosomes. C) Mouse anti-annexin-2, was not detected on the microvesicles/exosomes. D) Mouse anti-clathrin heavy chain, was not detected on the microvesicles/exosomes. The distribution of exosomes presenting caveolin-3 and flotillin-1 indicates that the sample contains more than one population of microvesicles/exosomes.

We obtained microvesicles/exosomes released by murine cardiomyocytes. Microvesicles/exosomes were isolated from the culture medium by differential centrifugation and preparative ultracentrifugation. A corresponding maneuvre comprising the culture medium alone did not reveal any presence of microvesicles/exosomes. The isolated microvesicles/exosomes were subjected to transmission electron microscopy revealing small, rounded vesicles (40–300 nm) surrounded by a bilayered membrane. Some of them had a distinct electron dense appearance while others displayed an electron lucent interior (Figure 1 A–B). Furthermore, caveolin-3 and flotillin-1 were detected by flow cytometry of the microvesicles/exosomes (approximately 80% of the microvesicle/exosome population was positive for flotillin-1 and 30% for caveolin-3, indicative of a certain degree of heterogeneity), whereas annexin-2 and clathrin were not detected (Figure 2 A–D).

### Identification of microvesicle/exosome contents

**Figure 3:**
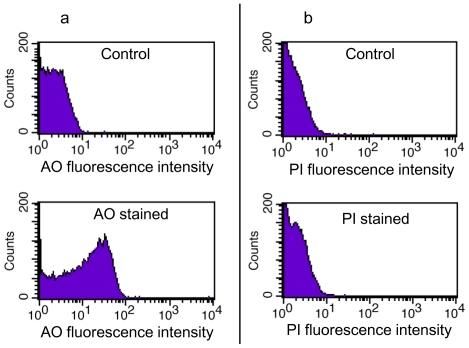
Flow cytometry of DNA-stained microvesicles/exosomes. A) Enhanced fluorescence at the 530±15 nm channel of membrane permeable acridine orange-stained microvesicles/exosomes (below) in comparison with unstained microvesicles/exosomes (above). B) Weak or no fluorescence at 670 nm/LP channel of membrane impermeable propidium iodide-stained microvesicles/exosomes (below) not differing from unstained microvesicles/exosomes (above).

Since DNA unambiguously is present in prostasomes [31], [41], we were interested in investigating presence of DNA in cardiomyocyte microvesicles/exosomes. DNA was prepared by conventional methods, see Methods. Conventional cloning however did not reveal any DNA, probably due to too little material. By use of *i.a.* TDT polymerase, poly A was added at the end of the DNA strand and a cDNA was constructed. This was labelled with biotin and converted to cRNA therewith making it adaptable to a commercial chip. Hybridisation revealed 343 different chromosomal DNA sequences when using a P-value less than 0.01 and a signal power more than 50. According to the list of genes in Table S1, all identified sequences are chromosomal. This does not rule out some viral DNA (incorporated in chromosomal DNA and/or from virus) but definitely shows that cardimyocyte DNA is enriched in microvesicles/exosomes. Data and information are available through NCBIs Gene Expression Omnibus (GEO) and are accessible through GEO Series accession number GSE21707. The AO (membrane permeable)-stained microvesicles/exosomes showed an enhanced fluorescence (Figure 3A), while PI (membrane impermeable)-stained microvesicles/exosomes without and with genomic DNA had low or no fluorescence, similar to the unstained microvesicles/exosomes (Figure 3B), suggesting that DNA is present only inside the thoroughly washed microvesicles/exosomes. The AO-stained microvesicles/exosomes also showed fluorescence for RNA, reflected by wavelength leakage into the 670/LP channel.

We extracted total RNA from the microvesicles/exosomes. Genes/proteins (for which identified mRNAs are encoding) were used in the bioinformatic data base and a biological network was drawn (Figure S1). The microvesicles/exosomes contained 1595 detected mRNA of which 1520 also were detected in cardiomyocytes. Out of these 1520 detected mRNAs in the microvesicles/exosomes, 423 could be directly connected to a biological network without addition of any extra genes/proteins (Table S2). Furthermore, a network of ribosomal genes/proteins was possible to establish. Accordingly, 35 genes coding for proteins in the small and large ribosomal subunit and 8 additional genes could be connected to a network (Figure S2). Finally 33 genes coding for proteins in mitochondria could be detected (Figure S3). Data and information are available through NCBIs Gene Expression Omnibus (GEO) and are accessible through GEO Series accession number GSE21707.

### Microvesicle/exosome DNA transfer into target fibroblasts

**Figure 4:**
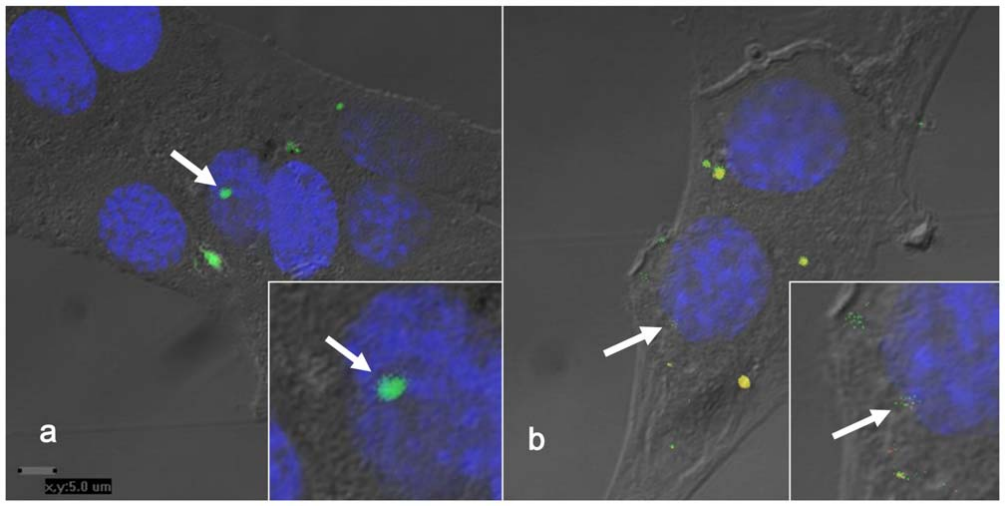
Confocal microscopy images of fibroblasts incubated with microvesicles/exosomes stained with acridine orange. Confocal microscopy picture of DNA-stained microvesicles/exosomes after dialysis, ultracentrifugation and resuspension in DMEM. After incubation with fibroblasts for 3 h at 37°C the DNA-staining localizes in fibroblasts to and inside the nuclear membrane. Additional light microscopy was used to add a layer in images to visualize cell borders. Arrows in A) and B) indicate acridine orange staining inside nuclei. B) also visualizes red wave length which detects acridine orange staining for RNA. Yellow staining shows colocalization of DNA and RNA.

Microvesicular/exosomal DNA and RNA were stained with AO. The AO-stained microvesicles/exosomes were thereafter dialysed for 24 h (with one change of dialysis buffer) and ultracentrifuged (130,000×g and 2 h at 4°C), to get rid of any trace amounts of irrelevant AO. Examination by confocal microscopy of fibroblasts incubated with AO-stained microvesicles/exosomes revealed AO-stained, intracellular DNA spots localized to and inside the nuclear membrane as well as spots of RNA (Figure 4 A–B).

### Microvesicle/exosome induced effects on target cells

When fibroblasts were transfected with microvesicles/exosomes a clearcut effect on gene expression was found. In more detail culture medium from cardiomyocytes induced 333 gene expression changes including 175 upregulations and 158 downregulations when compared to control medium. This culture medium gave rise to two compartments after ultracentrifugation: the microvesicle/exosome-deficient supernatant that induced only 96 changes (21 up, 75 down) and the microvesicle/exosome-enriched pellet resuspended in fresh DMEM that induced about 70% more changes (65 up and 96 down, Table S3). Data and information are available through NCBIs Gene Expression Omnibus (GEO) and are accessible through GEO Series accession number GSE21677.

## Discussion

Parenchymal cells are believed to mould microenvironment components and affect various functions, mainly by pathways involving cell-to-cell contact and the release of soluble factors like autocrine products. However, an alternative novel mechanism that is now emerging involves the active release, as we show here, by cardiomyocytes of nucleic acid containing microvesicles/exosomes, with the capacity of complementary actions. Gupta and Knowlton [42] demonstrated an exosomal release by cardiac cells. Apparently lipid rafts were involved in this process, since inhibition of lipid raft formation reduced the release of HSP 60, that is closely bound to the exosomal membrane [42]. Exosomes like prostatomes contain lipid rafts [6], and caveolin-1 was found to be associated with both prostasomes [21] and exosomes [42]. The present study depicts a new concept in the view of cardiomyocyte communication with other cells, proposing that microvesicles/exosomes generated by the cardiomyocytes are able to transfer a diverse array of genetic information (DNA and RNA) to other cells here exemplified by fibroblasts as target cells. We found mRNA intended for both small and large ribosomal subunits as well as mRNA coding for proteins involved in mitochondrial energy generation from cardiomyocyte-derived microvesicles/exosomes. This implies that the genetic information harboured in microvesicles/exosomes might participate in some protein production in transfected cells, including means of energy generation for that production at distant sites.

Apparently, DNA and RNA can be incorporated into microvesicles/exosomes and transferred to target cells here represented by fibroblasts. However, it remains elusive how the nucleic acids are sorted into microvesicles/exosomes. The microvesicle/exosome-linked nucleic acid may play a role in a variety of cell physiologic phenomena. Effective delivery of nucleic acids is crucial to their successful biological application. The microvesicles/exosomes appear to be naturally produced membraneous structures in the interstitial fluid with the ability to be recognized, adhered to and fused with other cell types, ultimately to deliver the cargo to target cells and their nuclei.

We confirm that microvesicles/exosomes derived from cardiomyocytes [42] are representative members of the exosome family. It should however be pointed out that the microvesicle/exosome population observed was not homogenous. Hence, 80% of microvesicles/exosomes were positive for flotillin-1, while only 30% were positive for caveolin-3. Furthermore some microvesicles/exosomes displayed an electron dense appearance, while others displayed an electron lucent interior. Finally the rather large size distribution is notable. These signs of heterogeneity in appearance and surface antigens could imply differences in cargo as well as target cells. The finding of caveolin-3, which is the myocyte specific isoform of caveolins, in 30% of the microvesicles/exosomes reflects the relationship to cardiomyocytes, since this isoform is exclusively expressed in skeletal and cardiac muscle [43]. Caveolin-3 forms caveolae in muscle tissue that have a key role in the maintenance of plasma membrane integrity and in the processes of vesicular trafficking and signal transduction [44]. To date, there have been 30 caveolin-3 mutations identified in patients and one caveolin-3 mutant has been described in a case of hypertrophic cardiomyopathy [44].

Enzymes DNase I and II present in extracellular fluid (including blood plasma) degrade efficiently DNA and therefore only trace amounts of free (i.e. not shielded from an enzymatic attack) DNA can normally be detected in extracellular fluids. Accordingly, transfer of genetic information from one cell to another must be protected from DNase attack. We suggest that in case of cardiomyocyte intercellular communication this is accomplished by microvesicle/exosome-shielding of DNA.

The functional transfer of genetic information to target cells was in fact substantiated by clearcut down/up-regulation of gene products. This supports the idea that the content of microvesicles/exosomes (i.a. DNA, mRNA and proteins) was indeed internalised in fibroblasts rather than just adhered to them. This internalisation even into the nucleus was corroborated by the confocal microscopy findings.

In summary, we have demonstrated the presence of cardiomyocyte derived, nucleic acid-containing microvesicles/exosomes in media of cultured cardiomyocytes. We suggest that exosomes derived from cardiomyocytes could be denoted “cardiosomes”. Microvesicles and cardiosomes can be involved in a metabolic course of events in the microenvironment of the heart by facilitating an array of cellular processes through transfer of nucleic acids to target cells and their nuclei.

## Supporting Information

Figure S1 **A biological network describing tentative interactions of proteins encoded by mRNA detected in microvesicles/exosomes.** Total RNA was prepared from microvesicles/exosomes. Illumina Beadstation was used to identify mRNA. Genes with detection p-value less than 0.01 and signal levels over 50 were considered as significantly detected. Genes/proteins, for which identified mRNAs are coding, were used in the bioinformatic database MetaCore (GeneGo Inc.) to construct a biological network. Out of 1520 detected genes in the microvesicles/exosomes, 423 could be directly connected to a biological network without the addition of any extra genes/proteins.
(TIF)

Figure S2 **Biological network of ribosomal genes/proteins.** Total RNA was prepared from microvesicles/exosomes. Illumina Beadstation was used to identify mRNA. Genes with detection p-value less than 0.01 and signal levels over 50 were considered as significantly detected. The bioinformatic database MetaCore (GeneGo Inc.) was used to analyze the 1520 detected genes in the microvesicles/exosomes. Thirty-five genes coding for proteins in the small and large ribosomal subunit and eight additional genes could be connected in a biological network (red circles).
(TIF)

Figure S3 **Biological network of mitochondrial genes/proteins.** Total RNA was prepared from microvesicles/exosomes. Illumina Beadstation was used to identify mRNA. Genes with detection p-value less than 0.01 and signal levels over 50 were considered as significantly detected. The bioinformatic database MetaCore (GeneGo Inc.) was used to analyze the 1520 detected genes in the microvesicles/exosomes. Thirty-three genes coding for proteins in the mitochondria could be detected (red circles).
(TIF)

Table S1 **Genes corresponding to DNA sequences detected in exosomes.** DNA was prepared by conventional methods. By use of *i.a.* TDT polymerase, poly A was added at the end of the DNA strand and a cDNA was constructed. This was labelled with biotin and converted to cRNA therewith making it adaptable to a commercial chip. Hybridisation revealed 343 different chromosomal DNA sequences when using a P-value less than 0.01 and a signal power more than 50.
(DOC)

Table S2 **Interactions between genes/proteins coded by exosomal mRNA.** Total RNA was extracted from the microvesicles/exosomes. Genes/proteins (for which identified mRNAs are encoding) were used in the bioinformatic data base and a biological network was drawn. The microvesicles/exosomes contained 1595 detected mRNA of which 1520 also were detected in cardiomyocytes. Out of these 1520 detected mRNAs in the microvesicles/exosomes, 423 could be directly connected to a biological network without addition of any extra genes/proteins.
(DOC)

Table S3 **Differentially expressed genes in Fb after exosome incubation.** Fibroblasts were transfected with microvesicles/exosomes and a clearcut effect on gene expression was found. After ultracentrifugation the microvesicle/exosome-enriched pellet resuspended in fresh DMEM that induced about 70% more changes (65 up and 96 down), here listed, than the microvesicle/exosome-deficient supernatant that induced only 96 changes (21 up, 75 down) when incubated with the fibroblast for 48 h.
(DOC)
