# Supplementary figures and images for: Cardiomyocyte Microvesicles Contain DNA/RNA and Convey Biological Messages to Target Cells
Source: PLoS One. 2012 Apr 10;7(4):e34653. doi: 10.1371/journal.pone.0034653 (PMC3323564; doi:10.1371/journal.pone.0034653)

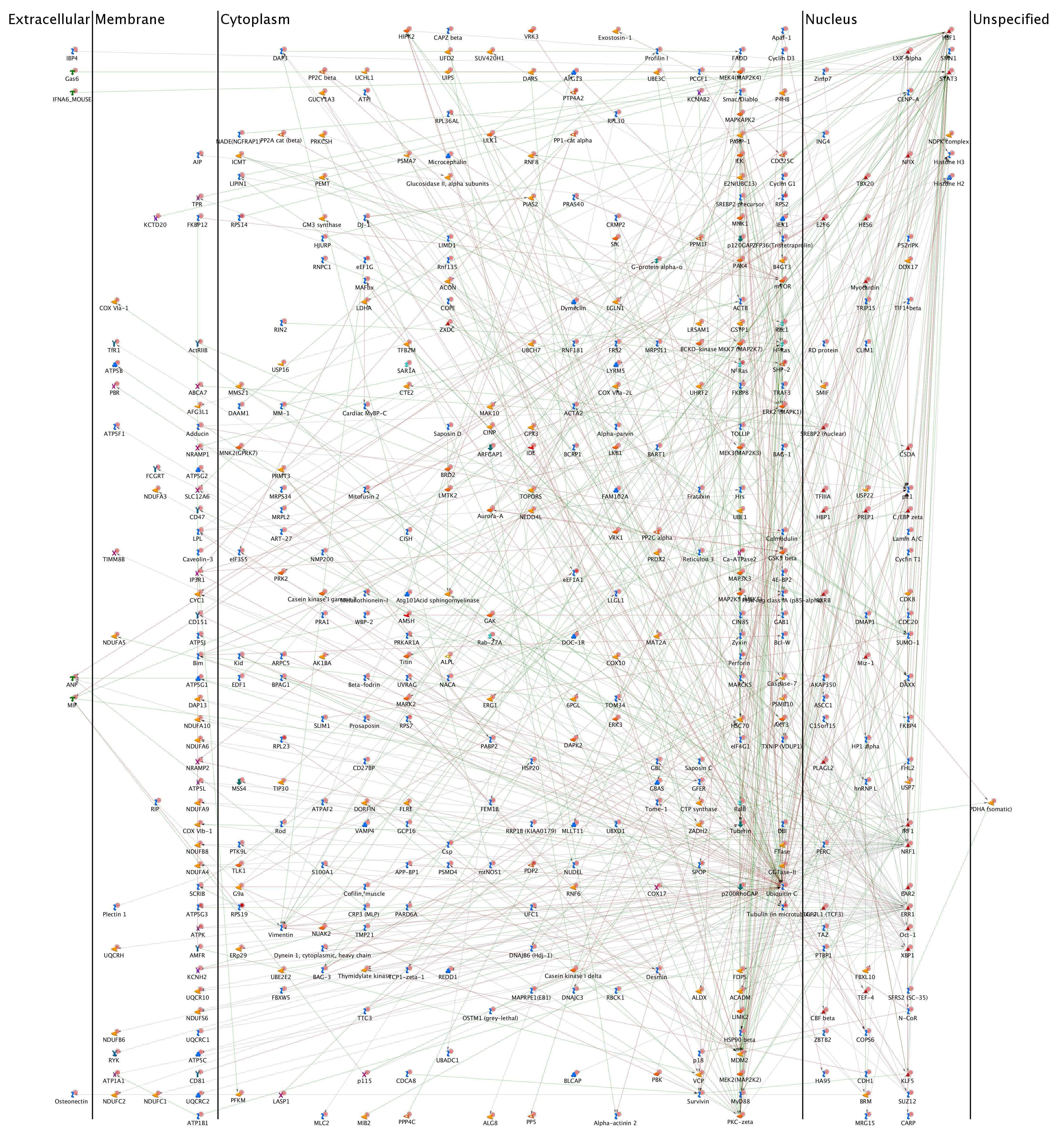

Supplement: Figure S1 — A biological network describing tentative interactions of proteins encoded by mRNA detected in microvesicles/exosomes. Total RNA was prepared from microvesicles/exosomes. Illumina Beadstation was used to identify mRNA. Genes with detection p-value less than 0.01 and signal levels over 50 were considered as significantly detected. Genes/proteins, for which identified mRNAs are coding, were used in the bioinformatic database MetaCore (GeneGo Inc.) to construct a biological network. Out of 1520 detected genes in the microvesicles/exosomes, 423 could be directly connected to a biological network without the addition of any extra genes/proteins. (TIF) [file pone.0034653.s001.tif]

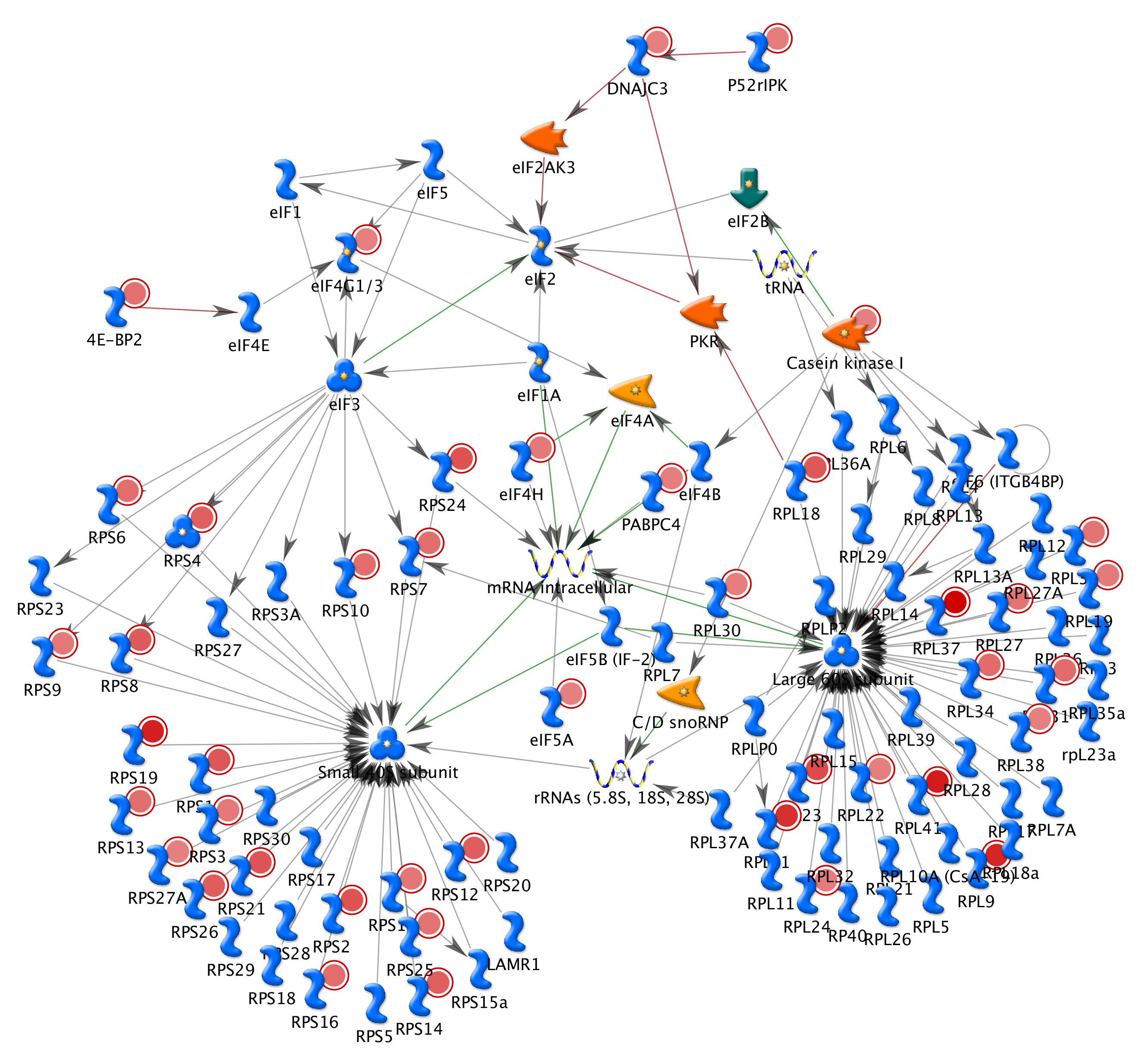

Supplement: Figure S2 — Biological network of ribosomal genes/proteins. Total RNA was prepared from microvesicles/exosomes. Illumina Beadstation was used to identify mRNA. Genes with detection p-value less than 0.01 and signal levels over 50 were considered as significantly detected. The bioinformatic database MetaCore (GeneGo Inc.) was used to analyze the 1520 detected genes in the microvesicles/exosomes. Thirty-five genes coding for proteins in the small and large ribosomal subunit and eight additional genes could be connected in a biological network (red circles). (TIF) [file pone.0034653.s002.tif]

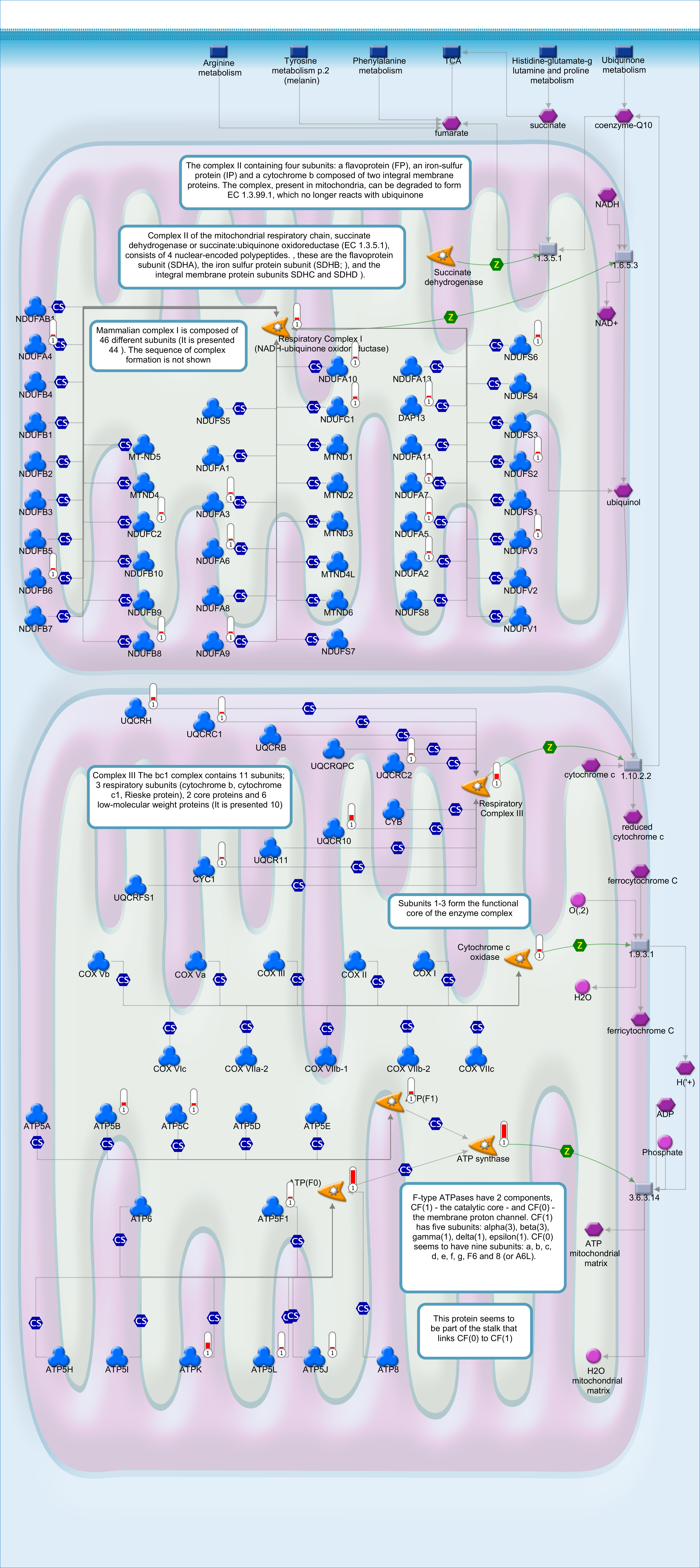

Supplement: Figure S3 — Biological network of mitochondrial genes/proteins. Total RNA was prepared from microvesicles/exosomes. Illumina Beadstation was used to identify mRNA. Genes with detection p-value less than 0.01 and signal levels over 50 were considered as significantly detected. The bioinformatic database MetaCore (GeneGo Inc.) was used to analyze the 1520 detected genes in the microvesicles/exosomes. Thirty-three genes coding for proteins in the mitochondria could be detected (red circles). (TIF) [file pone.0034653.s003.tif]
