# Supplementary material for: Cardiomyocyte Microvesicles Contain DNA/RNA and Convey Biological Messages to Target Cells
Source: PLoS One. 2012 Apr 10;7(4):e34653. doi: 10.1371/journal.pone.0034653 (PMC3323564; doi:10.1371/journal.pone.0034653)
Supplement: Table S1 — Genes corresponding to DNA sequences detected in exosomes. DNA was prepared by conventional methods. By use of i.a. TDT polymerase, poly A was added at the end of the DNA strand and a cDNA was constructed. This was labelled with biotin and converted to cRNA therewith making it adaptable to a commercial chip. Hybridisation revealed 343 different chromosomal DNA sequences when using a P-value less than 0.01 and a signal power more than 50. (DOC) [file pone.0034653.s004.doc]

Supplemental Table 1

| **Genes corresponding to DNA sequences detected in exosomes** | | | |
| --- | --- | --- | --- |
| **SYMBOL** | **DEFINITION** | **ENTREZ_GENE_ID** | **ONTOLOGY_FUNCTION** |
| 1110002B05Rik | RIKEN cDNA 1110002B05 gene (1110002B05Rik), mRNA. | 104725 |  |
| 1110028A07Rik | RIKEN cDNA 1110028A07 gene (1110028A07Rik), mRNA. | 68680 |  |
| 1700047I17Rik1 | RIKEN cDNA 1700047I17 gene 1 (1700047I17Rik1), mRNA. | 73385 |  |
| 1810007P19Rik | RIKEN cDNA 1810007P19 gene (1810007P19Rik), mRNA. | 230751 |  |
| 1810064F22Rik | RIKEN cDNA 1810064F22 gene (1810064F22Rik), mRNA. | 69862 |  |
| 2610208M17Rik | RIKEN cDNA 2610208M17 gene (2610208M17Rik), mRNA. | 108909 |  |
| 2610305D13Rik | RIKEN cDNA 2610305D13 gene (2610305D13Rik), mRNA. | 112422 | Interacting selectively with any metal ion [goid 46872] [evidence IEA]; Interacting selectively with zinc (Zn) ions [goid 8270] [evidence IEA] |
| 2700062C07Rik | RIKEN cDNA 2700062C07 gene (2700062C07Rik), mRNA. | 68046 |  |
| 2700081O15Rik | RIKEN cDNA 2700081O15 gene (2700081O15Rik), mRNA. | 108899 |  |
| 4632417K18Rik | RIKEN cDNA 4632417K18 gene (4632417K18Rik), mRNA. | 107373 | Catalysis of the hydrolysis of a peptide bond. A peptide bond is a covalent bond formed when the carbon atom from the carboxyl group of one amino acid shares electrons with the nitrogen atom from the amino group of a second amino acid [goid 8233] [evidence IEA] |
| 4930470P17Rik | PREDICTED: RIKEN cDNA 4930470P17 gene (4930470P17Rik), misc RNA. | 67637 |  |
| 4931406P16Rik | RIKEN cDNA 4931406P16 gene (4931406P16Rik), mRNA. | 233103 |  |
| 4933411G11Rik | RIKEN cDNA 4933411G11Rik gene (4933411G11Rik), mRNA. | 330228 |  |
| 5430437P03Rik | RIKEN cDNA 5430437P03 gene (5430437P03Rik), mRNA. | 68251 |  |
| 5730593F17Rik | RIKEN cDNA 5730593F17 gene (5730593F17Rik), mRNA. | 215512 |  |
| 6030408B16Rik | RIKEN cDNA 6030408B16 gene (6030408B16Rik), mRNA. | 77717 |  |
| 6430571L13Rik | RIKEN cDNA 6430571L13 gene (6430571L13Rik), mRNA. | 235599 |  |
| 6530418L21Rik | RIKEN cDNA 6530418L21 gene (6530418L21Rik), mRNA. | 109050 |  |
| 9130011E15Rik | RIKEN cDNA 9130011E15 gene (9130011E15Rik), mRNA. | 71617 |  |
| A530088H08Rik | RIKEN cDNA A530088H08 gene (A530088H08Rik), mRNA. | 193003 |  |
| AA792892 | expressed sequence AA792892 (AA792892), mRNA. | 100554 | Elemental activities, such as catalysis or binding, describing the actions of a gene product at the molecular level. A given gene product may exhibit one or more molecular functions [goid 3674] [evidence ND ] |
| Abcf2 | ATP-binding cassette, sub-family F (GCN20), member 2 (Abcf2), nuclear gene encoding mitochondrial protein, mRNA. | 27407 | Interacting selectively with ATP, adenosine 5'-triphosphate, a universally important coenzyme and enzyme regulator [goid 5524] [evidence IEA]; Catalysis of the reaction: a nucleoside triphosphate + H2O = nucleoside diphosphate + phosphate [goid 17111] [evidence IEA]; Interacting selectively with a nucleotide, any compound consisting of a nucleoside that is esterified with (ortho)phosphate or an oligophosphate at any hydroxyl group on the ribose or deoxyribose moiety [goid 166] [evidence IEA]; Catalysis of the reaction: ATP + H2O = ADP + phosphate. May or may not be coupled to another reaction [goid 16887] [evidence IEA] |
| Abcf3 | ATP-binding cassette, sub-family F (GCN20), member 3 (Abcf3), mRNA. | 27406 | Interacting selectively with ATP, adenosine 5'-triphosphate, a universally important coenzyme and enzyme regulator [goid 5524] [evidence IEA]; Interacting selectively with a nucleotide, any compound consisting of a nucleoside that is esterified with (ortho)phosphate or an oligophosphate at any hydroxyl group on the ribose or deoxyribose moiety [goid 166] [evidence IEA]; Catalysis of the reaction: a nucleoside triphosphate + H2O = nucleoside diphosphate + phosphate [goid 17111] [evidence IEA]; Catalysis of the reaction: ATP + H2O = ADP + phosphate. May or may not be coupled to another reaction [goid 16887] [evidence IEA] |
| Acpp | acid phosphatase, prostate (Acpp), transcript variant 1, mRNA. | 56318 | Catalysis of the reaction: an orthophosphoric monoester + H2O = an alcohol + phosphate, with an acid pH optimum [goid 3993] [evidence ISS] |
| Acsl5 | acyl-CoA synthetase long-chain family member 5 (Acsl5), mRNA. | 433256 | Catalysis of a biochemical reaction at physiological temperatures. In biologically catalyzed reactions, the reactants are known as substrates, and the catalysts are naturally occurring macromolecular substances known as enzymes. Enzymes possess specific binding sites for substrates, and are usually composed wholly or largely of protein, but RNA that has catalytic activity (ribozyme) is often also regarded as enzymatic [goid 3824] [evidence IEA]; Interacting selectively with magnesium (Mg) ions [goid 287] [evidence IEA]; Catalysis of the ligation of two substances with concomitant breaking of a diphosphate linkage, usually in a nucleoside triphosphate. Ligase is the systematic name for any enzyme of EC class 6 [goid 16874] [evidence IEA]; Catalysis of the reaction: ATP + a long-chain carboxylic acid + CoA = AMP + diphosphate + an acyl-CoA; long-chain fatty acids have chain lengths of C12-18 [goid 4467] [evidence IEA] |
| Acyp2 | acylphosphatase 2, muscle type (Acyp2), mRNA. | 75572 | Catalysis of the hydrolysis of various bonds, e.g. C-O, C-N, C-C, phosphoric anhydride bonds, etc. Hydrolase is the systematic name for any enzyme of EC class 3 [goid 16787] [evidence IEA]; Catalysis of the reaction: an acyl phosphate + H2O = a carboxylate + phosphate [goid 3998] [evidence IEA] |
| Aff1 | AF4/FMR2 family, member 1 (Aff1), transcript variant 1, mRNA. | 17355 | Catalysis of the transfer of a phosphate group, usually from ATP, to a substrate molecule [goid 16301] [evidence IEA]; Any transcription regulator activity required for initiation or upregulation of transcription [goid 16563] [evidence IDA]; The function of binding to a specific DNA sequence in order to modulate transcription. The transcription factor may or may not also interact selectively with a protein or macromolecular complex [goid 3700] [evidence TAS] |
| Ahsg | alpha-2-HS-glycoprotein (Ahsg), mRNA. | 11625 | [goid 4869] [evidence IEA] |
| AI467606 | expressed sequence AI467606 (AI467606), mRNA. | 101602 |  |
| Akap9 | A kinase (PRKA) anchor protein (yotiao) 9 (Akap9), mRNA. | 100986 | Catalysis of the transfer of a phosphate group, usually from ATP, to a substrate molecule [goid 16301] [evidence IEA]; Interacting selectively with any protein or protein complex (a complex of two or more proteins that may include other nonprotein molecules) [goid 5515] [evidence IPI] |
| Ars2 | arsenate resistance protein 2 (Ars2), mRNA. | 83701 | Elemental activities, such as catalysis or binding, describing the actions of a gene product at the molecular level. A given gene product may exhibit one or more molecular functions [goid 3674] [evidence ND ] |
| Ascc2 | activating signal cointegrator 1 complex subunit 2 (Ascc2), mRNA. | 75452 |  |
| Atp5a1 | ATP synthase, H+ transporting, mitochondrial F1 complex, alpha subunit, isoform 1 (Atp5a1), nuclear gene encoding mitochondrial protein, mRNA. | 11946 | Interacting selectively with any metal ion [goid 46872] [evidence IEA]; Catalysis of the transfer of a solute or solutes from one side of a membrane to the other according to the reaction: ATP + H2O + H+(in) = ADP + phosphate + H+(out), by a rotational mechanism [goid 46961] [evidence IEA]; Catalysis of the transfer of a solute or solutes from one side of a membrane to the other according to the reaction: ADP + phosphate = ATP + H2O, coupled with transport of H+ down a concentration gradient, by a rotational mechanism [goid 46933] [evidence IMP]; Interacting selectively with a nucleotide, any compound consisting of a nucleoside that is esterified with (ortho)phosphate or an oligophosphate at any hydroxyl group on the ribose or deoxyribose moiety [goid 166] [evidence IEA]; Catalysis of the transfer of hydrogen ions from one side of a membrane to the other [goid 15078] [evidence IEA]; Catalysis of the hydrolysis of an acid anhydride to directly drive the transport of a substance across a membrane [goid 16820] [evidence IEA]; Interacting selectively with ATP, adenosine 5'-triphosphate, a universally important coenzyme and enzyme regulator [goid 5524] [evidence IMP] |
| Aven | apoptosis, caspase activation inhibitor (Aven), mRNA. | 74268 | Interacting selectively with any protein or protein complex (a complex of two or more proteins that may include other nonprotein molecules) [goid 5515] [evidence ISS] |
| Avpr1a | arginine vasopressin receptor 1A (Avpr1a), mRNA. | 54140 | A receptor that binds an extracellular ligand and transmits the signal to a heterotrimeric G-protein complex. These receptors are characteristically seven-transmembrane receptors and are made up of hetero- or homodimers [goid 4930] [evidence IEA]; Combining with an extracellular or intracellular messenger to initiate a change in cell activity [goid 4872] [evidence IEA]; Mediates the transfer of a signal from the outside to the inside of a cell by means other than the introduction of the signal molecule itself into the cell [goid 4871] [evidence IEA]; Combining with vasopressin to initiate a change in cell activity [goid 5000] [evidence IEA] |
| B230380D07Rik | RIKEN cDNA B230380D07 gene (B230380D07Rik), mRNA. | 235461 | Catalysis of the transfer of hydrogen ions from one side of a membrane to the other [goid 15078] [evidence IEA] |
| BC017647 | cDNA sequence BC017647 (BC017647), mRNA. | 216971 |  |
| BC020002 | cDNA sequence BC020002 (BC020002), mRNA. | 252875 |  |
| BC021785 | cDNA sequence BC021785 (BC021785), mRNA. | 215928 | Catalysis of the transfer of a solute or solutes from one side of a membrane to the other according to the reaction: sugar(out) + H+(out) = sugar(in) + H+(in) [goid 5351] [evidence IEA] |
| BC026782 | cDNA sequence BC026782 (BC026782), mRNA. | 545366 |  |
| BC030476 | cDNA sequence BC030476 (BC030476), mRNA. | 239368 |  |
| BC030499 | cDNA sequence BC030499 (BC030499), mRNA. | 216976 | Catalysis of the reaction: ATP + a protein serine/threonine = ADP + protein serine/threonine phosphate [goid 4674] [evidence IEA]; Interacting selectively with ATP, adenosine 5'-triphosphate, a universally important coenzyme and enzyme regulator [goid 5524] [evidence IEA]; Interacting selectively with a nucleotide, any compound consisting of a nucleoside that is esterified with (ortho)phosphate or an oligophosphate at any hydroxyl group on the ribose or deoxyribose moiety [goid 166] [evidence IEA]; Catalysis of the phosphorylation of an amino acid residue in a protein, usually according to the reaction: a protein + ATP = a phosphoprotein + ADP [goid 4672] [evidence IEA]; Catalysis of the transfer of a group, e.g. a methyl group, glycosyl group, acyl group, phosphorus-containing, or other groups, from one compound (generally regarded as the donor) to another compound (generally regarded as the acceptor). Transferase is the systematic name for any enzyme of EC class 2 [goid 16740] [evidence IEA]; Catalysis of the transfer of a phosphate group, usually from ATP, to a substrate molecule [goid 16301] [evidence IEA] |
| BC050811 | cDNA sequence BC050811 (BC050811), mRNA. | 241943 |  |
| Btg3 | B-cell translocation gene 3 (Btg3), mRNA. | 12228 | Interacting selectively with any protein or protein complex (a complex of two or more proteins that may include other nonprotein molecules) [goid 5515] [evidence IPI] |
| C4a | complement component 4A (Rodgers blood group) (C4a), mRNA. | 625018 |  |
| C5ar1 | complement component 5a receptor 1 (C5ar1), mRNA. | 12273 | A receptor that binds an extracellular ligand and transmits the signal to a heterotrimeric G-protein complex. These receptors are characteristically seven-transmembrane receptors and are made up of hetero- or homodimers [goid 4930] [evidence IEA]; Combining with an extracellular or intracellular messenger to initiate a change in cell activity [goid 4872] [evidence IEA]; Mediates the transfer of a signal from the outside to the inside of a cell by means other than the introduction of the signal molecule itself into the cell [goid 4871] [evidence IEA]; Combining with anaphylatoxin C5a to initiate a change in cell activity [goid 4944] [evidence IDA]; Combining with anaphylatoxin to initiate a change in cell activity [goid 4942] [evidence IEA] |
| C630028N24Rik | RIKEN cDNA C630028N24 gene (C630028N24Rik), mRNA. | 235386 | Catalysis of the transfer of a group, e.g. a methyl group, glycosyl group, acyl group, phosphorus-containing, or other groups, from one compound (generally regarded as the donor) to another compound (generally regarded as the acceptor). Transferase is the systematic name for any enzyme of EC class 2 [goid 16740] [evidence IEA]; Catalysis of the transfer of a phosphate group, usually from ATP, to a substrate molecule [goid 16301] [evidence IEA] |
| Calcoco1 | calcium binding and coiled coil domain 1 (Calcoco1), mRNA. | 67488 | The function of a transcription cofactor that activates transcription from a RNA polymerase II promoter; does not bind DNA itself [goid 3713] [evidence IDA]; Interacting selectively with any protein or protein complex (a complex of two or more proteins that may include other nonprotein molecules) [goid 5515] [evidence IPI]; Interacting selectively with chromatin, the network of fibers of DNA and protein that make up the chromosomes of the eukaryotic nucleus during interphase [goid 3682] [evidence IDA] |
| Calr3 | calreticulin 3 (Calr3), transcript variant 1, mRNA. | 73316 | Interacting selectively with calcium ions (Ca2+) [goid 5509] [evidence IEA]; Interacting selectively with an unfolded protein [goid 51082] [evidence IEA] |
| Calr4 | calreticulin 4 (Calr4), mRNA. | 108802 | Elemental activities, such as catalysis or binding, describing the actions of a gene product at the molecular level. A given gene product may exhibit one or more molecular functions [goid 3674] [evidence ND ] |
| Car4 | carbonic anhydrase 4 (Car4), mRNA. | 12351 | Catalysis of the cleavage of C-C, C-O, C-N and other bonds by other means than by hydrolysis or oxidation, or conversely adding a group to a double bond. They differ from other enzymes in that two substrates are involved in one reaction direction, but only one in the other direction. When acting on the single substrate, a molecule is eliminated and this generates either a new double bond or a new ring [goid 16829] [evidence IEA]; Interacting selectively with zinc (Zn) ions [goid 8270] [evidence IEA]; Interacting selectively with any metal ion [goid 46872] [evidence IEA]; Catalysis of the reaction: H2CO3 = CO2 + H2O [goid 4089] [evidence IEA] |
| Cbfa2t3h | core-binding factor, runt domain, alpha subunit 2, translocated to, 3 homolog (human) (Cbfa2t3h), mRNA. | 12398 |  |
| Cbln2 | cerebellin 2 precursor protein (Cbln2), mRNA. | 12405 | Elemental activities, such as catalysis or binding, describing the actions of a gene product at the molecular level. A given gene product may exhibit one or more molecular functions [goid 3674] [evidence ND ] |
| Ccdc65 | coiled-coil domain containing 65 (Ccdc65), mRNA. | 105833 |  |
| Ccdc79 | coiled-coil domain containing 79 (Ccdc79), mRNA. | 320022 | The selective, often stoichiometric, interaction of a molecule with one or more specific sites on another molecule [goid 5488] [evidence IEA]; Interacting selectively with DNA (deoxyribonucleic acid) [goid 3677] [evidence IEA] |
| Ccl11 | small chemokine (C-C motif) ligand 11 (Ccl11), mRNA. | 20292 | Functions to control the survival, growth, differentiation and effector function of tissues and cells [goid 5125] [evidence IEA]; The function of a family of chemotactic pro-inflammatory activation-inducible cytokines acting primarily upon hemopoietic cells in immunoregulatory processes; all chemokines possess a number of conserved cysteine residues involved in intramolecular disulfide bond formation [goid 8009] [evidence IEA] |
| Cd160 | CD160 antigen (Cd160), mRNA. | 54215 | Combining with an extracellular or intracellular messenger to initiate a change in cell activity [goid 4872] [evidence IEA] |
| Cd1d1 | CD1d1 antigen (Cd1d1), mRNA. | 12479 | Interacting selectively with any protein or protein complex (a complex of two or more proteins that may include other nonprotein molecules) [goid 5515] [evidence IPI]; Interacting selectively with an endogenous cellular lipid antigen [goid 30883] [evidence IDA] |
| Cd247 | CD247 antigen (Cd247), mRNA. | 12503 | Combining with an extracellular or intracellular messenger to initiate a change in cell activity [goid 4872] [evidence IEA]; Combining with an extracellular or intracellular messenger to initiate a change in cell activity, and spanning to the membrane of either the cell or an organelle [goid 4888] [evidence IEA]; Interacting selectively with any protein or protein complex (a complex of two or more proteins that may include other nonprotein molecules) [goid 5515] [evidence ISO]; Interacting selectively with any protein or protein complex (a complex of two or more proteins that may include other nonprotein molecules) [goid 5515] [evidence IPI] |
| Cdcp2 | CUB domain containing protein 2 (Cdcp2), mRNA. | 242603 |  |
| Cdon | cell adhesion molecule-related/down-regulated by oncogenes (Cdon), mRNA. | 57810 | Interacting selectively with any protein or protein complex (a complex of two or more proteins that may include other nonprotein molecules) [goid 5515] [evidence IPI] |
| Ceacam20 | CEA-related cell adhesion molecule 20 (Ceacam20), mRNA. | 71601 |  |
| Chka | choline kinase alpha (Chka), transcript variant 2, mRNA. | 12660 | Catalysis of the transfer of a group, e.g. a methyl group, glycosyl group, acyl group, phosphorus-containing, or other groups, from one compound (generally regarded as the donor) to another compound (generally regarded as the acceptor). Transferase is the systematic name for any enzyme of EC class 2 [goid 16740] [evidence IEA]; Catalysis of the transfer of a phosphate group, usually from ATP, to a substrate molecule [goid 16301] [evidence IEA]; Catalysis of the reaction: ATP + ethanolamine = ADP + O-phosphoethanolamine [goid 4305] [evidence IEA]; Catalysis of the reaction: ATP + choline = ADP + O-phosphocholine [goid 4103] [evidence IDA]; Catalysis of the reaction: an acylcholine + H2O = choline + a carboxylic acid anion [goid 4104] [evidence IDA]; Interacting selectively with an identical protein to form a homodimer [goid 42803] [evidence IPI] |
| Cldn11 | claudin 11 (Cldn11), mRNA. | 18417 | Interacting selectively with any protein or protein complex (a complex of two or more proteins that may include other nonprotein molecules) [goid 5515] [evidence IPI]; The action of a molecule that contributes to the structural integrity of a complex or assembly within or outside a cell [goid 5198] [evidence IEA] |
| Cldn14 | claudin 14 (Cldn14), mRNA. | 56173 | The action of a molecule that contributes to the structural integrity of a complex or assembly within or outside a cell [goid 5198] [evidence IEA] |
| Clns1a | chloride channel, nucleotide-sensitive, 1A (Clns1a), mRNA. | 12729 |  |
| Cmklr1 | chemokine-like receptor 1 (Cmklr1), mRNA. | 14747 | A receptor that binds an extracellular ligand and transmits the signal to a heterotrimeric G-protein complex. These receptors are characteristically seven-transmembrane receptors and are made up of hetero- or homodimers [goid 4930] [evidence IEA]; Combining with an extracellular or intracellular messenger to initiate a change in cell activity [goid 4872] [evidence IEA]; Mediates the transfer of a signal from the outside to the inside of a cell by means other than the introduction of the signal molecule itself into the cell [goid 4871] [evidence IEA]; Combining with anaphylatoxin C5a to initiate a change in cell activity [goid 4944] [evidence IEA] |
| Cnih4 | cornichon homolog 4 (Drosophila) (Cnih4), mRNA. | 98417 |  |
| Col6a2 | procollagen, type VI, alpha 2 (Col6a2), mRNA. | 12834 | The action of a molecule that contributes to the structural integrity of a complex or assembly within or outside a cell [goid 5198] [evidence IEA]; Interacting selectively with any protein or protein complex (a complex of two or more proteins that may include other nonprotein molecules) [goid 5515] [evidence IEA] |
| Cox6a1 | cytochrome c oxidase, subunit VI a, polypeptide 1 (Cox6a1), nuclear gene encoding mitochondrial protein, mRNA. | 12861 | Catalysis of the transfer of a solute or solutes from one side of a membrane to the other according to the reaction: 4 ferrocytochrome c + O2 = 4 ferricytochrome c + 2 H2O [goid 4129] [evidence IEA]; Any molecular entity that serves as an electron acceptor and electron donor in an electron transport system [goid 9055] [evidence IEA] |
| Cpt2 | carnitine palmitoyltransferase 2 (Cpt2), mRNA. | 12896 | Catalysis of the generalized reaction: acyl-carrier + reactant = acyl-reactant + carrier [goid 8415] [evidence IEA]; Catalysis of the transfer of a group, e.g. a methyl group, glycosyl group, acyl group, phosphorus-containing, or other groups, from one compound (generally regarded as the donor) to another compound (generally regarded as the acceptor). Transferase is the systematic name for any enzyme of EC class 2 [goid 16740] [evidence IEA]; Catalysis of the reaction: palmitoyl-CoA + L-carnitine = CoA + L-palmitoylcarnitine [goid 4095] [evidence IEA] |
| Cpxm1 | carboxypeptidase X 1 (M14 family) (Cpxm1), mRNA. | 56264 | Catalysis of the hydrolysis of various bonds, e.g. C-O, C-N, C-C, phosphoric anhydride bonds, etc. Hydrolase is the systematic name for any enzyme of EC class 3 [goid 16787] [evidence IEA]; Interacting selectively with zinc (Zn) ions [goid 8270] [evidence IEA]; Catalysis of the hydrolysis of a peptide bond. A peptide bond is a covalent bond formed when the carbon atom from the carboxyl group of one amino acid shares electrons with the nitrogen atom from the amino group of a second amino acid [goid 8233] [evidence IEA]; Interacting selectively with any metal ion [goid 46872] [evidence IEA]; Catalysis of the hydrolysis of peptide bonds by a mechanism in which water acts as a nucleophile, one or two metal ions hold the water molecule in place, and charged amino acid side chains are ligands for the metal ions [goid 8237] [evidence IEA]; Catalysis of the hydrolysis of the terminal or penultimate peptide bond at the C-terminal end of a peptide or polypeptide [goid 4180] [evidence IDA]; Catalysis of the hydrolysis of C-terminal amino acid residues from a polypeptide chain by a mechanism in which water acts as a nucleophile, one or two metal ions hold the water molecule in place, and charged amino acid side chains are ligands for the metal ions [goid 4181] [evidence IEA] |
| Csnk2a1 | casein kinase 2, alpha 1 polypeptide (Csnk2a1), mRNA. | 12995 | Interacting selectively with a nucleotide, any compound consisting of a nucleoside that is esterified with (ortho)phosphate or an oligophosphate at any hydroxyl group on the ribose or deoxyribose moiety [goid 166] [evidence IEA]; Catalysis of the reaction: ATP + a protein serine/threonine = ADP + protein serine/threonine phosphate [goid 4674] [evidence IEA]; Catalysis of the transfer of a group, e.g. a methyl group, glycosyl group, acyl group, phosphorus-containing, or other groups, from one compound (generally regarded as the donor) to another compound (generally regarded as the acceptor). Transferase is the systematic name for any enzyme of EC class 2 [goid 16740] [evidence IEA]; Interacting selectively with ATP, adenosine 5'-triphosphate, a universally important coenzyme and enzyme regulator [goid 5524] [evidence IEA]; Catalysis of the phosphorylation of an amino acid residue in a protein, usually according to the reaction: a protein + ATP = a phosphoprotein + ADP [goid 4672] [evidence IEA]; Catalysis of the transfer of a phosphate group, usually from ATP, to a substrate molecule [goid 16301] [evidence IDA]; Interacting selectively with any protein or protein complex (a complex of two or more proteins that may include other nonprotein molecules) [goid 5515] [evidence IPI]; Modulation of the activity of a protein phosphatase enzyme [goid 19888] [evidence IDA]; Interacting selectively with the beta subunit of the catenin complex [goid 8013] [evidence IPI] |
| Csrnp2 | cysteine-serine-rich nuclear protein 2 (Csrnp2), mRNA. | 207785 | The function of binding to a specific DNA sequence in order to modulate transcription. The transcription factor may or may not also interact selectively with a protein or macromolecular complex [goid 3700] [evidence IDA]; Interacting selectively with DNA (deoxyribonucleic acid) [goid 3677] [evidence IEA] |
| Ctcfl | CCCTC-binding factor (zinc finger protein)-like (Ctcfl), mRNA. | 664799 |  |
| Ctdp1 | CTD (carboxy-terminal domain, RNA polymerase II, polypeptide A) phosphatase, subunit 1 (Ctdp1), mRNA. | 67655 | Catalysis of the hydrolysis of various bonds, e.g. C-O, C-N, C-C, phosphoric anhydride bonds, etc. Hydrolase is the systematic name for any enzyme of EC class 3 [goid 16787] [evidence IEA]; Catalysis of the reaction: a phosphoprotein + H2O = a protein + phosphate. Together with protein kinases, these enzymes control the state of phosphorylation of cell proteins and thereby provide an important mechanism for regulating cellular activity [goid 4721] [evidence IEA]; Interacting selectively with any protein or protein complex (a complex of two or more proteins that may include other nonprotein molecules) [goid 5515] [evidence IPI] |
| Ctsw | cathepsin W (Ctsw), mRNA. | 13041 | Catalysis of the hydrolysis of various bonds, e.g. C-O, C-N, C-C, phosphoric anhydride bonds, etc. Hydrolase is the systematic name for any enzyme of EC class 3 [goid 16787] [evidence IEA]; Catalysis of the hydrolysis of a peptide bond. A peptide bond is a covalent bond formed when the carbon atom from the carboxyl group of one amino acid shares electrons with the nitrogen atom from the amino group of a second amino acid [goid 8233] [evidence IEA]; Catalysis of the hydrolysis of peptide bonds in a polypeptide chain by a mechanism in which the sulfhydryl group of a cysteine residue at the active center acts as a nucleophile [goid 8234] [evidence IEA]; Catalysis of the hydrolysis of internal, alpha-peptide bonds in a polypeptide chain by a mechanism in which the sulfhydryl group of a cysteine residue at the active center acts as a nucleophile [goid 4197] [evidence IEA] |
| Cxcr5 | chemochine (C-X-C motif) receptor 5 (Cxcr5), mRNA. | 12145 | A receptor that binds an extracellular ligand and transmits the signal to a heterotrimeric G-protein complex. These receptors are characteristically seven-transmembrane receptors and are made up of hetero- or homodimers [goid 4930] [evidence IEA]; Combining with an extracellular or intracellular messenger to initiate a change in cell activity [goid 4872] [evidence IEA]; Mediates the transfer of a signal from the outside to the inside of a cell by means other than the introduction of the signal molecule itself into the cell [goid 4871] [evidence IEA]; [goid 4945] [evidence IEA]; Combining with a C-X-C chemokine to initiate a change in cell activity. A C-X-C chemokine has a single amino acid between the first two cysteines of the characteristic four cysteine motif [goid 16494] [evidence IEA] |
| Cyba | cytochrome b-245, alpha polypeptide (Cyba), mRNA. | 13057 | Interacting selectively with iron (Fe) ions [goid 5506] [evidence IEA]; Interacting selectively with any metal ion [goid 46872] [evidence IEA]; Catalysis of an oxidation-reduction (redox) reaction, a reversible chemical reaction in which the oxidation state of an atom or atoms within a molecule is altered. One substrate acts as a hydrogen or electron donor and becomes oxidized, while the other acts as hydrogen or electron acceptor and becomes reduced [goid 16491] [evidence IEA]; Interacting selectively with heme, any compound of iron complexed in a porphyrin (tetrapyrrole) ring [goid 20037] [evidence IEA] |
| Cyp2a5 | cytochrome P450, family 2, subfamily a, polypeptide 5 (Cyp2a5), mRNA. | 13087 | Catalysis of the incorporation of one atom from molecular oxygen into a compound and the reduction of the other atom of oxygen to water [goid 4497] [evidence IEA]; Interacting selectively with iron (Fe) ions [goid 5506] [evidence IEA]; Interacting selectively with any metal ion [goid 46872] [evidence IEA]; Catalysis of an oxidation-reduction (redox) reaction, a reversible chemical reaction in which the oxidation state of an atom or atoms within a molecule is altered. One substrate acts as a hydrogen or electron donor and becomes oxidized, while the other acts as hydrogen or electron acceptor and becomes reduced [goid 16491] [evidence IEA]; Any molecular entity that serves as an electron acceptor and electron donor in an electron transport system [goid 9055] [evidence IEA]; Interacting selectively with heme, any compound of iron complexed in a porphyrin (tetrapyrrole) ring [goid 20037] [evidence IEA]; Catalysis of an oxidation-reduction (redox) reaction in which hydrogen or electrons are transferred from reduced flavin or flavoprotein and one other donor, and one atom of oxygen is incorporated into one donor [goid 16712] [evidence IEA]; Catalysis of the reaction: RH + reduced flavoprotein + O2 = ROH + oxidized flavoprotein + H2O [goid 50381] [evidence IEA] |
| Cyp2j11 | cytochrome P450, family 2, subfamily j, polypeptide 11 (Cyp2j11), mRNA. | 100066 | Interacting selectively with iron (Fe) ions [goid 5506] [evidence IEA]; Interacting selectively with any metal ion [goid 46872] [evidence IEA]; Catalysis of the incorporation of one atom from molecular oxygen into a compound and the reduction of the other atom of oxygen to water [goid 4497] [evidence IEA]; Catalysis of an oxidation-reduction (redox) reaction, a reversible chemical reaction in which the oxidation state of an atom or atoms within a molecule is altered. One substrate acts as a hydrogen or electron donor and becomes oxidized, while the other acts as hydrogen or electron acceptor and becomes reduced [goid 16491] [evidence IEA]; Elemental activities, such as catalysis or binding, describing the actions of a gene product at the molecular level. A given gene product may exhibit one or more molecular functions [goid 3674] [evidence ND ] |
| Cyp3a13 | cytochrome P450, family 3, subfamily a, polypeptide 13 (Cyp3a13), mRNA. | 13113 | Catalysis of the incorporation of one atom from molecular oxygen into a compound and the reduction of the other atom of oxygen to water [goid 4497] [evidence IEA]; Interacting selectively with iron (Fe) ions [goid 5506] [evidence IEA]; Interacting selectively with any metal ion [goid 46872] [evidence IEA]; Catalysis of an oxidation-reduction (redox) reaction, a reversible chemical reaction in which the oxidation state of an atom or atoms within a molecule is altered. One substrate acts as a hydrogen or electron donor and becomes oxidized, while the other acts as hydrogen or electron acceptor and becomes reduced [goid 16491] [evidence IEA]; Any molecular entity that serves as an electron acceptor and electron donor in an electron transport system [goid 9055] [evidence IEA]; Interacting selectively with heme, any compound of iron complexed in a porphyrin (tetrapyrrole) ring [goid 20037] [evidence IEA]; Catalysis of an oxidation-reduction (redox) reaction in which hydrogen or electrons are transferred from reduced flavin or flavoprotein and one other donor, and one atom of oxygen is incorporated into one donor [goid 16712] [evidence IEA]; Catalysis of the reaction: RH + reduced flavoprotein + O2 = ROH + oxidized flavoprotein + H2O [goid 50381] [evidence IEA] |
| Cyp4x1 | cytochrome P450, family 4, subfamily x, polypeptide 1 (Cyp4x1), mRNA. | 81906 | Interacting selectively with iron (Fe) ions [goid 5506] [evidence IEA]; Interacting selectively with any metal ion [goid 46872] [evidence IEA]; Elemental activities, such as catalysis or binding, describing the actions of a gene product at the molecular level. A given gene product may exhibit one or more molecular functions [goid 3674] [evidence ND ]; Catalysis of the incorporation of one atom from molecular oxygen into a compound and the reduction of the other atom of oxygen to water [goid 4497] [evidence IEA]; Catalysis of an oxidation-reduction (redox) reaction, a reversible chemical reaction in which the oxidation state of an atom or atoms within a molecule is altered. One substrate acts as a hydrogen or electron donor and becomes oxidized, while the other acts as hydrogen or electron acceptor and becomes reduced [goid 16491] [evidence IEA] |
| D130040H23Rik | RIKEN cDNA D130040H23 gene (D130040H23Rik), mRNA. | 211135 |  |
| D1Bwg0212e | DNA segment, Chr 1, Brigham & Women's Genetics 0212 expressed (D1Bwg0212e), mRNA. | 52846 |  |
| D5Ertd579e | DNA segment, Chr 5, ERATO Doi 579, expressed (D5Ertd579e), mRNA. | 320661 | Interacting selectively with ATP, adenosine 5'-triphosphate, a universally important coenzyme and enzyme regulator [goid 5524] [evidence IEA]; Interacting selectively with a nucleotide, any compound consisting of a nucleoside that is esterified with (ortho)phosphate or an oligophosphate at any hydroxyl group on the ribose or deoxyribose moiety [goid 166] [evidence IEA] |
| Dap3 | death associated protein 3 (Dap3), nuclear gene encoding mitochondrial protein, mRNA. | 65111 |  |
| Dap3 | death associated protein 3 (Dap3), nuclear gene encoding mitochondrial protein, mRNA. | 65111 |  |
| Dazl | deleted in azoospermia-like (Dazl), mRNA. | 13164 | Interacting selectively with any protein or protein complex (a complex of two or more proteins that may include other nonprotein molecules) [goid 5515] [evidence IPI]; Interacting selectively with an RNA molecule or a portion thereof [goid 3723] [evidence IEA]; Interacting selectively with the 3' untranslated region of an mRNA molecule [goid 3730] [evidence IDA]; Interacting selectively with any nucleic acid [goid 3676] [evidence IEA]; Interacting selectively with a nucleotide, any compound consisting of a nucleoside that is esterified with (ortho)phosphate or an oligophosphate at any hydroxyl group on the ribose or deoxyribose moiety [goid 166] [evidence IEA]; Any of a group of soluble proteins functioning in the activation of ribosome-mediated translation of mRNA into a polypeptide [goid 8494] [evidence IDA] |
| Ddit3 | DNA-damage inducible transcript 3 (Ddit3), mRNA. | 13198 | Interacting selectively with DNA of a specific nucleotide composition, e.g. GC-rich DNA binding, or with a specific sequence motif or type of DNA e.g. promotor binding or rDNA binding [goid 43565] [evidence IEA]; The formation of a protein dimer, a macromolecular structure consists of two noncovalently associated identical or nonidentical subunits [goid 46983] [evidence IEA]; The function of binding to a specific DNA sequence in order to modulate transcription. The transcription factor may or may not also interact selectively with a protein or macromolecular complex [goid 3700] [evidence IMP]; Interacting selectively with DNA (deoxyribonucleic acid) [goid 3677] [evidence ISO]; Interacting selectively with DNA (deoxyribonucleic acid) [goid 3677] [evidence IDA] |
| Ddx3y | DEAD (Asp-Glu-Ala-Asp) box polypeptide 3, Y-linked (Ddx3y), mRNA. | 26900 | Catalysis of the hydrolysis of various bonds, e.g. C-O, C-N, C-C, phosphoric anhydride bonds, etc. Hydrolase is the systematic name for any enzyme of EC class 3 [goid 16787] [evidence IEA]; Catalysis of the reaction: NTP + H2O = NDP + phosphate to drive the unwinding of a DNA or RNA helix [goid 4386] [evidence IEA]; Interacting selectively with an RNA molecule or a portion thereof [goid 3723] [evidence IEA]; Interacting selectively with a nucleotide, any compound consisting of a nucleoside that is esterified with (ortho)phosphate or an oligophosphate at any hydroxyl group on the ribose or deoxyribose moiety [goid 166] [evidence IEA]; Interacting selectively with ATP, adenosine 5'-triphosphate, a universally important coenzyme and enzyme regulator [goid 5524] [evidence IEA]; Interacting selectively with any nucleic acid [goid 3676] [evidence IEA]; Catalysis of the reaction: ATP + H2O = ADP + phosphate to drive the unwinding of a DNA or RNA helix [goid 8026] [evidence IEA] |
| Ddx4 | DEAD (Asp-Glu-Ala-Asp) box polypeptide 4 (Ddx4), mRNA. | 13206 | Catalysis of the hydrolysis of various bonds, e.g. C-O, C-N, C-C, phosphoric anhydride bonds, etc. Hydrolase is the systematic name for any enzyme of EC class 3 [goid 16787] [evidence IEA]; Catalysis of the reaction: NTP + H2O = NDP + phosphate to drive the unwinding of a DNA or RNA helix [goid 4386] [evidence IEA]; Interacting selectively with a nucleotide, any compound consisting of a nucleoside that is esterified with (ortho)phosphate or an oligophosphate at any hydroxyl group on the ribose or deoxyribose moiety [goid 166] [evidence IEA]; Interacting selectively with ATP, adenosine 5'-triphosphate, a universally important coenzyme and enzyme regulator [goid 5524] [evidence IEA]; Interacting selectively with any nucleic acid [goid 3676] [evidence IEA]; Catalysis of the reaction: ATP + H2O = ADP + phosphate to drive the unwinding of a DNA or RNA helix [goid 8026] [evidence IEA]; Interacting selectively with any protein or protein complex (a complex of two or more proteins that may include other nonprotein molecules) [goid 5515] [evidence IPI] |
| Dennd5b | DENN/MADD domain containing 5B (Dennd5b), mRNA. | 320560 |  |
| Dmrtc1a | DMRT-like family C1a (Dmrtc1a), transcript variant 1, mRNA. | 70887 |  |
| Dnaja2 | DnaJ (Hsp40) homolog, subfamily A, member 2 (Dnaja2), mRNA. | 56445 | Interacting selectively with any metal ion [goid 46872] [evidence IEA]; Interacting selectively with zinc (Zn) ions [goid 8270] [evidence IEA]; Interacting selectvely with a heat shock protein, any protein synthesized or activated in response to heat shock [goid 31072] [evidence IEA]; Interacting selectively with an unfolded protein [goid 51082] [evidence IEA] |
| Dnajc28 | DnaJ (Hsp40) homolog, subfamily C, member 28 (Dnajc28), transcript variant 2, mRNA. | 246738 | Interacting selectvely with a heat shock protein, any protein synthesized or activated in response to heat shock [goid 31072] [evidence IEA] |
| Dpp7 | dipeptidylpeptidase 7 (Dpp7), mRNA. | 83768 | Catalysis of the hydrolysis of N-terminal amino acid residues from in a polypeptide chain [goid 4177] [evidence IEA]; Catalysis of the hydrolysis of a peptide bond. A peptide bond is a covalent bond formed when the carbon atom from the carboxyl group of one amino acid shares electrons with the nitrogen atom from the amino group of a second amino acid [goid 8233] [evidence IEA]; Catalysis of the hydrolysis of various bonds, e.g. C-O, C-N, C-C, phosphoric anhydride bonds, etc. Hydrolase is the systematic name for any enzyme of EC class 3 [goid 16787] [evidence IEA]; Catalysis of the hydrolysis of internal, alpha-peptide bonds in a polypeptide chain by a catalytic mechanism that involves a catalytic triad consisting of a serine nucleophile that is activated by a proton relay involving an acidic residue (e.g. aspartate or glutamate) and a basic residue (usually histidine) [goid 4252] [evidence IEA]; Catalysis of the hydrolysis of peptide bonds in a polypeptide chain by a catalytic mechanism that involves a catalytic triad consisting of a serine nucleophile that is activated by a proton relay involving an acidic residue (e.g. aspartate or glutamate) and a basic residue (usually histidine) [goid 8236] [evidence IEA] |
| Dusp10 | dual specificity phosphatase 10 (Dusp10), mRNA. | 63953 | Catalysis of the hydrolysis of various bonds, e.g. C-O, C-N, C-C, phosphoric anhydride bonds, etc. Hydrolase is the systematic name for any enzyme of EC class 3 [goid 16787] [evidence IEA]; Catalysis of the reaction: a phosphoprotein + H2O = a protein + phosphate. Together with protein kinases, these enzymes control the state of phosphorylation of cell proteins and thereby provide an important mechanism for regulating cellular activity [goid 4721] [evidence IEA]; Catalysis of the hydrolysis of phosphoric monoesters, releasing inorganic phosphate [goid 16791] [evidence IEA]; Catalysis of the reaction: protein serine/threonine/tyrosine phosphate + H2O = protein serine/threonine/tyrosine + phosphate [goid 8138] [evidence IEA]; Catalysis of the reaction: protein tyrosine phosphate + H2O = protein tyrosine + phosphate [goid 4725] [evidence IEA]; Catalysis of the reaction: MAP kinase serine/threonine/tyrosine phosphate + H2O = MAP kinase serine/threonine/tyrosine + phosphate [goid 17017] [evidence IEA] |
| E030010A14Rik | RIKEN cDNA E030010A14 gene (E030010A14Rik), mRNA. | 226040 |  |
| EG245297 | predicted gene, EG245297 (EG245297), mRNA. | 245297 |  |
| EG328354 | predicted gene, EG328354 (EG328354), mRNA. | 328354 |  |
| EG328479 | predicted gene, EG328479 (EG328479), mRNA. | 328479 |  |
| EG381806 | predicted gene, EG381806 (EG381806) on chromosome 6. | 381806 |  |
| EG433224 | predicted gene, EG433224 (EG433224), non-coding RNA. | 433224 |  |
| EG665378 | predicted gene, EG665378 (EG665378), mRNA. | 665378 |  |
| EG668668 | predicted gene, EG668668 (EG668668), mRNA. | 668668 |  |
| Elf3 | E74-like factor 3 (Elf3), mRNA. | 13710 | Interacting selectively with DNA of a specific nucleotide composition, e.g. GC-rich DNA binding, or with a specific sequence motif or type of DNA e.g. promotor binding or rDNA binding [goid 43565] [evidence IEA]; The function of binding to a specific DNA sequence in order to modulate transcription. The transcription factor may or may not also interact selectively with a protein or macromolecular complex [goid 3700] [evidence IDA]; The function of binding to a specific DNA sequence in order to modulate transcription. The transcription factor may or may not also interact selectively with a protein or macromolecular complex [goid 3700] [evidence IMP]; Interacting selectively with DNA (deoxyribonucleic acid) [goid 3677] [evidence IDA] |
| Enpp7 | ectonucleotide pyrophosphatase/phosphodiesterase 7 (Enpp7), mRNA. | 238011 | Catalysis of the reaction: a nucleotide + H2O = a nucleoside + phosphate [goid 8252] [evidence ISO]; Catalysis of the reaction: sphingomyelin + H2O = N-acylsphingosine + choline phosphate [goid 4767] [evidence ISO] |
| Ermn | ermin, ERM-like protein (Ermn), mRNA. | 77767 | Interacting selectively with monomeric or multimeric forms of actin, including actin filaments [goid 3779] [evidence IEA]; Interacting selectively with an actin filament, also known as F-actin, a helical filamentous polymer of globular G-actin subunits [goid 51015] [evidence IDA]; Interacting selectively with any protein component of any cytoskeleton (actin, microtubule, or intermediate filament cytoskeleton) [goid 8092] [evidence IEA] |
| Evx1 | even skipped homeotic gene 1 homolog (Evx1), mRNA. | 14028 | Interacting selectively with DNA (deoxyribonucleic acid) [goid 3677] [evidence IEA]; Interacting selectively with DNA of a specific nucleotide composition, e.g. GC-rich DNA binding, or with a specific sequence motif or type of DNA e.g. promotor binding or rDNA binding [goid 43565] [evidence IEA]; The function of binding to a specific DNA sequence in order to modulate transcription. The transcription factor may or may not also interact selectively with a protein or macromolecular complex [goid 3700] [evidence IEA] |
| F8 | coagulation factor VIII (F8), mRNA. | 14069 | Interacting selectively with copper (Cu) ions [goid 5507] [evidence IEA]; Interacting selectively with calcium ions (Ca2+) [goid 5509] [evidence IEA]; Interacting selectively with any metal ion [goid 46872] [evidence IEA]; Catalysis of an oxidation-reduction (redox) reaction, a reversible chemical reaction in which the oxidation state of an atom or atoms within a molecule is altered. One substrate acts as a hydrogen or electron donor and becomes oxidized, while the other acts as hydrogen or electron acceptor and becomes reduced [goid 16491] [evidence IEA] |
| Fam119a | family with sequence similarity 119, member A (Fam119a), mRNA. | 67099 |  |
| Fam122b | family with sequence similarity 122, member B (Fam122b), mRNA. | 78755 |  |
| Fam134c | family with sequence similarity 134, member C (Fam134c), transcript variant 2, mRNA. | 67998 |  |
| Fastkd2 | FAST kinase domains 2 (Fastkd2), mRNA. | 75619 | Interacting selectively with ATP, adenosine 5'-triphosphate, a universally important coenzyme and enzyme regulator [goid 5524] [evidence IEA]; Catalysis of the phosphorylation of an amino acid residue in a protein, usually according to the reaction: a protein + ATP = a phosphoprotein + ADP [goid 4672] [evidence IEA] |
| Fbxl12 | F-box and leucine-rich repeat protein 12 (Fbxl12), transcript variant 1, mRNA. | 30843 | Elemental activities, such as catalysis or binding, describing the actions of a gene product at the molecular level. A given gene product may exhibit one or more molecular functions [goid 3674] [evidence ND ] |
| Fbxl5 | F-box and leucine-rich repeat protein 5 (Fbxl5), mRNA. | 242960 | Interacting selectively with any protein or protein complex (a complex of two or more proteins that may include other nonprotein molecules) [goid 5515] [evidence IEA]; Elemental activities, such as catalysis or binding, describing the actions of a gene product at the molecular level. A given gene product may exhibit one or more molecular functions [goid 3674] [evidence ND ] |
| Fbxl5 | F-box and leucine-rich repeat protein 5 (Fbxl5), mRNA. | 242960 | Interacting selectively with any protein or protein complex (a complex of two or more proteins that may include other nonprotein molecules) [goid 5515] [evidence IEA]; Elemental activities, such as catalysis or binding, describing the actions of a gene product at the molecular level. A given gene product may exhibit one or more molecular functions [goid 3674] [evidence ND ] |
| Frap1 | FK506 binding protein 12-rapamycin associated protein 1 (Frap1), mRNA. | 56717 | Catalysis of the transfer of a group, e.g. a methyl group, glycosyl group, acyl group, phosphorus-containing, or other groups, from one compound (generally regarded as the donor) to another compound (generally regarded as the acceptor). Transferase is the systematic name for any enzyme of EC class 2 [goid 16740] [evidence IEA]; Catalysis of the transfer of a phosphate group, usually from ATP, to a substrate molecule [goid 16301] [evidence IEA]; Catalysis of the reaction: ATP + a protein serine/threonine = ADP + protein serine/threonine phosphate [goid 4674] [evidence IDA]; Catalysis of the transfer of a phosphorus-containing group from one compound (donor) to an alcohol group (acceptor) [goid 16773] [evidence IEA]; Catalysis of the transfer of a phosphorus-containing group from one compound (donor) to another (acceptor) [goid 16772] [evidence IEA]; The selective, often stoichiometric, interaction of a molecule with one or more specific sites on another molecule [goid 5488] [evidence IEA]; Interacting selectively with any protein or protein complex (a complex of two or more proteins that may include other nonprotein molecules) [goid 5515] [evidence IPI] |
| Fzr1 | fizzy/cell division cycle 20 related 1 (Drosophila) (Fzr1), mRNA. | 56371 |  |
| GA_x5J8B7W2BV0-3116-4045 | olfactory receptor GA_x5J8B7W2BV0-3116-4045 (GA_x5J8B7W2BV0-3116-4045), mRNA. | 257663 |  |
| Galc | galactosylceramidase (Galc), mRNA. | 14420 | Catalysis of the hydrolysis of various bonds, e.g. C-O, C-N, C-C, phosphoric anhydride bonds, etc. Hydrolase is the systematic name for any enzyme of EC class 3 [goid 16787] [evidence IEA]; Catalysis of the hydrolysis of any glycosyl bond [goid 16798] [evidence IEA]; Catalysis of a biochemical reaction at physiological temperatures. In biologically catalyzed reactions, the reactants are known as substrates, and the catalysts are naturally occurring macromolecular substances known as enzymes. Enzymes possess specific binding sites for substrates, and are usually composed wholly or largely of protein, but RNA that has catalytic activity (ribozyme) is often also regarded as enzymatic [goid 3824] [evidence IEA]; Interacting selectively with cations, charged atoms or groups of atoms with a net positive charge [goid 43169] [evidence IEA]; Catalysis of the reaction: D-galactosyl-N-acylsphingosine + H2O = D-galactose + N-acylsphingosine [goid 4336] [evidence IMP] |
| Gbp2 | guanylate binding protein 2 (Gbp2), mRNA. | 14469 | Catalysis of the reaction: GTP + H2O = GDP + phosphate [goid 3924] [evidence ISS]; Interacting selectively with GTP, guanosine triphosphate [goid 5525] [evidence IEA]; Interacting selectively with a nucleotide, any compound consisting of a nucleoside that is esterified with (ortho)phosphate or an oligophosphate at any hydroxyl group on the ribose or deoxyribose moiety [goid 166] [evidence IEA] |
| Gca | grancalcin (Gca), mRNA. | 227960 | Interacting selectively with calcium ions (Ca2+) [goid 5509] [evidence IEA] |
| Gm129 | gene model 129, (NCBI) (Gm129), mRNA. XM_907670 XM_920513 XM_920520 XM_920527 XM_920533 | 229599 |  |
| Gm75 | gene model 75, (NCBI) (Gm75), mRNA. | 219026 |  |
| Gm817 | gene model 817, (NCBI) (Gm817), mRNA. | 329207 |  |
| Gnb2l1 | guanine nucleotide binding protein (G protein), beta polypeptide 2 like 1 (Gnb2l1), mRNA. | 14694 | Catalysis of the transfer of a phosphate group, usually from ATP, to a substrate molecule [goid 16301] [evidence IEA]; Combining with an extracellular or intracellular messenger to initiate a change in cell activity [goid 4872] [evidence ISA]; Interacting selectively with protein kinase C [goid 5080] [evidence ISA] |
| Gnb3 | guanine nucleotide binding protein (G protein), beta 3 (Gnb3), mRNA. | 14695 | Mediates the transfer of a signal from the outside to the inside of a cell by means other than the introduction of the signal molecule itself into the cell [goid 4871] [evidence IEA]; Catalysis of the reaction: GTP + H2O = GDP + phosphate [goid 3924] [evidence TAS] |
| Gper | G protein-coupled estrogen receptor 1 (Gper), mRNA. | 76854 | A receptor that binds an extracellular ligand and transmits the signal to a heterotrimeric G-protein complex. These receptors are characteristically seven-transmembrane receptors and are made up of hetero- or homodimers [goid 4930] [evidence IEA]; Combining with an extracellular or intracellular messenger to initiate a change in cell activity [goid 4872] [evidence IEA]; Mediates the transfer of a signal from the outside to the inside of a cell by means other than the introduction of the signal molecule itself into the cell [goid 4871] [evidence IEA]; Combining with a purine nucleotide and transmitting the signal to a heterotrimeric G-protein complex to initiate a change in cell activity [goid 45028] [evidence IEA] |
| Gpr1 | G protein-coupled receptor 1 (Gpr1), mRNA. | 241070 | A receptor that binds an extracellular ligand and transmits the signal to a heterotrimeric G-protein complex. These receptors are characteristically seven-transmembrane receptors and are made up of hetero- or homodimers [goid 4930] [evidence IEA]; Combining with an extracellular or intracellular messenger to initiate a change in cell activity [goid 4872] [evidence IEA]; Elemental activities, such as catalysis or binding, describing the actions of a gene product at the molecular level. A given gene product may exhibit one or more molecular functions [goid 3674] [evidence ND ]; Mediates the transfer of a signal from the outside to the inside of a cell by means other than the introduction of the signal molecule itself into the cell [goid 4871] [evidence IEA] |
| Gtf2a1 | general transcription factor II A, 1 (Gtf2a1), transcript variant 2, mRNA. | 83602 | Functions to initiate or regulate RNA polymerase II transcription [goid 3702] [evidence IEA] |
| Gtse1 | G two S phase expressed protein 1 (Gtse1), mRNA. | 29870 |  |
| Gvin1 | GTPase, very large interferon inducible 1 (Gvin1), transcript variant B, mRNA. | 74558 | Interacting selectively with GTP, guanosine triphosphate [goid 5525] [evidence IEA]; Interacting selectively with a nucleotide, any compound consisting of a nucleoside that is esterified with (ortho)phosphate or an oligophosphate at any hydroxyl group on the ribose or deoxyribose moiety [goid 166] [evidence IEA] |
| H2-Ab1 | histocompatibility 2, class II antigen A, beta 1 (H2-Ab1), mRNA. | 14961 | Interacting selectively with any protein or protein complex (a complex of two or more proteins that may include other nonprotein molecules) [goid 5515] [evidence IPI]; Interacting selectively with an antigen peptide, a fragment of a foreign protein derived by proteolysis within the cell [goid 42605] [evidence IDA] |
| Hectd2 | HECT domain containing 2 (Hectd2), mRNA. | 226098 | Catalysis of the ligation of two substances with concomitant breaking of a diphosphate linkage, usually in a nucleoside triphosphate. Ligase is the systematic name for any enzyme of EC class 6 [goid 16874] [evidence IEA]; Catalysis of the ligation of an acid to an amino acid via a carbon-nitrogen bond with concomitant breakage of a diphosphate linkage, usually in a nucleoside triphosphate [goid 16881] [evidence IEA]; Elemental activities, such as catalysis or binding, describing the actions of a gene product at the molecular level. A given gene product may exhibit one or more molecular functions [goid 3674] [evidence ND ] |
| Hectd2 | HECT domain containing 2 (Hectd2), mRNA. | 226098 | Catalysis of the ligation of two substances with concomitant breaking of a diphosphate linkage, usually in a nucleoside triphosphate. Ligase is the systematic name for any enzyme of EC class 6 [goid 16874] [evidence IEA]; Catalysis of the ligation of an acid to an amino acid via a carbon-nitrogen bond with concomitant breakage of a diphosphate linkage, usually in a nucleoside triphosphate [goid 16881] [evidence IEA]; Elemental activities, such as catalysis or binding, describing the actions of a gene product at the molecular level. A given gene product may exhibit one or more molecular functions [goid 3674] [evidence ND ] |
| Hif1an | hypoxia-inducible factor 1, alpha subunit inhibitor (Hif1an), mRNA. | 319594 | Catalysis of an oxidation-reduction (redox) reaction in which hydrogen or electrons are transferred from one donor, and two oxygen atoms is incorporated into a donor [goid 16702] [evidence IEA]; Interacting selectively with iron (Fe) ions [goid 5506] [evidence IEA]; Interacting selectively with any metal ion [goid 46872] [evidence IEA]; Catalysis of an oxidation-reduction (redox) reaction, a reversible chemical reaction in which the oxidation state of an atom or atoms within a molecule is altered. One substrate acts as a hydrogen or electron donor and becomes oxidized, while the other acts as hydrogen or electron acceptor and becomes reduced [goid 16491] [evidence IEA]; Catalysis of the reaction: peptide L-aspartate + 2-oxoglutarate + O2 = peptide 3-hydroxy-L-aspartate + succinate + CO2 [goid 4597] [evidence IEA] |
| Hif3a | hypoxia inducible factor 3, alpha subunit (Hif3a), mRNA. | 53417 | Interacting selectively with DNA (deoxyribonucleic acid) [goid 3677] [evidence IPI]; Plays a role in regulating transcription; may bind a promoter or enhancer DNA sequence or interact with a DNA-binding transcription factor [goid 30528] [evidence IEA]; Mediates the transfer of a signal from the outside to the inside of a cell by means other than the introduction of the signal molecule itself into the cell [goid 4871] [evidence IEA]; The function of binding to a specific DNA sequence in order to modulate transcription. The transcription factor may or may not also interact selectively with a protein or macromolecular complex [goid 3700] [evidence IPI] |
| Hn1 | hematological and neurological expressed sequence 1 (Hn1), mRNA. | 15374 | Elemental activities, such as catalysis or binding, describing the actions of a gene product at the molecular level. A given gene product may exhibit one or more molecular functions [goid 3674] [evidence ND ] |
| Hoxb6 | homeo box B6 (Hoxb6), mRNA. | 15414 | Interacting selectively with DNA (deoxyribonucleic acid) [goid 3677] [evidence IEA]; Interacting selectively with DNA of a specific nucleotide composition, e.g. GC-rich DNA binding, or with a specific sequence motif or type of DNA e.g. promotor binding or rDNA binding [goid 43565] [evidence IEA]; The function of binding to a specific DNA sequence in order to modulate transcription. The transcription factor may or may not also interact selectively with a protein or macromolecular complex [goid 3700] [evidence IEA] |
| Hsd3b2 | hydroxy-delta-5-steroid dehydrogenase, 3 beta- and steroid delta-isomerase 2 (Hsd3b2), mRNA. | 15493 | Catalysis of a biochemical reaction at physiological temperatures. In biologically catalyzed reactions, the reactants are known as substrates, and the catalysts are naturally occurring macromolecular substances known as enzymes. Enzymes possess specific binding sites for substrates, and are usually composed wholly or largely of protein, but RNA that has catalytic activity (ribozyme) is often also regarded as enzymatic [goid 3824] [evidence IEA]; Catalysis of an oxidation-reduction (redox) reaction, a reversible chemical reaction in which the oxidation state of an atom or atoms within a molecule is altered. One substrate acts as a hydrogen or electron donor and becomes oxidized, while the other acts as hydrogen or electron acceptor and becomes reduced [goid 16491] [evidence IEA]; Catalysis of the geometric or structural changes within one molecule. Isomerase is the systematic name for any enzyme of EC class 5 [goid 16853] [evidence IEA]; Catalysis of the reaction: 3-beta-hydroxy-delta(5)-steroid + NAD+ = 3-oxo-delta(5)-steroid + NADH + H+ [goid 3854] [evidence IEA]; The selective, often stoichiometric, interaction of a molecule with one or more specific sites on another molecule [goid 5488] [evidence IEA]; Catalysis of the reaction: a 3-oxo-delta(5)-steroid = a 3-oxo-delta(4)-steroid [goid 4769] [evidence IEA] |
| Hspe1 | heat shock protein 1 (chaperonin 10) (Hspe1), mRNA. | 15528 | Interacting selectively with ATP, adenosine 5'-triphosphate, a universally important coenzyme and enzyme regulator [goid 5524] [evidence IEA] |
| Iap |  |  |  |
| Idh2 | isocitrate dehydrogenase 2 (NADP+), mitochondrial (Idh2), nuclear gene encoding mitochondrial protein, mRNA. | 269951 | Interacting selectively with magnesium (Mg) ions [goid 287] [evidence IEA]; Interacting selectively with manganese (Mn) ions [goid 30145] [evidence IEA]; Catalysis of the reaction: isocitrate + NADP+ = 2-oxoglutarate + CO2 + NADPH + H+ [goid 4450] [evidence IDA]; Interacting selectively with any metal ion [goid 46872] [evidence IEA]; Catalysis of an oxidation-reduction (redox) reaction, a reversible chemical reaction in which the oxidation state of an atom or atoms within a molecule is altered. One substrate acts as a hydrogen or electron donor and becomes oxidized, while the other acts as hydrogen or electron acceptor and becomes reduced [goid 16491] [evidence IEA]; Catalysis of an oxidation-reduction (redox) reaction in which a CH-OH group acts as a hydrogen or electron donor and reduces NAD+ or NADP [goid 16616] [evidence IEA] |
| Ifngr2 | interferon gamma receptor 2 (Ifngr2), mRNA. | 15980 | Combining with an extracellular or intracellular messenger to initiate a change in cell activity [goid 4872] [evidence IEA] |
| Ift140 | intraflagellar transport 140 homolog (Chlamydomonas) (Ift140), mRNA. | 106633 |  |
| Ikzf1 | IKAROS family zinc finger 1 (Ikzf1), transcript variant 1, mRNA. | 22778 | Interacting selectively with any metal ion [goid 46872] [evidence IEA]; Interacting selectively with zinc (Zn) ions [goid 8270] [evidence IEA]; Interacting selectively with any nucleic acid [goid 3676] [evidence IEA]; Interacting selectively with any protein or protein complex (a complex of two or more proteins that may include other nonprotein molecules) [goid 5515] [evidence IPI]; The function of binding to a specific DNA sequence in order to modulate transcription. The transcription factor may or may not also interact selectively with a protein or macromolecular complex [goid 3700] [evidence IDA]; Interacting selectively with DNA (deoxyribonucleic acid) [goid 3677] [evidence IDA]; Any transcription regulator activity that prevents or downregulates transcription [goid 16564] [evidence IDA] |
| Il20ra | interleukin 20 receptor, alpha (Il20ra), mRNA. | 237313 | Combining with an extracellular or intracellular messenger to initiate a change in cell activity [goid 4872] [evidence IEA] |
| Ipp | IAP promoted placental gene (Ipp), mRNA. | 16351 | Interacting selectively with monomeric or multimeric forms of actin, including actin filaments [goid 3779] [evidence IEA]; Interacting selectively with any protein or protein complex (a complex of two or more proteins that may include other nonprotein molecules) [goid 5515] [evidence IEA] |
| Irgm1 | immunity-related GTPase family M member 1 (Irgm1), mRNA. | 15944 | Catalysis of the hydrolysis of various bonds, e.g. C-O, C-N, C-C, phosphoric anhydride bonds, etc. Hydrolase is the systematic name for any enzyme of EC class 3 [goid 16787] [evidence IEA]; Interacting selectively with a nucleotide, any compound consisting of a nucleoside that is esterified with (ortho)phosphate or an oligophosphate at any hydroxyl group on the ribose or deoxyribose moiety [goid 166] [evidence IEA]; Interacting selectively with GTP, guanosine triphosphate [goid 5525] [evidence IEA] |
| Itga7 | integrin alpha 7 (Itga7), mRNA. | 16404 | Interacting selectively with calcium ions (Ca2+) [goid 5509] [evidence IEA]; Interacting selectively with any protein or protein complex (a complex of two or more proteins that may include other nonprotein molecules) [goid 5515] [evidence IEA]; Combining with an extracellular or intracellular messenger to initiate a change in cell activity [goid 4872] [evidence IEA] |
| Itgb2 | integrin beta 2 (Itgb2), mRNA. | 16414 | Combining with an extracellular or intracellular messenger to initiate a change in cell activity [goid 4872] [evidence IEA]; Interacting selectively with any protein or protein complex (a complex of two or more proteins that may include other nonprotein molecules) [goid 5515] [evidence IEA]; The selective, often stoichiometric, interaction of a molecule with one or more specific sites on another molecule [goid 5488] [evidence IEA] |
| Kcna10 | potassium voltage-gated channel, shaker-related subfamily, member 10 (Kcna10), mRNA. | 242151 | Catalysis of the transmembrane transfer of a potassium ion by a voltage-gated channel [goid 5249] [evidence ISO] |
| Kcnh2 | potassium voltage-gated channel, subfamily H (eag-related), member 2 (Kcnh2), mRNA. | 16511 | Catalysis of facilitated diffusion of an ion (by an energy-independent process) by passage through a transmembrane aqueous pore or channel without evidence for a carrier-mediated mechanism [goid 5216] [evidence IEA]; Catalysis of facilitated diffusion of a potassium ion (by an energy-independent process) involving passage through a transmembrane aqueous pore or channel without evidence for a carrier-mediated mechanism [goid 5267] [evidence IEA]; Catalysis of the transmembrane transfer of an ion by a voltage-gated channel. An ion is an atom or group of atoms carrying an electric charge by virtue of having gained or lost one or more electrons [goid 5244] [evidence IEA]; Catalysis of the phosphorylation of a specific transcription regulator in response to the presence of a particular signal substance outside the cell [goid 155] [evidence IEA]; Mediates the transfer of a signal from the outside to the inside of a cell by means other than the introduction of the signal molecule itself into the cell [goid 4871] [evidence IEA]; Interacting selectively with potassium (K+) ions [goid 30955] [evidence IEA]; Catalysis of the transmembrane transfer of a potassium ion by a voltage-gated channel [goid 5249] [evidence IEA] |
| Kcnh6 | potassium voltage-gated channel, subfamily H (eag-related), member 6 (Kcnh6), mRNA. XM_922264 XM_922267 | 192775 | Catalysis of facilitated diffusion of an ion (by an energy-independent process) by passage through a transmembrane aqueous pore or channel without evidence for a carrier-mediated mechanism [goid 5216] [evidence IEA] |
| Kcnmb1 | potassium large conductance calcium-activated channel, subfamily M, beta member 1 (Kcnmb1), mRNA. | 16533 | Catalysis of facilitated diffusion of an ion (by an energy-independent process) by passage through a transmembrane aqueous pore or channel without evidence for a carrier-mediated mechanism [goid 5216] [evidence IEA]; Catalysis of the calcium concentration-regulatable energy-independent passage of potassium ions across a lipid bilayer down a concentration gradient [goid 15269] [evidence IGI] |
| Klhdc4 | kelch domain containing 4 (Klhdc4), mRNA. | 234825 |  |
| Krtap8-2 | keratin associated protein 8-2 (Krtap8-2), mRNA. | 16704 |  |
| Lep | leptin (Lep), mRNA. | 16846 | The action characteristic of a hormone, any substance formed in very small amounts in one specialized organ or group of cells and carried (sometimes in the bloodstream) to another organ or group of cells in the same organism, upon which it has a specific regulatory action. The term was originally applied to agents with a stimulatory physiological action in vertebrate animals (as opposed to a chalone, which has a depressant action). Usage is now extended to regulatory compounds in lower animals and plants, and to synthetic substances having comparable effects [goid 5179] [evidence IEA]; Interacting selectively with any protein or protein complex (a complex of two or more proteins that may include other nonprotein molecules) [goid 5515] [evidence IPI]; The function that stimulates a cell to grow or proliferate. Most growth factors have other actions besides the induction of cell growth or proliferation [goid 8083] [evidence IDA] |
| Lincr | lung-inducible neuralized-related C3HC4 RING domain protein (Lincr), mRNA. | 214854 | Catalysis of the ligation of two substances with concomitant breaking of a diphosphate linkage, usually in a nucleoside triphosphate. Ligase is the systematic name for any enzyme of EC class 6 [goid 16874] [evidence IEA]; Elemental activities, such as catalysis or binding, describing the actions of a gene product at the molecular level. A given gene product may exhibit one or more molecular functions [goid 3674] [evidence ND ]; Interacting selectively with any protein or protein complex (a complex of two or more proteins that may include other nonprotein molecules) [goid 5515] [evidence IEA]; Interacting selectively with any metal ion [goid 46872] [evidence IEA]; Interacting selectively with zinc (Zn) ions [goid 8270] [evidence IEA] |
| LOC100044160 | PREDICTED: hypothetical protein LOC100044160 (LOC100044160), mRNA. | 100044160 |  |
| LOC100045019 | PREDICTED: similar to Tubulin, gamma 2 (LOC100045019), mRNA. | 100045019 |  |
| LOC100045343 | PREDICTED: similar to CDNA sequence BC046404 (LOC100045343), misc RNA. | 100045343 |  |
| LOC100046609 | PREDICTED: similar to RIKEN cDNA 4932415G16 gene (LOC100046609), misc RNA. | 100046609 |  |
| LOC100047963 | PREDICTED: similar to ADIR1 (LOC100047963), mRNA. | 100047963 |  |
| LOC100048346 | PREDICTED: similar to ubiquitin specific protease UBP43 (LOC100048346), mRNA. | 100048346 |  |
| LOC100048384 | PREDICTED: similar to programmed cell death protein 7 (LOC100048384), misc RNA. | 100048384 |  |
| Lrch4 | leucine-rich repeats and calponin homology (CH) domain containing 4 (Lrch4), mRNA. | 231798 | Interacting selectively with any protein or protein complex (a complex of two or more proteins that may include other nonprotein molecules) [goid 5515] [evidence IPI]; Any transcription regulator activity that prevents or downregulates transcription [goid 16564] [evidence IMP] |
| Lrrc39 | leucine rich repeat containing 39 (Lrrc39), transcript variant 1, mRNA. | 109245 | Interacting selectively with any protein or protein complex (a complex of two or more proteins that may include other nonprotein molecules) [goid 5515] [evidence IEA] |
| Luzp2 | leucine zipper protein 2 (Luzp2), mRNA. | 233271 | Elemental activities, such as catalysis or binding, describing the actions of a gene product at the molecular level. A given gene product may exhibit one or more molecular functions [goid 3674] [evidence ND ] |
| Magi1 | membrane associated guanylate kinase, WW and PDZ domain containing 1 (Magi1), transcript variant 1, mRNA. | 14924 | Catalysis of the transfer of a phosphate group, usually from ATP, to a substrate molecule [goid 16301] [evidence IEA]; Interacting selectively with a nucleotide, any compound consisting of a nucleoside that is esterified with (ortho)phosphate or an oligophosphate at any hydroxyl group on the ribose or deoxyribose moiety [goid 166] [evidence IEA]; Interacting selectively with any protein or protein complex (a complex of two or more proteins that may include other nonprotein molecules) [goid 5515] [evidence IPI]; Interacting selectively with ATP, adenosine 5'-triphosphate, a universally important coenzyme and enzyme regulator [goid 5524] [evidence IEA] |
| Mapk1 | mitogen-activated protein kinase 1 (Mapk1), transcript variant 1, mRNA. | 26413 | Interacting selectively with a phosphorylated tyrosine residue within a protein [goid 1784] [evidence IMP]; Interacting selectively with a nucleotide, any compound consisting of a nucleoside that is esterified with (ortho)phosphate or an oligophosphate at any hydroxyl group on the ribose or deoxyribose moiety [goid 166] [evidence IEA]; Catalysis of the transfer of a phosphate group, usually from ATP, to a substrate molecule [goid 16301] [evidence IDA]; Catalysis of the transfer of a group, e.g. a methyl group, glycosyl group, acyl group, phosphorus-containing, or other groups, from one compound (generally regarded as the donor) to another compound (generally regarded as the acceptor). Transferase is the systematic name for any enzyme of EC class 2 [goid 16740] [evidence IEA]; Interacting selectively with ATP, adenosine 5'-triphosphate, a universally important coenzyme and enzyme regulator [goid 5524] [evidence IEA]; Catalysis of the phosphorylation of an amino acid residue in a protein, usually according to the reaction: a protein + ATP = a phosphoprotein + ADP [goid 4672] [evidence IDA]; Catalysis of the reaction: ATP + a protein serine/threonine = ADP + protein serine/threonine phosphate [goid 4674] [evidence ISO]; Catalysis of the phosphorylation of proteins. Mitogen-activated protein kinase; a family of protein kinases that perform a crucial step in relaying signals from the plasma membrane to the nucleus. They are activated by a wide range of proliferation- or differentiation-inducing signals; activation is strong with agonists such as polypeptide growth factors and tumor-promoting phorbol esters, but weak (in most cell backgrounds) by stress stimuli [goid 4707] [evidence IDA]; Catalysis of the phosphorylation of proteins. Mitogen-activated protein kinase; a family of protein kinases that perform a crucial step in relaying signals from the plasma membrane to the nucleus. They are activated by a wide range of proliferation- or differentiation-inducing signals; activation is strong with agonists such as polypeptide growth factors and tumor-promoting phorbol esters, but weak (in most cell backgrounds) by stress stimuli [goid 4707] [evidence IMP]; Catalysis of the phosphorylation of proteins. Mitogen-activated protein kinase; a family of protein kinases that perform a crucial step in relaying signals from the plasma membrane to the nucleus. They are activated by a wide range of proliferation- or differentiation-inducing signals; activation is strong with agonists such as polypeptide growth factors and tumor-promoting phorbol esters, but weak (in most cell backgrounds) by stress stimuli [goid 4707] [evidence IDA]; Interacting selectively with any protein or protein complex (a complex of two or more proteins that may include other nonprotein molecules) [goid 5515] [evidence IPI]; [goid 16908] [evidence IDA] |
| Mapk13 | mitogen-activated protein kinase 13 (Mapk13), mRNA. | 26415 | Interacting selectively with ATP, adenosine 5'-triphosphate, a universally important coenzyme and enzyme regulator [goid 5524] [evidence IEA]; Catalysis of the reaction: ATP + a protein serine/threonine = ADP + protein serine/threonine phosphate [goid 4674] [evidence ISO]; Catalysis of the phosphorylation of proteins. Mitogen-activated protein kinase; a family of protein kinases that perform a crucial step in relaying signals from the plasma membrane to the nucleus. They are activated by a wide range of proliferation- or differentiation-inducing signals; activation is strong with agonists such as polypeptide growth factors and tumor-promoting phorbol esters, but weak (in most cell backgrounds) by stress stimuli [goid 4707] [evidence IEA]; Catalysis of the transfer of a phosphate group, usually from ATP, to a substrate molecule [goid 16301] [evidence IEA]; Interacting selectively with a nucleotide, any compound consisting of a nucleoside that is esterified with (ortho)phosphate or an oligophosphate at any hydroxyl group on the ribose or deoxyribose moiety [goid 166] [evidence IEA]; Catalysis of the transfer of a group, e.g. a methyl group, glycosyl group, acyl group, phosphorus-containing, or other groups, from one compound (generally regarded as the donor) to another compound (generally regarded as the acceptor). Transferase is the systematic name for any enzyme of EC class 2 [goid 16740] [evidence IEA]; Catalysis of the phosphorylation of an amino acid residue in a protein, usually according to the reaction: a protein + ATP = a phosphoprotein + ADP [goid 4672] [evidence IEA]; Interacting selectively with any protein or protein complex (a complex of two or more proteins that may include other nonprotein molecules) [goid 5515] [evidence ISO] |
| Marveld3 | MARVEL (membrane-associating) domain containing 3 (Marveld3), transcript variant 1, mRNA. | 73608 |  |
| Mbd3l2 | methyl-CpG binding domain protein 3-like 2 (Mbd3l2), mRNA. | 234988 |  |
| Mcfd2 | multiple coagulation factor deficiency 2 (Mcfd2), mRNA. | 193813 | Interacting selectively with calcium ions (Ca2+) [goid 5509] [evidence IEA]; Elemental activities, such as catalysis or binding, describing the actions of a gene product at the molecular level. A given gene product may exhibit one or more molecular functions [goid 3674] [evidence ND ] |
| Mdfic | MyoD family inhibitor domain containing (Mdfic), mRNA. | 16543 |  |
| Mgst2 | microsomal glutathione S-transferase 2 (Mgst2), mRNA. | 211666 | Catalysis of the transfer of a group, e.g. a methyl group, glycosyl group, acyl group, phosphorus-containing, or other groups, from one compound (generally regarded as the donor) to another compound (generally regarded as the acceptor). Transferase is the systematic name for any enzyme of EC class 2 [goid 16740] [evidence IEA] |
| Mid1 |  |  | Catalysis of the ligation of two substances with concomitant breaking of a diphosphate linkage, usually in a nucleoside triphosphate. Ligase is the systematic name for any enzyme of EC class 6 [goid 16874] [evidence IEA]; Interacting selectively with zinc (Zn) ions [goid 8270] [evidence IEA]; Interacting selectively with any metal ion [goid 46872] [evidence IEA]; Interacting selectively with any protein or protein complex (a complex of two or more proteins that may include other nonprotein molecules) [goid 5515] [evidence IPI] |
| Mrps14 | mitochondrial ribosomal protein S14 (Mrps14), nuclear gene encoding mitochondrial protein, mRNA. | 64659 | The action of a molecule that contributes to the structural integrity of the ribosome [goid 3735] [evidence ISS] |
| Mycbp2 | MYC binding protein 2 (Mycbp2), mRNA. | 105689 | Interacting selectively with zinc (Zn) ions [goid 8270] [evidence IEA]; Interacting selectively with any metal ion [goid 46872] [evidence IEA]; Interacting selectively with an identical protein to form a homodimer [goid 42803] [evidence IPI]; Catalysis of the ligation of two substances with concomitant breaking of a diphosphate linkage, usually in a nucleoside triphosphate. Ligase is the systematic name for any enzyme of EC class 6 [goid 16874] [evidence IEA]; Interacting selectively with any protein or protein complex (a complex of two or more proteins that may include other nonprotein molecules) [goid 5515] [evidence IPI] |
| Mypn | PREDICTED: myopalladin (Mypn), mRNA. | 68802 | Interacting selectively with monomeric or multimeric forms of actin, including actin filaments [goid 3779] [evidence IEA] |
| Myt1l | myelin transcription factor 1-like (Myt1l), transcript variant 2, mRNA. | 17933 | Interacting selectively with zinc (Zn) ions [goid 8270] [evidence IEA]; Interacting selectively with DNA (deoxyribonucleic acid) [goid 3677] [evidence IEA]; Interacting selectively with any metal ion [goid 46872] [evidence IEA]; The function of binding to a specific DNA sequence in order to modulate transcription. The transcription factor may or may not also interact selectively with a protein or macromolecular complex [goid 3700] [evidence IEA] |
| Nags | N-acetylglutamate synthase (Nags), transcript variant 1, mRNA. | 217214 | Catalysis of the transfer of a group, e.g. a methyl group, glycosyl group, acyl group, phosphorus-containing, or other groups, from one compound (generally regarded as the donor) to another compound (generally regarded as the acceptor). Transferase is the systematic name for any enzyme of EC class 2 [goid 16740] [evidence IEA]; Catalysis of the generalized reaction: acyl-carrier + reactant = acyl-reactant + carrier [goid 8415] [evidence IEA]; Catalysis of the reaction: ATP + N-acetyl-L-glutamate = ADP + N-acetyl-L-glutamate 5-phosphate [goid 3991] [evidence IEA]; Catalysis of the reaction: acetyl-CoA + L-glutamate = CoA + N-acetyl-L-glutamate [goid 4042] [evidence IDA]; Catalysis of the transfer of an acetyl group to a nitrogen atom on the acceptor molecule [goid 8080] [evidence IEA] |
| Nampt | nicotinamide phosphoribosyltransferase (Nampt), mRNA. | 59027 | Catalysis of the transfer of a group, e.g. a methyl group, glycosyl group, acyl group, phosphorus-containing, or other groups, from one compound (generally regarded as the donor) to another compound (generally regarded as the acceptor). Transferase is the systematic name for any enzyme of EC class 2 [goid 16740] [evidence IEA]; Catalysis of the transfer of a glycosyl group from one compound (donor) to another (acceptor) [goid 16757] [evidence IEA]; Catalysis of the reaction: pyrophosphate + nicotinamide nucleotide = PRPP + niacinamide [goid 47280] [evidence IDA] |
| Nap1l3 | nucleosome assembly protein 1-like 3 (Nap1l3), mRNA. | 54561 |  |
| Ndor1 | NADPH dependent diflavin oxidoreductase 1 (Ndor1), mRNA. | 78797 | Catalysis of an oxidation-reduction (redox) reaction, a reversible chemical reaction in which the oxidation state of an atom or atoms within a molecule is altered. One substrate acts as a hydrogen or electron donor and becomes oxidized, while the other acts as hydrogen or electron acceptor and becomes reduced [goid 16491] [evidence IEA]; Interacting selectively with iron (Fe) ions [goid 5506] [evidence IEA]; Interacting selectively with FMN, flavin mononucleotide, the coenzyme or the prosthetic group of various flavoprotein oxidoreductase enzymes [goid 10181] [evidence IEA]; Any molecular entity that serves as an electron acceptor and electron donor in an electron transport system [goid 9055] [evidence IEA] |
| Net1 | neuroepithelial cell transforming gene 1 (Net1), transcript variant 2, mRNA. | 56349 | Stimulates the exchange of guanyl nucleotides by a GTPase. Under normal cellular physiological conditions, the concentration of GTP is higher than that of GDP, favoring the replacement of GDP by GTP in association with the GTPase [goid 5085] [evidence IEA]; Stimulates the exchange of guanyl nucleotides by a GTPase of the Rho family. Under normal cellular physiological conditions, the concentration of GTP is higher than that of GDP, favoring the replacement of GDP by GTP in association with the GTPase [goid 5089] [evidence IEA] |
| Neu2 | neuraminidase 2 (Neu2), mRNA. | 23956 | Catalysis of the hydrolysis of various bonds, e.g. C-O, C-N, C-C, phosphoric anhydride bonds, etc. Hydrolase is the systematic name for any enzyme of EC class 3 [goid 16787] [evidence IEA]; Catalysis of the hydrolysis of any glycosyl bond [goid 16798] [evidence IEA]; Catalysis of the hydrolysis of alpha-(2->3)-, alpha-(2->6)-, alpha-(2->8)-glycosidic linkages of terminal sialic residues in oligosaccharides, glycoproteins, glycolipids, colominic acid and synthetic substrates [goid 4308] [evidence IEA] |
| Neurod1 | neurogenic differentiation 1 (Neurod1), mRNA. | 18012 | Plays a role in regulating transcription; may bind a promoter or enhancer DNA sequence or interact with a DNA-binding transcription factor [goid 30528] [evidence IEA]; Interacting selectively with any protein or protein complex (a complex of two or more proteins that may include other nonprotein molecules) [goid 5515] [evidence IPI]; Interacting selectively with DNA (deoxyribonucleic acid) [goid 3677] [evidence IDA]; Any transcription regulator activity required for initiation or upregulation of transcription [goid 16563] [evidence IDA]; Interacting selectively with a nonidentical protein to form a heterodimer [goid 46982] [evidence IPI]; Interacting selectively with DNA of a specific nucleotide composition, e.g. GC-rich DNA binding, or with a specific sequence motif or type of DNA e.g. promotor binding or rDNA binding [goid 43565] [evidence IDA] |
| Nkain3 | Na+/K+ transporting ATPase interacting 3 (Nkain3), mRNA. | 269513 |  |
| Nlrp2 | NLR family, pyrin domain containing 2 (Nlrp2), mRNA. | 232827 | Interacting selectively with ATP, adenosine 5'-triphosphate, a universally important coenzyme and enzyme regulator [goid 5524] [evidence IEA]; Interacting selectively with a nucleotide, any compound consisting of a nucleoside that is esterified with (ortho)phosphate or an oligophosphate at any hydroxyl group on the ribose or deoxyribose moiety [goid 166] [evidence IEA] |
| Npy2r | neuropeptide Y receptor Y2 (Npy2r), mRNA. | 18167 | A receptor that binds an extracellular ligand and transmits the signal to a heterotrimeric G-protein complex. These receptors are characteristically seven-transmembrane receptors and are made up of hetero- or homodimers [goid 4930] [evidence IEA]; Combining with an extracellular or intracellular messenger to initiate a change in cell activity [goid 4872] [evidence IEA]; Mediates the transfer of a signal from the outside to the inside of a cell by means other than the introduction of the signal molecule itself into the cell [goid 4871] [evidence IEA]; Combining with neuropeptide Y to initiate a change in cell activity [goid 4983] [evidence IDA]; Combining with gut peptide YY to initiate a change in cell activity [goid 1601] [evidence IDA] |
| Nr0b1 | nuclear receptor subfamily 0, group B, member 1 (Nr0b1), mRNA. | 11614 | Combining with an extracellular or intracellular messenger to initiate a change in cell activity [goid 4872] [evidence IEA]; Interacting selectively with an RNA molecule or a portion thereof [goid 3723] [evidence IDA]; The function of binding to a specific DNA sequence in order to modulate transcription. The transcription factor may or may not also interact selectively with a protein or macromolecular complex [goid 3700] [evidence IEA]; Combining with a steroid hormone to initiate a change in cell activity [goid 3707] [evidence IEA]; Interacting selectively with DNA (deoxyribonucleic acid) [goid 3677] [evidence IEA]; Any transcription regulator activity that prevents or downregulates transcription [goid 16564] [evidence IDA]; A ligand-dependent receptor found in the nucleus of the cell [goid 4879] [evidence IEA]; The function of a transcription cofactor that represses transcription from a RNA polymerase II promoter; does not bind DNA itself [goid 3714] [evidence IGI]; Interacting selectively with any protein or protein complex (a complex of two or more proteins that may include other nonprotein molecules) [goid 5515] [evidence IPI] |
| Nt5dc3 | 5'-nucleotidase domain containing 3 (Nt5dc3), transcript variant 2, mRNA. | 103220 | Catalysis of the hydrolysis of various bonds, e.g. C-O, C-N, C-C, phosphoric anhydride bonds, etc. Hydrolase is the systematic name for any enzyme of EC class 3 [goid 16787] [evidence IEA]; Interacting selectively with magnesium (Mg) ions [goid 287] [evidence IEA]; Interacting selectively with any metal ion [goid 46872] [evidence IEA] |
| Nutf2 | nuclear transport factor 2 (Nutf2), mRNA. | 68051 | Enables the directed movement of proteins into, out of, within or between cells [goid 8565] [evidence IDA] |
| Obfc1 | oligonucleotide/oligosaccharide-binding fold containing 1 (Obfc1), mRNA. | 108689 | Interacting selectively with any nucleic acid [goid 3676] [evidence IEA] |
| Olfm2 | olfactomedin 2 (Olfm2), mRNA. | 244723 |  |
| Olfr1154 | olfactory receptor 1154 (Olfr1154), mRNA. | 258641 | A receptor that binds an extracellular ligand and transmits the signal to a heterotrimeric G-protein complex. These receptors are characteristically seven-transmembrane receptors and are made up of hetero- or homodimers [goid 4930] [evidence IEA]; Combining with airborne compounds to initiate a change in cell activity. These receptors are used for the sense of smell [goid 4984] [evidence ISA]; Combining with an extracellular or intracellular messenger to initiate a change in cell activity [goid 4872] [evidence IEA]; Mediates the transfer of a signal from the outside to the inside of a cell by means other than the introduction of the signal molecule itself into the cell [goid 4871] [evidence IEA] |
| Olfr174 | olfactory receptor 174 (Olfr174), mRNA. | 259004 | A receptor that binds an extracellular ligand and transmits the signal to a heterotrimeric G-protein complex. These receptors are characteristically seven-transmembrane receptors and are made up of hetero- or homodimers [goid 4930] [evidence IEA]; Combining with an extracellular or intracellular messenger to initiate a change in cell activity [goid 4872] [evidence IEA]; Combining with airborne compounds to initiate a change in cell activity. These receptors are used for the sense of smell [goid 4984] [evidence ISA]; Mediates the transfer of a signal from the outside to the inside of a cell by means other than the introduction of the signal molecule itself into the cell [goid 4871] [evidence IEA] |
| Olfr272 | olfactory receptor 272 (Olfr272), mRNA. | 258836 | A receptor that binds an extracellular ligand and transmits the signal to a heterotrimeric G-protein complex. These receptors are characteristically seven-transmembrane receptors and are made up of hetero- or homodimers [goid 4930] [evidence IEA]; Combining with an extracellular or intracellular messenger to initiate a change in cell activity [goid 4872] [evidence IEA]; Mediates the transfer of a signal from the outside to the inside of a cell by means other than the introduction of the signal molecule itself into the cell [goid 4871] [evidence IEA]; Combining with airborne compounds to initiate a change in cell activity. These receptors are used for the sense of smell [goid 4984] [evidence ISA] |
| Olfr62 | olfactory receptor 62 (Olfr62), mRNA. | 18363 | A receptor that binds an extracellular ligand and transmits the signal to a heterotrimeric G-protein complex. These receptors are characteristically seven-transmembrane receptors and are made up of hetero- or homodimers [goid 4930] [evidence IEA]; Combining with an extracellular or intracellular messenger to initiate a change in cell activity [goid 4872] [evidence IEA]; Mediates the transfer of a signal from the outside to the inside of a cell by means other than the introduction of the signal molecule itself into the cell [goid 4871] [evidence IEA]; Combining with airborne compounds to initiate a change in cell activity. These receptors are used for the sense of smell [goid 4984] [evidence ISA] |
| Olfr770 | olfactory receptor 770 (Olfr770), mRNA. | 258862 | A receptor that binds an extracellular ligand and transmits the signal to a heterotrimeric G-protein complex. These receptors are characteristically seven-transmembrane receptors and are made up of hetero- or homodimers [goid 4930] [evidence IEA]; Combining with airborne compounds to initiate a change in cell activity. These receptors are used for the sense of smell [goid 4984] [evidence ISA]; Combining with an extracellular or intracellular messenger to initiate a change in cell activity [goid 4872] [evidence IEA]; Mediates the transfer of a signal from the outside to the inside of a cell by means other than the introduction of the signal molecule itself into the cell [goid 4871] [evidence IEA] |
| Olfr826 | olfactory receptor 826 (Olfr826), mRNA. | 258671 | A receptor that binds an extracellular ligand and transmits the signal to a heterotrimeric G-protein complex. These receptors are characteristically seven-transmembrane receptors and are made up of hetero- or homodimers [goid 4930] [evidence IEA]; Mediates the transfer of a signal from the outside to the inside of a cell by means other than the introduction of the signal molecule itself into the cell [goid 4871] [evidence IEA]; Combining with an extracellular or intracellular messenger to initiate a change in cell activity [goid 4872] [evidence IEA]; Combining with airborne compounds to initiate a change in cell activity. These receptors are used for the sense of smell [goid 4984] [evidence ISA] |
| Olfr871 | olfactory receptor 871 (Olfr871), mRNA. | 258905 | Combining with airborne compounds to initiate a change in cell activity. These receptors are used for the sense of smell [goid 4984] [evidence ISA]; A receptor that binds an extracellular ligand and transmits the signal to a heterotrimeric G-protein complex. These receptors are characteristically seven-transmembrane receptors and are made up of hetero- or homodimers [goid 4930] [evidence IEA]; Mediates the transfer of a signal from the outside to the inside of a cell by means other than the introduction of the signal molecule itself into the cell [goid 4871] [evidence IEA]; Combining with an extracellular or intracellular messenger to initiate a change in cell activity [goid 4872] [evidence IEA] |
| Olfr881 | olfactory receptor 881 (Olfr881), mRNA. | 258413 | A receptor that binds an extracellular ligand and transmits the signal to a heterotrimeric G-protein complex. These receptors are characteristically seven-transmembrane receptors and are made up of hetero- or homodimers [goid 4930] [evidence IEA]; Combining with an extracellular or intracellular messenger to initiate a change in cell activity [goid 4872] [evidence IEA]; Mediates the transfer of a signal from the outside to the inside of a cell by means other than the introduction of the signal molecule itself into the cell [goid 4871] [evidence IEA]; Combining with airborne compounds to initiate a change in cell activity. These receptors are used for the sense of smell [goid 4984] [evidence ISA] |
| Olfr884 | olfactory receptor 884 (Olfr884), mRNA. | 257996 | A receptor that binds an extracellular ligand and transmits the signal to a heterotrimeric G-protein complex. These receptors are characteristically seven-transmembrane receptors and are made up of hetero- or homodimers [goid 4930] [evidence IEA]; Combining with an extracellular or intracellular messenger to initiate a change in cell activity [goid 4872] [evidence IEA]; Mediates the transfer of a signal from the outside to the inside of a cell by means other than the introduction of the signal molecule itself into the cell [goid 4871] [evidence IEA]; Combining with airborne compounds to initiate a change in cell activity. These receptors are used for the sense of smell [goid 4984] [evidence ISA] |
| Olfr910 | olfactory receptor 910 (Olfr910), mRNA. | 258807 | A receptor that binds an extracellular ligand and transmits the signal to a heterotrimeric G-protein complex. These receptors are characteristically seven-transmembrane receptors and are made up of hetero- or homodimers [goid 4930] [evidence IEA]; Mediates the transfer of a signal from the outside to the inside of a cell by means other than the introduction of the signal molecule itself into the cell [goid 4871] [evidence IEA]; Combining with an extracellular or intracellular messenger to initiate a change in cell activity [goid 4872] [evidence IEA]; Combining with airborne compounds to initiate a change in cell activity. These receptors are used for the sense of smell [goid 4984] [evidence ISA] |
| Olfr985 | olfactory receptor 985 (Olfr985), mRNA. | 258854 | A receptor that binds an extracellular ligand and transmits the signal to a heterotrimeric G-protein complex. These receptors are characteristically seven-transmembrane receptors and are made up of hetero- or homodimers [goid 4930] [evidence IEA]; Combining with airborne compounds to initiate a change in cell activity. These receptors are used for the sense of smell [goid 4984] [evidence ISA]; Combining with an extracellular or intracellular messenger to initiate a change in cell activity [goid 4872] [evidence IEA]; Mediates the transfer of a signal from the outside to the inside of a cell by means other than the introduction of the signal molecule itself into the cell [goid 4871] [evidence IEA] |
| Omt2b | oocyte maturation, beta (Omt2b), mRNA. | 382088 |  |
| Orai2 | ORAI calcium release-activated calcium modulator 2 (Orai2), mRNA. | 269717 |  |
| ORF9 | open reading frame 9 (ORF9), mRNA. | 52793 | Functions to control the survival, growth, differentiation and effector function of tissues and cells [goid 5125] [evidence NAS] |
| Osgepl1 | O-sialoglycoprotein endopeptidase-like 1 (Osgepl1), mRNA. | 72085 | Catalysis of the hydrolysis of various bonds, e.g. C-O, C-N, C-C, phosphoric anhydride bonds, etc. Hydrolase is the systematic name for any enzyme of EC class 3 [goid 16787] [evidence IEA]; Catalysis of the hydrolysis of a peptide bond. A peptide bond is a covalent bond formed when the carbon atom from the carboxyl group of one amino acid shares electrons with the nitrogen atom from the amino group of a second amino acid [goid 8233] [evidence IEA]; Interacting selectively with any metal ion [goid 46872] [evidence IEA]; Interacting selectively with zinc (Zn) ions [goid 8270] [evidence IEA]; Catalysis of the hydrolysis of peptide bonds by a mechanism in which water acts as a nucleophile, one or two metal ions hold the water molecule in place, and charged amino acid side chains are ligands for the metal ions [goid 8237] [evidence IEA]; Catalysis of the hydrolysis of internal, alpha-peptide bonds in a polypeptide chain [goid 4175] [evidence IEA]; Catalysis of the hydrolysis of internal, alpha-peptide bonds in a polypeptide chain by a mechanism in which water acts as a nucleophile, one or two metal ions hold the water molecule in place, and charged amino acid side chains are ligands for the metal ions [goid 4222] [evidence IEA] |
| P140 | P140 gene (P140), mRNA. | 56013 | Interacting selectively with any protein or protein complex (a complex of two or more proteins that may include other nonprotein molecules) [goid 5515] [evidence IPI] |
| P2ry12 | purinergic receptor P2Y, G-protein coupled 12 (P2ry12), mRNA. | 70839 | A receptor that binds an extracellular ligand and transmits the signal to a heterotrimeric G-protein complex. These receptors are characteristically seven-transmembrane receptors and are made up of hetero- or homodimers [goid 4930] [evidence IEA]; Combining with adenosine and transmitting the signal to a heterotrimeric G-protein complex to initiate a change in cell activity [goid 1609] [evidence IMP]; [goid 1621] [evidence IMP]; Combining with a purine nucleotide and transmitting the signal to a heterotrimeric G-protein complex to initiate a change in cell activity [goid 45028] [evidence IEA]; Combining with an extracellular or intracellular messenger to initiate a change in cell activity [goid 4872] [evidence IEA]; Mediates the transfer of a signal from the outside to the inside of a cell by means other than the introduction of the signal molecule itself into the cell [goid 4871] [evidence IEA] |
| Pabpc5 | poly A binding protein, cytoplasmic 5 (Pabpc5), mRNA. | 93728 | Elemental activities, such as catalysis or binding, describing the actions of a gene product at the molecular level. A given gene product may exhibit one or more molecular functions [goid 3674] [evidence ND ] |
| Pacsin3 | protein kinase C and casein kinase substrate in neurons 3 (Pacsin3), mRNA. | 80708 | Interacting selectively with any protein or protein complex (a complex of two or more proteins that may include other nonprotein molecules) [goid 5515] [evidence IPI]; Interacting selectively with any protein component of any cytoskeleton (actin, microtubule, or intermediate filament cytoskeleton) [goid 8092] [evidence IDA]; Catalysis of the transfer of a phosphate group, usually from ATP, to a substrate molecule [goid 16301] [evidence IEA] |
| Palmd | palmdelphin (Palmd), mRNA. | 114301 |  |
| Pdhx | pyruvate dehydrogenase complex, component X (Pdhx), nuclear gene encoding mitochondrial protein, mRNA. | 27402 | Catalysis of the transfer of a group, e.g. a methyl group, glycosyl group, acyl group, phosphorus-containing, or other groups, from one compound (generally regarded as the donor) to another compound (generally regarded as the acceptor). Transferase is the systematic name for any enzyme of EC class 2 [goid 16740] [evidence IEA]; Catalysis of the generalized reaction: acyl-carrier + reactant = acyl-reactant + carrier [goid 8415] [evidence IEA]; Interacting selectively with lipoic acid, 1,2-dithiolane-3-pentanoic acid [goid 31405] [evidence IEA]; Interacting selectively with any protein or protein complex (a complex of two or more proteins that may include other nonprotein molecules) [goid 5515] [evidence IEA] |
| Pdlim3 | PDZ and LIM domain 3 (Pdlim3), mRNA. | 53318 | Interacting selectively with any metal ion [goid 46872] [evidence IEA]; Interacting selectively with zinc (Zn) ions [goid 8270] [evidence IEA]; Interacting selectively with any protein or protein complex (a complex of two or more proteins that may include other nonprotein molecules) [goid 5515] [evidence TAS]; The action of a molecule that contributes to the structural integrity of a muscle fiber [goid 8307] [evidence IDA]; The action of a molecule that contributes to the structural integrity of a muscle fiber [goid 8307] [evidence TAS]; Interacting selectively with any protein component of any cytoskeleton (actin, microtubule, or intermediate filament cytoskeleton) [goid 8092] [evidence IPI] |
| Pdzd11 | PDZ domain containing 11 (Pdzd11), mRNA. | 72621 | Interacting selectively with any protein or protein complex (a complex of two or more proteins that may include other nonprotein molecules) [goid 5515] [evidence IEA] |
| Pfdn2 | prefoldin 2 (Pfdn2), mRNA. | 18637 | Interacting selectively with an unfolded protein [goid 51082] [evidence IEA] |
| Pgs1 | phosphatidylglycerophosphate synthase 1 (Pgs1), mRNA. | 74451 | Catalysis of the transfer of a group, e.g. a methyl group, glycosyl group, acyl group, phosphorus-containing, or other groups, from one compound (generally regarded as the donor) to another compound (generally regarded as the acceptor). Transferase is the systematic name for any enzyme of EC class 2 [goid 16740] [evidence IEA]; Interacting selectively with a nucleotide, any compound consisting of a nucleoside that is esterified with (ortho)phosphate or an oligophosphate at any hydroxyl group on the ribose or deoxyribose moiety [goid 166] [evidence IEA]; Interacting selectively with ATP, adenosine 5'-triphosphate, a universally important coenzyme and enzyme regulator [goid 5524] [evidence IEA]; Catalysis of a biochemical reaction at physiological temperatures. In biologically catalyzed reactions, the reactants are known as substrates, and the catalysts are naturally occurring macromolecular substances known as enzymes. Enzymes possess specific binding sites for substrates, and are usually composed wholly or largely of protein, but RNA that has catalytic activity (ribozyme) is often also regarded as enzymatic [goid 3824] [evidence IEA]; Catalysis of the transfer of a substituted phosphate group, other than diphosphate or nucleotidyl residues, from one compound (donor) to a another (acceptor) [goid 16780] [evidence IEA]; Catalysis of the reaction: CDP-diacylglycerol + glycerol 3-phosphate = CMP + 3-(3-phosphatidyl)-glycerol 1-phosphate [goid 8444] [evidence IEA] |
| Phf11 | PHD finger protein 11 (Phf11), mRNA. | 219131 |  |
| Pitx2 | paired-like homeodomain transcription factor 2 (Pitx2), transcript variant 3, mRNA. | 18741 | Interacting selectively with DNA (deoxyribonucleic acid) [goid 3677] [evidence IEA]; Interacting selectively with DNA of a specific nucleotide composition, e.g. GC-rich DNA binding, or with a specific sequence motif or type of DNA e.g. promotor binding or rDNA binding [goid 43565] [evidence IEA]; Interacting selectively with chromatin, the network of fibers of DNA and protein that make up the chromosomes of the eukaryotic nucleus during interphase [goid 3682] [evidence IDA]; Interacting selectively with any protein or protein complex (a complex of two or more proteins that may include other nonprotein molecules) [goid 5515] [evidence IPI]; The function of binding to a specific DNA sequence in order to modulate transcription. The transcription factor may or may not also interact selectively with a protein or macromolecular complex [goid 3700] [evidence IDA]; The function of binding to a specific DNA sequence in order to modulate transcription. The transcription factor may or may not also interact selectively with a protein or macromolecular complex [goid 3700] [evidence ISO]; The function of binding to a specific DNA sequence in order to modulate transcription. The transcription factor may or may not also interact selectively with a protein or macromolecular complex [goid 3700] [evidence IDA]; Interacting selectively with a transcription factor, any protein required to initiate or regulate transcription [goid 8134] [evidence ISO] |
| Pkn2 | protein kinase N2 (Pkn2), mRNA. | 109333 | Catalysis of the transfer of a phosphate group, usually from ATP, to a substrate molecule [goid 16301] [evidence IEA]; Interacting selectively with a nucleotide, any compound consisting of a nucleoside that is esterified with (ortho)phosphate or an oligophosphate at any hydroxyl group on the ribose or deoxyribose moiety [goid 166] [evidence IEA]; Catalysis of the reaction: ATP + a protein serine/threonine = ADP + protein serine/threonine phosphate [goid 4674] [evidence IEA]; Catalysis of the transfer of a group, e.g. a methyl group, glycosyl group, acyl group, phosphorus-containing, or other groups, from one compound (generally regarded as the donor) to another compound (generally regarded as the acceptor). Transferase is the systematic name for any enzyme of EC class 2 [goid 16740] [evidence IEA]; Interacting selectively with ATP, adenosine 5'-triphosphate, a universally important coenzyme and enzyme regulator [goid 5524] [evidence IEA]; Catalysis of the phosphorylation of an amino acid residue in a protein, usually according to the reaction: a protein + ATP = a phosphoprotein + ADP [goid 4672] [evidence IEA]; Catalysis of the reaction: ATP + a protein = ADP + a phosphoprotein, with a requirement for diacylglycerol [goid 4697] [evidence IEA] |
| Pla2g15 | phospholipase A2, group XV (Pla2g15), mRNA. | 192654 | Catalysis of the hydrolysis of various bonds, e.g. C-O, C-N, C-C, phosphoric anhydride bonds, etc. Hydrolase is the systematic name for any enzyme of EC class 3 [goid 16787] [evidence IEA]; Catalysis of the generalized reaction: acyl-carrier + reactant = acyl-reactant + carrier [goid 8415] [evidence IEA]; Catalysis of the reaction: 2-lysophosphatidylcholine + H2O = glycerophosphocholine + a carboxylate [goid 4622] [evidence IDA]; Catalysis of the transfer of a group, e.g. a methyl group, glycosyl group, acyl group, phosphorus-containing, or other groups, from one compound (generally regarded as the donor) to another compound (generally regarded as the acceptor). Transferase is the systematic name for any enzyme of EC class 2 [goid 16740] [evidence IEA]; Catalysis of the reaction: phosphatidylcholine + a sterol = a sterol ester + 1-acylglycerophosphocholine [goid 4607] [evidence IEA]; Catalysis of the reaction: phosphatidylcholine + H2O = 1-acylglycerophosphocholine + a carboxylate; does not require Ca2+ [goid 47499] [evidence IDA] |
| Plagl1 | pleiomorphic adenoma gene-like 1 (Plagl1), mRNA. | 22634 | Interacting selectively with any protein or protein complex (a complex of two or more proteins that may include other nonprotein molecules) [goid 5515] [evidence IPI]; Interacting selectively with DNA (deoxyribonucleic acid) [goid 3677] [evidence ISO] |
| Pld5 | phospholipase D family, member 5 (Pld5), mRNA. XM_899968 XM_915002 XM_923506 XM_923510 XM_923521 XM_923527 | 319455 | Catalysis of a biochemical reaction at physiological temperatures. In biologically catalyzed reactions, the reactants are known as substrates, and the catalysts are naturally occurring macromolecular substances known as enzymes. Enzymes possess specific binding sites for substrates, and are usually composed wholly or largely of protein, but RNA that has catalytic activity (ribozyme) is often also regarded as enzymatic [goid 3824] [evidence IEA] |
| Plec1 | plectin 1 (Plec1), transcript variant 11, mRNA. | 18810 | Interacting selectively with monomeric or multimeric forms of actin, including actin filaments [goid 3779] [evidence IEA]; The action of a molecule that contributes to the structural integrity of a complex or assembly within or outside a cell [goid 5198] [evidence IEA] |
| Plek | pleckstrin (Plek), mRNA. | 56193 |  |
| Plekhg5 | pleckstrin homology domain containing, family G (with RhoGef domain) member 5 (Plekhg5), mRNA. | 269608 | Stimulates the exchange of guanyl nucleotides by a GTPase of the Rho family. Under normal cellular physiological conditions, the concentration of GTP is higher than that of GDP, favoring the replacement of GDP by GTP in association with the GTPase [goid 5089] [evidence IDA]; Interacting selectively with any protein or protein complex (a complex of two or more proteins that may include other nonprotein molecules) [goid 5515] [evidence IPI] |
| Pofut1 | protein O-fucosyltransferase 1 (Pofut1), transcript variant 2, mRNA. | 140484 | Catalysis of the transfer of a fucosyl group to an acceptor molecule, typically another carbohydrate or a lipid [goid 8417] [evidence ISO]; Interacting selectively with manganese (Mn) ions [goid 30145] [evidence IEA]; Catalysis of the transfer of a group, e.g. a methyl group, glycosyl group, acyl group, phosphorus-containing, or other groups, from one compound (generally regarded as the donor) to another compound (generally regarded as the acceptor). Transferase is the systematic name for any enzyme of EC class 2 [goid 16740] [evidence IEA]; Catalysis of the transfer of a glycosyl group from one compound (donor) to another (acceptor) [goid 16757] [evidence IEA]; Catalysis of the transfer of an alpha-L-fucosyl residue from GDP- beta-L-fucose to the serine hydroxy group of a protein acceptor [goid 46922] [evidence IEA] |
| Popdc2 | popeye domain containing 2 (Popdc2), transcript variant 2, mRNA. | 64082 |  |
| Ppp5c | protein phosphatase 5, catalytic subunit (Ppp5c), mRNA. | 19060 | Catalysis of the hydrolysis of various bonds, e.g. C-O, C-N, C-C, phosphoric anhydride bonds, etc. Hydrolase is the systematic name for any enzyme of EC class 3 [goid 16787] [evidence IEA]; Interacting selectively with manganese (Mn) ions [goid 30145] [evidence IEA]; Catalysis of the reaction: a phosphoprotein + H2O = a protein + phosphate. Together with protein kinases, these enzymes control the state of phosphorylation of cell proteins and thereby provide an important mechanism for regulating cellular activity [goid 4721] [evidence IMP]; Interacting selectively with iron (Fe) ions [goid 5506] [evidence IEA]; Interacting selectively with any metal ion [goid 46872] [evidence IEA]; The selective, often stoichiometric, interaction of a molecule with one or more specific sites on another molecule [goid 5488] [evidence IEA]; Interacting selectively with any protein or protein complex (a complex of two or more proteins that may include other nonprotein molecules) [goid 5515] [evidence IPI] |
| Prf1 | perforin 1 (pore forming protein) (Prf1), mRNA. | 18646 | Interacting selectively with calcium ions (Ca2+) [goid 5509] [evidence IEA] |
| Prss34 | protease, serine, 34 (Prss34), mRNA. | 328780 | Catalysis of the hydrolysis of peptide bonds in a polypeptide chain by a catalytic mechanism that involves a catalytic triad consisting of a serine nucleophile that is activated by a proton relay involving an acidic residue (e.g. aspartate or glutamate) and a basic residue (usually histidine) [goid 8236] [evidence IDA]; Catalysis of the hydrolysis of a peptide bond. A peptide bond is a covalent bond formed when the carbon atom from the carboxyl group of one amino acid shares electrons with the nitrogen atom from the amino group of a second amino acid [goid 8233] [evidence IEA] |
| Prss36 | protease, serine, 36 (Prss36), mRNA. | 77613 | Catalysis of the hydrolysis of various bonds, e.g. C-O, C-N, C-C, phosphoric anhydride bonds, etc. Hydrolase is the systematic name for any enzyme of EC class 3 [goid 16787] [evidence IEA]; Catalysis of the hydrolysis of a peptide bond. A peptide bond is a covalent bond formed when the carbon atom from the carboxyl group of one amino acid shares electrons with the nitrogen atom from the amino group of a second amino acid [goid 8233] [evidence IEA]; Catalysis of the hydrolysis of internal, alpha-peptide bonds in a polypeptide chain by a catalytic mechanism that involves a catalytic triad consisting of a serine nucleophile that is activated by a proton relay involving an acidic residue (e.g. aspartate or glutamate) and a basic residue (usually histidine) [goid 4252] [evidence ISA] |
| Psg23 | pregnancy-specific glycoprotein 23 (Psg23), mRNA. | 56868 |  |
| Psmd4 | proteasome (prosome, macropain) 26S subunit, non-ATPase, 4 (Psmd4), mRNA. | 19185 | Elemental activities, such as catalysis or binding, describing the actions of a gene product at the molecular level. A given gene product may exhibit one or more molecular functions [goid 3674] [evidence ND ] |
| Ptp4a2 | protein tyrosine phosphatase 4a2 (Ptp4a2), mRNA. | 19244 | Catalysis of the hydrolysis of various bonds, e.g. C-O, C-N, C-C, phosphoric anhydride bonds, etc. Hydrolase is the systematic name for any enzyme of EC class 3 [goid 16787] [evidence IEA]; Catalysis of the reaction: a phosphoprotein + H2O = a protein + phosphate. Together with protein kinases, these enzymes control the state of phosphorylation of cell proteins and thereby provide an important mechanism for regulating cellular activity [goid 4721] [evidence IEA]; Catalysis of the reaction: protein tyrosine phosphate + H2O = protein tyrosine + phosphate [goid 4725] [evidence IEA]; Catalysis of the hydrolysis of phosphoric monoesters, releasing inorganic phosphate [goid 16791] [evidence IEA] |
| Pxt1 | peroxisomal, testis specific 1 (Pxt1), mRNA. | 69307 |  |
| R3hdm1 | R3H domain 1 (binds single-stranded nucleic acids) (R3hdm1), mRNA. | 226412 |  |
| Rab19 | RAB19, member RAS oncogene family (Rab19), mRNA. | 19331 | Interacting selectively with GTP, guanosine triphosphate [goid 5525] [evidence IEA]; Interacting selectively with a nucleotide, any compound consisting of a nucleoside that is esterified with (ortho)phosphate or an oligophosphate at any hydroxyl group on the ribose or deoxyribose moiety [goid 166] [evidence IEA] |
| Rabgef1 | RAB guanine nucleotide exchange factor (GEF) 1 (Rabgef1), mRNA. | 56715 | Interacting selectively with any metal ion [goid 46872] [evidence IEA]; Interacting selectively with zinc (Zn) ions [goid 8270] [evidence IEA]; Interacting selectively with DNA (deoxyribonucleic acid) [goid 3677] [evidence IEA]; Elemental activities, such as catalysis or binding, describing the actions of a gene product at the molecular level. A given gene product may exhibit one or more molecular functions [goid 3674] [evidence ND ] |
| Rapgefl1 | Rap guanine nucleotide exchange factor (GEF)-like 1 (Rapgefl1), mRNA. | 268480 | Stimulates the exchange of guanyl nucleotides by a GTPase. Under normal cellular physiological conditions, the concentration of GTP is higher than that of GDP, favoring the replacement of GDP by GTP in association with the GTPase [goid 5085] [evidence IEA] |
| Rbak | RB-associated KRAB repressor (Rbak), transcript variant 1, mRNA. | 57782 | Interacting selectively with any metal ion [goid 46872] [evidence IEA]; Interacting selectively with zinc (Zn) ions [goid 8270] [evidence IEA]; Interacting selectively with any nucleic acid [goid 3676] [evidence IEA] |
| Rbbp7 | retinoblastoma binding protein 7 (Rbbp7), mRNA. | 245688 | Interacting selectively with any protein or protein complex (a complex of two or more proteins that may include other nonprotein molecules) [goid 5515] [evidence IPI]; Any transcription regulator activity that prevents or downregulates transcription [goid 16564] [evidence IDA] |
| Rbm12b | RNA binding motif protein 12B (Rbm12b), mRNA. | 72397 | Interacting selectively with an RNA molecule or a portion thereof [goid 3723] [evidence IEA]; Interacting selectively with any nucleic acid [goid 3676] [evidence IEA]; Interacting selectively with a nucleotide, any compound consisting of a nucleoside that is esterified with (ortho)phosphate or an oligophosphate at any hydroxyl group on the ribose or deoxyribose moiety [goid 166] [evidence IEA] |
| Rbm14 | RNA binding motif protein 14 (Rbm14), mRNA. | 56275 | Interacting selectively with an RNA molecule or a portion thereof [goid 3723] [evidence IEA]; Interacting selectively with any nucleic acid [goid 3676] [evidence IEA]; Interacting selectively with a nucleotide, any compound consisting of a nucleoside that is esterified with (ortho)phosphate or an oligophosphate at any hydroxyl group on the ribose or deoxyribose moiety [goid 166] [evidence IEA]; Elemental activities, such as catalysis or binding, describing the actions of a gene product at the molecular level. A given gene product may exhibit one or more molecular functions [goid 3674] [evidence ND ] |
| Rchy1 | ring finger and CHY zinc finger domain containing 1 (Rchy1), mRNA. | 68098 | Interacting selectively with any metal ion [goid 46872] [evidence IEA]; Interacting selectively with zinc (Zn) ions [goid 8270] [evidence IEA]; Interacting selectively with any protein or protein complex (a complex of two or more proteins that may include other nonprotein molecules) [goid 5515] [evidence IEA] |
| Rhbdl2 | rhomboid, veinlet-like 2 (Drosophila) (Rhbdl2), mRNA. | 230726 |  |
| Rnf113a2 | ring finger protein 113A2 (Rnf113a2), mRNA. | 66381 | Interacting selectively with any metal ion [goid 46872] [evidence IEA]; Interacting selectively with zinc (Zn) ions [goid 8270] [evidence IEA] |
| Rnf182 | ring finger protein 182 (Rnf182), mRNA. | 328234 | Interacting selectively with any metal ion [goid 46872] [evidence IEA]; Interacting selectively with zinc (Zn) ions [goid 8270] [evidence IEA]; Interacting selectively with any protein or protein complex (a complex of two or more proteins that may include other nonprotein molecules) [goid 5515] [evidence IEA] |
| Rngtt | RNA guanylyltransferase and 5'-phosphatase (Rngtt), mRNA. | 24018 | Catalysis of the hydrolysis of various bonds, e.g. C-O, C-N, C-C, phosphoric anhydride bonds, etc. Hydrolase is the systematic name for any enzyme of EC class 3 [goid 16787] [evidence IEA]; Catalysis of a biochemical reaction at physiological temperatures. In biologically catalyzed reactions, the reactants are known as substrates, and the catalysts are naturally occurring macromolecular substances known as enzymes. Enzymes possess specific binding sites for substrates, and are usually composed wholly or largely of protein, but RNA that has catalytic activity (ribozyme) is often also regarded as enzymatic [goid 3824] [evidence IEA]; Catalysis of the transfer of a group, e.g. a methyl group, glycosyl group, acyl group, phosphorus-containing, or other groups, from one compound (generally regarded as the donor) to another compound (generally regarded as the acceptor). Transferase is the systematic name for any enzyme of EC class 2 [goid 16740] [evidence IEA]; Catalysis of the reaction: 5'-phosphopolynucleotide + H2O = polynucleotide + phosphate [goid 4651] [evidence IEA]; Catalysis of the transfer of a nucleotidyl group to a reactant [goid 16779] [evidence IEA]; Catalysis of the reaction: GTP + (5')pp-Pur-mRNA = diphosphate + G(5')ppp-Pur-mRNA; G(5')ppp-Pur-mRNA is mRNA containing a guanosine residue linked 5' through three phosphates to the 5' position of the terminal residue [goid 4484] [evidence IEA]; Catalysis of the hydrolysis of phosphoric monoesters, releasing inorganic phosphate [goid 16791] [evidence IEA]; Catalysis of the reaction: protein serine/threonine/tyrosine phosphate + H2O = protein serine/threonine/tyrosine + phosphate [goid 8138] [evidence IEA]; Catalysis of the reaction: protein tyrosine phosphate + H2O = protein tyrosine + phosphate [goid 4725] [evidence IEA] |
| Rnu6 | U6 small nuclear RNA (Rnu6), non-coding RNA. | 19862 |  |
| Rod1 | ROD1 regulator of differentiation 1 (S. pombe) (Rod1), transcript variant 1, mRNA. | 230257 | Interacting selectively with an RNA molecule or a portion thereof [goid 3723] [evidence IEA]; Interacting selectively with any nucleic acid [goid 3676] [evidence IEA]; Interacting selectively with a nucleotide, any compound consisting of a nucleoside that is esterified with (ortho)phosphate or an oligophosphate at any hydroxyl group on the ribose or deoxyribose moiety [goid 166] [evidence IEA] |
| Rpl23 | ribosomal protein L23 (Rpl23), mRNA. | 65019 | The action of a molecule that contributes to the structural integrity of the ribosome [goid 3735] [evidence ISA] |
| Rps3 | ribosomal protein S3 (Rps3), mRNA. | 27050 | Interacting selectively with an RNA molecule or a portion thereof [goid 3723] [evidence IEA]; The action of a molecule that contributes to the structural integrity of the ribosome [goid 3735] [evidence IEA] |
| Sdhb | succinate dehydrogenase complex, subunit B, iron sulfur (Ip) (Sdhb), nuclear gene encoding mitochondrial protein, mRNA. | 67680 | Interacting selectively with a 2 iron, 2 sulfur (2Fe-2S) cluster; this cluster consists of two iron atoms, with two inorganic sulfur atoms found between the irons and acting as bridging ligands [goid 51537] [evidence IEA]; Interacting selectively with a 3 iron, 4 sulfur (3Fe-4S) cluster; this cluster consists of three iron atoms, with the inorganic sulfur atoms found between the irons and acting as bridging ligands. It is essentially a 4Fe-4S cluster with one iron missing [goid 51538] [evidence IEA]; Interacting selectively with a 4 iron, 4 sulfur (4Fe-4S) cluster; this cluster consists of four iron atoms, with the inorganic sulfur atoms found between the irons and acting as bridging ligands [goid 51539] [evidence IEA]; Interacting selectively with an iron-sulfur cluster, a combination of iron and sulfur atoms [goid 51536] [evidence IEA]; Interacting selectively with iron (Fe) ions [goid 5506] [evidence IEA]; Interacting selectively with any metal ion [goid 46872] [evidence IEA]; Catalysis of an oxidation-reduction (redox) reaction, a reversible chemical reaction in which the oxidation state of an atom or atoms within a molecule is altered. One substrate acts as a hydrogen or electron donor and becomes oxidized, while the other acts as hydrogen or electron acceptor and becomes reduced [goid 16491] [evidence IEA]; Any molecular entity that serves as an electron acceptor and electron donor in an electron transport system [goid 9055] [evidence IEA]; Catalysis of the reaction: succinate + quinone = fumarate + quinol [goid 8177] [evidence IEA] |
| Sdk1 | sidekick homolog 1 (chicken) (Sdk1), mRNA. | 330222 | Interacting selectively with any protein or protein complex (a complex of two or more proteins that may include other nonprotein molecules) [goid 5515] [evidence IEA] |
| Sema4f | sema domain, immunoglobulin domain (Ig), TM domain, and short cytoplasmic domain (Sema4f), mRNA. | 20355 | Interacting selectively with any protein or protein complex (a complex of two or more proteins that may include other nonprotein molecules) [goid 5515] [evidence IPI] |
| Serbp1 | Serpine1 mRNA binding protein 1 (Serbp1), mRNA. | 66870 | Interacting selectively with an RNA molecule or a portion thereof [goid 3723] [evidence IEA] |
| Serpina3c | serine (or cysteine) peptidase inhibitor, clade A, member 3C (Serpina3c), mRNA. | 16625 | Stops, prevents or reduces the activity of an endopeptidase, any enzyme that hydrolyzes nonterminal peptide bonds in polypeptides [goid 4866] [evidence IEA]; Stops, prevents or reduces the activity of serine-type endopeptidases, enzymes that catalyze the hydrolysis of nonterminal peptide bonds in a polypeptide chain; a serine residue (and a histidine residue) are at the active center of the enzyme [goid 4867] [evidence IEA] |
| Serpinb3b | serine (or cysteine) peptidase inhibitor, clade B (ovalbumin), member 3B (Serpinb3b), mRNA. | 383548 | [goid 4869] [evidence IDA]; Stops, prevents or reduces the activity of serine-type endopeptidases, enzymes that catalyze the hydrolysis of nonterminal peptide bonds in a polypeptide chain; a serine residue (and a histidine residue) are at the active center of the enzyme [goid 4867] [evidence IDA] |
| Serpinf1 | serine (or cysteine) peptidase inhibitor, clade F, member 1 (Serpinf1), mRNA. | 20317 | Stops, prevents or reduces the activity of serine-type endopeptidases, enzymes that catalyze the hydrolysis of nonterminal peptide bonds in a polypeptide chain; a serine residue (and a histidine residue) are at the active center of the enzyme [goid 4867] [evidence IEA] |
| Sf3b3 | splicing factor 3b, subunit 3 (Sf3b3), mRNA. | 101943 | Interacting selectively with any nucleic acid [goid 3676] [evidence IEA] |
| Sgk1 | serum/glucocorticoid regulated kinase 1 (Sgk1), mRNA. | 20393 | Catalysis of the transfer of a phosphate group, usually from ATP, to a substrate molecule [goid 16301] [evidence IEA]; Interacting selectively with a nucleotide, any compound consisting of a nucleoside that is esterified with (ortho)phosphate or an oligophosphate at any hydroxyl group on the ribose or deoxyribose moiety [goid 166] [evidence IEA]; Catalysis of the reaction: ATP + a protein serine/threonine = ADP + protein serine/threonine phosphate [goid 4674] [evidence IEA]; Catalysis of the transfer of a group, e.g. a methyl group, glycosyl group, acyl group, phosphorus-containing, or other groups, from one compound (generally regarded as the donor) to another compound (generally regarded as the acceptor). Transferase is the systematic name for any enzyme of EC class 2 [goid 16740] [evidence IEA]; Interacting selectively with ATP, adenosine 5'-triphosphate, a universally important coenzyme and enzyme regulator [goid 5524] [evidence IEA]; Interacting selectively with any protein or protein complex (a complex of two or more proteins that may include other nonprotein molecules) [goid 5515] [evidence IPI]; Catalysis of the phosphorylation of an amino acid residue in a protein, usually according to the reaction: a protein + ATP = a phosphoprotein + ADP [goid 4672] [evidence IDA] |
| Shc1 | src homology 2 domain-containing transforming protein C1 (Shc1), mRNA. | 20416 | Interacting selectively with any protein or protein complex (a complex of two or more proteins that may include other nonprotein molecules) [goid 5515] [evidence IPI] |
| Siglech | sialic acid binding Ig-like lectin H (Siglech), mRNA. | 233274 |  |
| Sip1 | survivor of motor neuron protein interacting protein 1 (Sip1), mRNA. | 66603 |  |
| Slc1a7 | solute carrier family 1 (glutamate transporter), member 7 (Slc1a7), mRNA. | 242607 | Enables the active transport of a solute across a membrane by a mechanism whereby two or more species are transported together in the same direction in a tightly coupled process not directly linked to a form of energy other than chemiosmotic energy [goid 15293] [evidence IEA]; Catalysis of the transfer of a solute or solutes from one side of a membrane to the other according to the reaction: dicarboxylate(out) + Na+(out) = dicarboxylate(in) + Na+(in) [goid 17153] [evidence IEA] |
| Slc35f4 | solute carrier family 35, member F4 (Slc35f4), mRNA. | 75288 |  |
| Slc36a3 | solute carrier family 36 (proton/amino acid symporter), member 3 (Slc36a3), mRNA. | 215332 |  |
| Slc45a4 | solute carrier family 45, member 4 (Slc45a4), mRNA. XM_898255 XM_898268 XM_907823 XM_920555 XM_920562 XM_920569 XM_920578 XM_920594 | 106068 |  |
| Slc9a6 | solute carrier family 9 (sodium/hydrogen exchanger), member 6 (Slc9a6), mRNA. | 236794 | Enables the active transport of a solute across a membrane by a mechanism whereby two or more species are transported in opposite directions in a tightly coupled process not directly linked to a form of energy other than chemiosmotic energy [goid 15297] [evidence IEA] |
| Slco1a4 | solute carrier organic anion transporter family, member 1a4 (Slco1a4), mRNA. | 28250 | Enables the directed movement of substances (such as macromolecules, small molecules, ions) into, out of, within or between cells [goid 5215] [evidence IEA]; Catalysis of the transfer of organic anions from one side of a membrane to the other. Organic anions are atoms or small molecules with a negative charge which contain carbon in covalent linkage [goid 8514] [evidence IDA] |
| Slco2b1 | solute carrier organic anion transporter family, member 2b1 (Slco2b1), mRNA. | 101488 | Enables the directed movement of substances (such as macromolecules, small molecules, ions) into, out of, within or between cells [goid 5215] [evidence IEA]; Catalysis of the transfer of organic anions from one side of a membrane to the other. Organic anions are atoms or small molecules with a negative charge which contain carbon in covalent linkage [goid 8514] [evidence ISO] |
| Slfnl1 | schlafen like 1 (Slfnl1), mRNA. | 194219 | Interacting selectively with ATP, adenosine 5'-triphosphate, a universally important coenzyme and enzyme regulator [goid 5524] [evidence IEA]; Interacting selectively with a nucleotide, any compound consisting of a nucleoside that is esterified with (ortho)phosphate or an oligophosphate at any hydroxyl group on the ribose or deoxyribose moiety [goid 166] [evidence IEA] |
| Sltm | SAFB-like, transcription modulator (Sltm), transcript variant 1, mRNA. | 66660 | Interacting selectively with an RNA molecule or a portion thereof [goid 3723] [evidence IEA]; Interacting selectively with any nucleic acid [goid 3676] [evidence IEA]; Interacting selectively with a nucleotide, any compound consisting of a nucleoside that is esterified with (ortho)phosphate or an oligophosphate at any hydroxyl group on the ribose or deoxyribose moiety [goid 166] [evidence IEA] |
| Snapc3 | small nuclear RNA activating complex, polypeptide 3 (Snapc3), mRNA. | 77634 | Interacting selectively with DNA (deoxyribonucleic acid) [goid 3677] [evidence IEA] |
| Sncg | synuclein, gamma (Sncg), mRNA. | 20618 | Elemental activities, such as catalysis or binding, describing the actions of a gene product at the molecular level. A given gene product may exhibit one or more molecular functions [goid 3674] [evidence ND ] |
| Snora65 | small nucleolar RNA, H/ACA box 65 (Snora65) on chromosome 2. | 104367 |  |
| Socs3 | suppressor of cytokine signaling 3 (Socs3), mRNA. | 12702 | Interacting selectively with any protein or protein complex (a complex of two or more proteins that may include other nonprotein molecules) [goid 5515] [evidence IPI] |
| Sorbs3 | sorbin and SH3 domain containing 3 (Sorbs3), mRNA. | 20410 | Interacting selectively with any protein or protein complex (a complex of two or more proteins that may include other nonprotein molecules) [goid 5515] [evidence IDA]; Interacting selectively with any protein or protein complex (a complex of two or more proteins that may include other nonprotein molecules) [goid 5515] [evidence IPI]; Interacting selectively with a transcription factor, any protein required to initiate or regulate transcription [goid 8134] [evidence IPI] |
| Sox7 | SRY-box containing gene 7 (Sox7), mRNA. | 20680 | Interacting selectively with DNA (deoxyribonucleic acid) [goid 3677] [evidence IEA]; The function of binding to a specific DNA sequence in order to modulate transcription. The transcription factor may or may not also interact selectively with a protein or macromolecular complex [goid 3700] [evidence IDA] |
| Spag4l | sperm associated antigen 4-like (Spag4l), mRNA. | 76407 |  |
| Spdyb | speedy homolog B (Drosophila) (Spdyb), mRNA. | 74673 | Interacting selectively with a protein kinase, any enzyme that catalyzes the transfer of a phosphate group, usually from ATP, to a protein substrate [goid 19901] [evidence IPI] |
| Sphk1 | sphingosine kinase 1 (Sphk1), transcript variant 1, mRNA. | 20698 | Interacting selectively with DNA (deoxyribonucleic acid) [goid 3677] [evidence ISA]; Interacting selectively with calmodulin, a calcium-binding protein with many roles, both in the calcium-bound and calcium-free states [goid 5516] [evidence ISA]; Interacting selectively with ATP, adenosine 5'-triphosphate, a universally important coenzyme and enzyme regulator [goid 5524] [evidence IEA]; Catalysis of the transfer of a group, e.g. a methyl group, glycosyl group, acyl group, phosphorus-containing, or other groups, from one compound (generally regarded as the donor) to another compound (generally regarded as the acceptor). Transferase is the systematic name for any enzyme of EC class 2 [goid 16740] [evidence IEA]; Catalysis of the transfer of a phosphate group, usually from ATP, to a substrate molecule [goid 16301] [evidence IEA]; Interacting selectively with a nucleotide, any compound consisting of a nucleoside that is esterified with (ortho)phosphate or an oligophosphate at any hydroxyl group on the ribose or deoxyribose moiety [goid 166] [evidence IEA]; Catalysis of the reaction: NTP + 1,2-diacylglycerol = NDP + 1,2-diacylglycerol 3-phosphate [goid 4143] [evidence IEA]; Catalysis of the reaction: ATP + sphinganine = ADP + sphinganine 1-phosphate [goid 8481] [evidence ISA]; Catalysis of the reaction: ATP + sphinganine = ADP + sphinganine 1-phosphate [goid 8481] [evidence ISO] |
| Ssbp3 | single-stranded DNA binding protein 3 (Ssbp3), transcript variant 1, mRNA. | 72475 | Interacting selectively with DNA (deoxyribonucleic acid) [goid 3677] [evidence IEA]; Plays a role in regulating transcription; may bind a promoter or enhancer DNA sequence or interact with a DNA-binding transcription factor [goid 30528] [evidence IEA]; Interacting selectively with single-stranded DNA [goid 3697] [evidence ISA] |
| Stk35 | serine/threonine kinase 35 (Stk35), transcript variant 1, mRNA. | 67333 | Catalysis of the transfer of a phosphate group, usually from ATP, to a substrate molecule [goid 16301] [evidence IEA]; Interacting selectively with a nucleotide, any compound consisting of a nucleoside that is esterified with (ortho)phosphate or an oligophosphate at any hydroxyl group on the ribose or deoxyribose moiety [goid 166] [evidence IEA]; Catalysis of the reaction: ATP + a protein serine/threonine = ADP + protein serine/threonine phosphate [goid 4674] [evidence IEA]; Catalysis of the transfer of a group, e.g. a methyl group, glycosyl group, acyl group, phosphorus-containing, or other groups, from one compound (generally regarded as the donor) to another compound (generally regarded as the acceptor). Transferase is the systematic name for any enzyme of EC class 2 [goid 16740] [evidence IEA]; Interacting selectively with ATP, adenosine 5'-triphosphate, a universally important coenzyme and enzyme regulator [goid 5524] [evidence IEA]; Catalysis of the phosphorylation of an amino acid residue in a protein, usually according to the reaction: a protein + ATP = a phosphoprotein + ADP [goid 4672] [evidence IEA] |
| Sult1d1 | sulfotransferase family 1D, member 1 (Sult1d1), mRNA. | 53315 | Catalysis of the transfer of a group, e.g. a methyl group, glycosyl group, acyl group, phosphorus-containing, or other groups, from one compound (generally regarded as the donor) to another compound (generally regarded as the acceptor). Transferase is the systematic name for any enzyme of EC class 2 [goid 16740] [evidence IEA]; Catalysis of the reaction: 3'-phosphoadenosine 5'-phosphosulfate + L-tyrosine methyl ester = adenosine 3',5'-bisphosphate + L-tyrosine methyl ester 4-sulfate [goid 17067] [evidence IEA]; Catalysis of the reaction: 3'-phosphoadenosine 5'-phosphosulfate + a phenol = adenosine 3',5'-bisphosphate + an aryl sulfate [goid 4062] [evidence IDA] |
| Suv420h1 | suppressor of variegation 4-20 homolog 1 (Drosophila) (Suv420h1), mRNA. | 225888 | Catalysis of the transfer of a group, e.g. a methyl group, glycosyl group, acyl group, phosphorus-containing, or other groups, from one compound (generally regarded as the donor) to another compound (generally regarded as the acceptor). Transferase is the systematic name for any enzyme of EC class 2 [goid 16740] [evidence IEA]; Catalysis of the transfer of a methyl group to an acceptor molecule [goid 8168] [evidence IEA]; Catalysis of the reaction: S-adenosyl-L-methionine + histone L-lysine = S-adenosyl-L-homocysteine + histone N6-methyl-L-lysine. The methylation of peptidyl-lysine in histones forms N6-methyl-L-lysine, N6,N6-dimethyl-L-lysine and N6,N6,N6-trimethyl-L-lysine derivatives [goid 18024] [evidence IEA]; Catalysis of the addition of a methyl group onto lysine at position 20 of the histone H4 protein [goid 42799] [evidence IDA] |
| Syt2 | synaptotagmin II (Syt2), mRNA. | 20980 | Interacting selectively with any metal ion [goid 46872] [evidence IEA]; Enables the directed movement of substances (such as macromolecules, small molecules, ions) into, out of, within or between cells [goid 5215] [evidence IEA]; Interacting selectively with calcium ions (Ca2+) [goid 5509] [evidence IDA]; Interacting selectively with any protein or protein complex (a complex of two or more proteins that may include other nonprotein molecules) [goid 5515] [evidence IPI]; Interacting selectively with phospholipids, a class of lipids containing phosphoric acid as a mono- or diester, in the presence of calcium [goid 5544] [evidence IDA] |
| Tapbp | TAP binding protein (Tapbp), transcript variant 1, mRNA. | 21356 |  |
| Tcfap2a | transcription factor AP-2, alpha (Tcfap2a), mRNA. | 21418 | Interacting selectively with DNA (deoxyribonucleic acid) [goid 3677] [evidence IEA]; Interacting selectively with any protein or protein complex (a complex of two or more proteins that may include other nonprotein molecules) [goid 5515] [evidence IPI]; The function of binding to a specific DNA sequence in order to modulate transcription. The transcription factor may or may not also interact selectively with a protein or macromolecular complex [goid 3700] [evidence IDA] |
| Terf2ip | telomeric repeat binding factor 2, interacting protein (Terf2ip), mRNA. | 57321 | Interacting selectively with DNA (deoxyribonucleic acid) [goid 3677] [evidence IEA] |
| Thoc4 | THO complex 4 (Thoc4), mRNA. | 21681 | Interacting selectively with an RNA molecule or a portion thereof [goid 3723] [evidence IDA]; Interacting selectively with single-stranded DNA [goid 3697] [evidence IDA]; Interacting selectively with any nucleic acid [goid 3676] [evidence IEA]; Interacting selectively with a nucleotide, any compound consisting of a nucleoside that is esterified with (ortho)phosphate or an oligophosphate at any hydroxyl group on the ribose or deoxyribose moiety [goid 166] [evidence IEA] |
| Timm9 | translocase of inner mitochondrial membrane 9 homolog (yeast) (Timm9), nuclear gene encoding mitochondrial protein, transcript variant 2, mRNA. | 30056 | Interacting selectively with zinc (Zn) ions [goid 8270] [evidence IEA]; Interacting selectively with any metal ion [goid 46872] [evidence IEA]; Interacting selectively with any protein or protein complex (a complex of two or more proteins that may include other nonprotein molecules) [goid 5515] [evidence IPI] |
| Tlcd2 | TLC domain containing 2 (Tlcd2), mRNA. | 380712 |  |
| Tmem121 | transmembrane protein 121 (Tmem121), mRNA. | 69195 | Elemental activities, such as catalysis or binding, describing the actions of a gene product at the molecular level. A given gene product may exhibit one or more molecular functions [goid 3674] [evidence ND ] |
| Tmem180 | transmembrane protein 180 (Tmem180), mRNA. | 75146 |  |
| Tmem184a | transmembrane protein 184a (Tmem184a), mRNA. | 231832 |  |
| Tmem2 | transmembrane protein 2 (Tmem2), transcript variant 1, mRNA. | 83921 | Elemental activities, such as catalysis or binding, describing the actions of a gene product at the molecular level. A given gene product may exhibit one or more molecular functions [goid 3674] [evidence ND ] |
| Tmem33 | transmembrane protein 33 (Tmem33), transcript variant 1, mRNA. | 67878 | Interacting selectively with any protein or protein complex (a complex of two or more proteins that may include other nonprotein molecules) [goid 5515] [evidence IPI] |
| Tmem38b | transmembrane protein 38B (Tmem38b), mRNA. | 52076 | Interacting selectively with potassium (K+) ions [goid 30955] [evidence IEA]; Catalysis of facilitated diffusion of an ion (by an energy-independent process) by passage through a transmembrane aqueous pore or channel without evidence for a carrier-mediated mechanism [goid 5216] [evidence IEA]; Catalysis of facilitated diffusion of a potassium ion (by an energy-independent process) involving passage through a transmembrane aqueous pore or channel without evidence for a carrier-mediated mechanism [goid 5267] [evidence IEA]; Catalysis of the energy-independent passage of cations across a lipid bilayer down a concentration gradient [goid 5261] [evidence IEA] |
| Tmem77 | transmembrane protein 77 (Tmem77), transcript variant 2, mRNA. | 67171 |  |
| Tnfrsf10b | tumor necrosis factor receptor superfamily, member 10b (Tnfrsf10b), mRNA. | 21933 | Combining with an extracellular or intracellular messenger to initiate a change in cell activity [goid 4872] [evidence IEA]; Combining with an extracellular or intracellular messenger to initiate a change in cell activity, and spanning to the membrane of either the cell or an organelle [goid 4888] [evidence IEA]; Interacting selectively with any protein or protein complex (a complex of two or more proteins that may include other nonprotein molecules) [goid 5515] [evidence ISO]; Increases the rate of proteolysis catalyzed by a caspase [goid 8656] [evidence RCA]; Interacting selectively with TRAIL (TNF-related apoptosis inducing ligand), a member of the tumor necrosis factor ligand family that rapidly induces apoptosis in a variety of transformed cell lines [goid 45569] [evidence RCA] |
| Tnxb | tenascin XB (Tnxb), mRNA. | 81877 | Interacting selectively with collagen, a group of fibrous proteins of very high tensile strength that form the main component of connective tissue in animals. Collagen is highly enriched in glycine (some regions are 33% glycine) and proline, occurring predominantly as 3-hydroxyproline (about 20%) [goid 5518] [evidence IDA]; Interacting selectively with heparin, any member of a group of glycosaminoglycans found mainly as an intracellular component of mast cells and which consist predominantly of alternating alpha1-4-linked D-galactose and N-acetyl-D-glucosamine-6-sulfate residues [goid 8201] [evidence IDA] |
| Trpc4ap | transient receptor potential cation channel, subfamily C, member 4 associated protein (Trpc4ap), mRNA. | 56407 | Combining with an extracellular or intracellular messenger to initiate a change in cell activity [goid 4872] [evidence IEA] |
| Trub1 | TruB pseudouridine (psi) synthase homolog 1 (E. coli) (Trub1), transcript variant 1, mRNA. | 72133 | Catalysis of the geometric or structural changes within one molecule. Isomerase is the systematic name for any enzyme of EC class 5 [goid 16853] [evidence IEA]; Catalysis of the reaction: RNA uridine = RNA pseudouridine. Conversion of uridine in an RNA molecule to pseudouridine by rotation of the C1'-N-1 glycosidic bond of uridine in RNA to a C1'-C5 [goid 9982] [evidence IEA] |
| Tsc1 | tuberous sclerosis 1 (Tsc1), mRNA. | 64930 | Interacting selectively with a protein N-terminus, the end of any peptide chain at which the 2-amino (or 2-imino) function of a constituent amino acid is not attached in peptide linkage to another amino-acid residue [goid 47485] [evidence IPI] |
| Txndc11 | thioredoxin domain containing 11 (Txndc11), transcript variant 1, mRNA. | 106200 |  |
| Tyms | thymidylate synthase (Tyms), mRNA. | 22171 | Catalysis of the transfer of a methyl group to an acceptor molecule [goid 8168] [evidence IEA]; Catalysis of the transfer of a group, e.g. a methyl group, glycosyl group, acyl group, phosphorus-containing, or other groups, from one compound (generally regarded as the donor) to another compound (generally regarded as the acceptor). Transferase is the systematic name for any enzyme of EC class 2 [goid 16740] [evidence IEA]; Catalysis of the reaction: 5,10-methylenetetrahydrofolate + dUMP = dihydrofolate + dTMP [goid 4799] [evidence IEA] |
| Ubap2l | ubiquitin associated protein 2-like (Ubap2l), transcript variant 2, mRNA. | 74383 |  |
| Ube2d1 | ubiquitin-conjugating enzyme E2D 1, UBC4/5 homolog (yeast) (Ube2d1), mRNA. | 216080 | Catalysis of the ligation of two substances with concomitant breaking of a diphosphate linkage, usually in a nucleoside triphosphate. Ligase is the systematic name for any enzyme of EC class 6 [goid 16874] [evidence IEA]; Catalysis of ATP-dependent isopeptide bond formation between the carboxy-terminal residues of a small conjugating protein such as ubiquitin or a ubiquitin-like protein, and a substrate lysine residue. This function may be performed alone or in conjunction with an E3, ubiquitin-like protein ligase [goid 19787] [evidence IEA]; Catalysis of the reaction: ATP + ubiquitin + protein lysine = AMP + diphosphate + protein N-ubiquityllysine [goid 4842] [evidence IEA] |
| Ube2g1 | ubiquitin-conjugating enzyme E2G 1 (UBC7 homolog, C. elegans) (Ube2g1), mRNA. | 67128 | Catalysis of the ligation of two substances with concomitant breaking of a diphosphate linkage, usually in a nucleoside triphosphate. Ligase is the systematic name for any enzyme of EC class 6 [goid 16874] [evidence IEA]; Catalysis of ATP-dependent isopeptide bond formation between the carboxy-terminal residues of a small conjugating protein such as ubiquitin or a ubiquitin-like protein, and a substrate lysine residue. This function may be performed alone or in conjunction with an E3, ubiquitin-like protein ligase [goid 19787] [evidence IEA]; Catalysis of the reaction: ATP + ubiquitin + protein lysine = AMP + diphosphate + protein N-ubiquityllysine [goid 4842] [evidence IEA] |
| Ube2v2 | ubiquitin-conjugating enzyme E2 variant 2 (Ube2v2), mRNA. | 70620 | Interacting selectively with any protein or protein complex (a complex of two or more proteins that may include other nonprotein molecules) [goid 5515] [evidence IPI]; Catalysis of ATP-dependent isopeptide bond formation between the carboxy-terminal residues of a small conjugating protein such as ubiquitin or a ubiquitin-like protein, and a substrate lysine residue. This function may be performed alone or in conjunction with an E3, ubiquitin-like protein ligase [goid 19787] [evidence IEA] |
| Unc119 | unc-119 homolog (C. elegans) (Unc119), mRNA. XM_001004318 | 22248 |  |
| Usp12 | ubiquitin specific peptidase 12 (Usp12), mRNA. | 22217 | Catalysis of the hydrolysis of various bonds, e.g. C-O, C-N, C-C, phosphoric anhydride bonds, etc. Hydrolase is the systematic name for any enzyme of EC class 3 [goid 16787] [evidence IEA]; Catalysis of the hydrolysis of a peptide bond. A peptide bond is a covalent bond formed when the carbon atom from the carboxyl group of one amino acid shares electrons with the nitrogen atom from the amino group of a second amino acid [goid 8233] [evidence IEA]; Catalysis of the hydrolysis of peptide bonds in a polypeptide chain by a mechanism in which the sulfhydryl group of a cysteine residue at the active center acts as a nucleophile [goid 8234] [evidence IEA]; Catalysis of the reaction: ubiquitin C-terminal thiolester + H2O = ubiquitin + a thiol. Hydrolysis of esters, including those formed between thiols such as dithiothreitol or glutathione and the C-terminal glycine residue of the polypeptide ubiquitin, and AMP-ubiquitin [goid 4221] [evidence IEA] |
| V1rc29 | vomeronasal 1 receptor, C29 (V1rc29), mRNA. | 171202 | Combining with an extracellular or intracellular messenger to initiate a change in cell activity [goid 4872] [evidence IEA]; Interacting selectively with a pheromone, a substance, or characteristic mixture of substances, that is secreted and released by an organism and detected by a second organism of the same or a closely related species, in which it causes a specific reaction, such as a definite behavioral reaction or a developmental process [goid 5550] [evidence ISS]; Combining with a pheromone to initiate a change in cell activity [goid 16503] [evidence ISS] |
| Wars2 | tryptophanyl tRNA synthetase 2 (mitochondrial) (Wars2), mRNA. | 70560 | Catalysis of the formation of aminoacyl-tRNA from ATP, amino acid, and tRNA with the release of pyrophosphate and AMP [goid 4812] [evidence IEA]; Interacting selectively with a nucleotide, any compound consisting of a nucleoside that is esterified with (ortho)phosphate or an oligophosphate at any hydroxyl group on the ribose or deoxyribose moiety [goid 166] [evidence IEA]; Catalysis of the ligation of two substances with concomitant breaking of a diphosphate linkage, usually in a nucleoside triphosphate. Ligase is the systematic name for any enzyme of EC class 6 [goid 16874] [evidence IEA]; Interacting selectively with ATP, adenosine 5'-triphosphate, a universally important coenzyme and enzyme regulator [goid 5524] [evidence IEA]; Catalysis of the reaction: ATP + L-tryptophan + tRNA(Trp) = AMP + diphosphate + L-tryptophanyl-tRNA(Trp) [goid 4830] [evidence IEA] |
| Wbp2 | WW domain binding protein 2 (Wbp2), mRNA. | 22378 | Interacting selectively with any protein or protein complex (a complex of two or more proteins that may include other nonprotein molecules) [goid 5515] [evidence IPI] |
| Wbp7 | WW domain binding protein 7 (Wbp7), mRNA. | 75410 | Catalysis of the transfer of a group, e.g. a methyl group, glycosyl group, acyl group, phosphorus-containing, or other groups, from one compound (generally regarded as the donor) to another compound (generally regarded as the acceptor). Transferase is the systematic name for any enzyme of EC class 2 [goid 16740] [evidence IEA]; Interacting selectively with zinc (Zn) ions [goid 8270] [evidence IEA]; Interacting selectively with DNA (deoxyribonucleic acid) [goid 3677] [evidence IEA]; Catalysis of the transfer of a methyl group to an acceptor molecule [goid 8168] [evidence IEA]; Interacting selectively with any metal ion [goid 46872] [evidence IEA]; Interacting selectively with any protein or protein complex (a complex of two or more proteins that may include other nonprotein molecules) [goid 5515] [evidence IEA]; Catalysis of the reaction: S-adenosyl-L-methionine + histone L-lysine = S-adenosyl-L-homocysteine + histone N6-methyl-L-lysine. The methylation of peptidyl-lysine in histones forms N6-methyl-L-lysine, N6,N6-dimethyl-L-lysine and N6,N6,N6-trimethyl-L-lysine derivatives [goid 18024] [evidence IEA] |
| Wdr13 | WD repeat domain 13 (Wdr13), mRNA. | 73447 |  |
| Wdr59 | WD repeat domain 59 (Wdr59), mRNA. | 319481 |  |
| Wfdc12 | WAP four-disulfide core domain 12 (Wfdc12), mRNA. | 192200 | Stops, prevents or reduces the activity of an endopeptidase, any enzyme that hydrolyzes nonterminal peptide bonds in polypeptides [goid 4866] [evidence IEA]; Stops, prevents or reduces the activity of serine-type endopeptidases, enzymes that catalyze the hydrolysis of nonterminal peptide bonds in a polypeptide chain; a serine residue (and a histidine residue) are at the active center of the enzyme [goid 4867] [evidence IEA]; Stops, prevents or reduces the activity of a protease, any enzyme catalyzes the hydrolysis peptide bonds [goid 30414] [evidence IEA] |
| Vmn2r81 | vomeronasal 2, receptor 81 (Vmn2r81), mRNA. | 216144 | A receptor that binds an extracellular ligand and transmits the signal to a heterotrimeric G-protein complex. These receptors are characteristically seven-transmembrane receptors and are made up of hetero- or homodimers [goid 4930] [evidence IEA]; Combining with an extracellular or intracellular messenger to initiate a change in cell activity [goid 4872] [evidence IEA]; Mediates the transfer of a signal from the outside to the inside of a cell by means other than the introduction of the signal molecule itself into the cell [goid 4871] [evidence IEA] |
| Wnt7b | wingless-related MMTV integration site 7B (Wnt7b), mRNA. | 22422 | Mediates the transfer of a signal from the outside to the inside of a cell by means other than the introduction of the signal molecule itself into the cell [goid 4871] [evidence IEA]; Interacting selectively with any protein or protein complex (a complex of two or more proteins that may include other nonprotein molecules) [goid 5515] [evidence IPI]; Interacting selectively with one or more specific sites on a receptor molecule, a macromolecule that undergoes combination with a hormone, neurotransmitter, drug or intracellular messenger to initiate a change in cell function [goid 5102] [evidence TAS] |
| Vrk2 | vaccinia related kinase 2 (Vrk2), mRNA. | 69922 | Catalysis of the reaction: ATP + a protein serine/threonine = ADP + protein serine/threonine phosphate [goid 4674] [evidence IDA]; Interacting selectively with ATP, adenosine 5'-triphosphate, a universally important coenzyme and enzyme regulator [goid 5524] [evidence IEA]; Catalysis of the phosphorylation of an amino acid residue in a protein, usually according to the reaction: a protein + ATP = a phosphoprotein + ADP [goid 4672] [evidence IEA]; Catalysis of the transfer of a phosphate group, usually from ATP, to a substrate molecule [goid 16301] [evidence IEA]; Interacting selectively with a nucleotide, any compound consisting of a nucleoside that is esterified with (ortho)phosphate or an oligophosphate at any hydroxyl group on the ribose or deoxyribose moiety [goid 166] [evidence IEA]; Catalysis of the transfer of a group, e.g. a methyl group, glycosyl group, acyl group, phosphorus-containing, or other groups, from one compound (generally regarded as the donor) to another compound (generally regarded as the acceptor). Transferase is the systematic name for any enzyme of EC class 2 [goid 16740] [evidence IEA] |
| Xpnpep1 | X-prolyl aminopeptidase (aminopeptidase P) 1, soluble (Xpnpep1), mRNA. | 170750 | Catalysis of the hydrolysis of N-terminal amino acid residues from in a polypeptide chain [goid 4177] [evidence IEA]; Catalysis of the hydrolysis of various bonds, e.g. C-O, C-N, C-C, phosphoric anhydride bonds, etc. Hydrolase is the systematic name for any enzyme of EC class 3 [goid 16787] [evidence IEA]; Interacting selectively with manganese (Mn) ions [goid 30145] [evidence IEA]; Catalysis of the hydrolysis of a peptide bond. A peptide bond is a covalent bond formed when the carbon atom from the carboxyl group of one amino acid shares electrons with the nitrogen atom from the amino group of a second amino acid [goid 8233] [evidence IEA]; Interacting selectively with any metal ion [goid 46872] [evidence IEA]; Catalysis of the hydrolysis of peptide bonds by a mechanism in which water acts as a nucleophile, one or two metal ions hold the water molecule in place, and charged amino acid side chains are ligands for the metal ions [goid 8237] [evidence IEA]; Catalysis of the hydrolysis of a peptide bond not more than three residues from the N- or C-terminus of a polypeptide chain by a mechanism in which water acts as a nucleophile, one or two metal ions hold the water molecule in place, and charged amino acid side chains are ligands for the metal ions [goid 8235] [evidence IEA] |
| Xpo7 | exportin 7 (Xpo7), mRNA. | 65246 | Enables the directed movement of proteins into, out of, within or between cells [goid 8565] [evidence IEA]; Interacting selectively with Ran, a conserved Ras-like GTP-binding protein, implicated in nucleocytoplasmic transport, cell cycle progression, spindle assembly, nuclear organization and nuclear envelope (NE) assembly [goid 8536] [evidence TAS] |
| Yif1b | Yip1 interacting factor homolog B (S. cerevisiae) (Yif1b), mRNA. | 77254 |  |
| Ywhae | tyrosine 3-monooxygenase/tryptophan 5-monooxygenase activation protein, epsilon polypeptide (Ywhae), mRNA. | 22627 | Catalysis of the incorporation of one atom from molecular oxygen into a compound and the reduction of the other atom of oxygen to water [goid 4497] [evidence IEA]; Interacting selectively with any protein or protein complex (a complex of two or more proteins that may include other nonprotein molecules) [goid 5515] [evidence IPI]; Interacting selectively with a specific domain of a protein [goid 19904] [evidence IDA] |
| Zbtb2 | zinc finger and BTB domain containing 2 (Zbtb2), mRNA. | 381990 | Interacting selectively with zinc (Zn) ions [goid 8270] [evidence IEA] |
| Zdhhc4 | zinc finger, DHHC domain containing 4 (Zdhhc4), mRNA. | 72881 | Catalysis of the generalized reaction: acyl-carrier + reactant = acyl-reactant + carrier [goid 8415] [evidence IEA]; Catalysis of the transfer of a group, e.g. a methyl group, glycosyl group, acyl group, phosphorus-containing, or other groups, from one compound (generally regarded as the donor) to another compound (generally regarded as the acceptor). Transferase is the systematic name for any enzyme of EC class 2 [goid 16740] [evidence IEA]; Interacting selectively with zinc (Zn) ions [goid 8270] [evidence IEA]; Interacting selectively with any metal ion [goid 46872] [evidence IEA] |
| Zfp238 | zinc finger protein 238 (Zfp238), transcript variant 2, mRNA. | 30928 | Interacting selectively with zinc (Zn) ions [goid 8270] [evidence IEA]; Interacting selectively with DNA (deoxyribonucleic acid) [goid 3677] [evidence IEA]; Interacting selectively with any metal ion [goid 46872] [evidence IEA]; Interacting selectively with any nucleic acid [goid 3676] [evidence IEA]; Interacting selectively with any protein or protein complex (a complex of two or more proteins that may include other nonprotein molecules) [goid 5515] [evidence IEA] |
| Zfp352 | zinc finger protein 352 (Zfp352), mRNA. | 236537 |  |
| Zfp597 | zinc finger protein 597 (Zfp597), mRNA. | 71063 | Elemental activities, such as catalysis or binding, describing the actions of a gene product at the molecular level. A given gene product may exhibit one or more molecular functions [goid 3674] [evidence ND ] |
| Zfp622 | zinc finger protein 622 (Zfp622), mRNA. | 52521 | Interacting selectively with any metal ion [goid 46872] [evidence IEA]; Interacting selectively with zinc (Zn) ions [goid 8270] [evidence IEA]; Interacting selectively with any nucleic acid [goid 3676] [evidence IEA] |
| Zxda | zinc finger, X-linked, duplicated A (Zxda), non-coding RNA. | 668171 |  |
| Zzz3 | zinc finger, ZZ domain containing 3 (Zzz3), transcript variant 1, mRNA. | 108946 | Interacting selectively with zinc (Zn) ions [goid 8270] [evidence IEA]; Interacting selectively with DNA (deoxyribonucleic acid) [goid 3677] [evidence IEA]; Interacting selectively with any metal ion [goid 46872] [evidence IEA]; Elemental activities, such as catalysis or binding, describing the actions of a gene product at the molecular level. A given gene product may exhibit one or more molecular functions [goid 3674] [evidence ND ] |
