# Supplementary material for: Cardiomyocyte Microvesicles Contain DNA/RNA and Convey Biological Messages to Target Cells
Source: PLoS One. 2012 Apr 10;7(4):e34653. doi: 10.1371/journal.pone.0034653 (PMC3323564; doi:10.1371/journal.pone.0034653)
Supplement: Table S2 — Interactions between genes/proteins coded by exosomal mRNA. Total RNA was extracted from the microvesicles/exosomes. Genes/proteins (for which identified mRNAs are encoding) were used in the bioinformatic data base and a biological network was drawn. The microvesicles/exosomes contained 1595 detected mRNA of which 1520 also were detected in cardiomyocytes. Out of these 1520 detected mRNAs in the microvesicles/exosomes, 423 could be directly connected to a biological network without addition of any extra genes/proteins. (DOC) [file pone.0034653.s005.doc]

Supplemental Table 2

| **Interactions between genes/proteins coded by exosomal mRNA** | | | |
| --- | --- | --- | --- |
| **From** | **To** | **Effect** | **Mechanism** |
| PTP4A2 | GGTase-II | inhibition | Binding |
| SCRIB | LLGL1 | activation | Binding |
| RNF6 | Ubiquitin | activation | Binding |
| Aurora-A | Histone H3 | Unspecified | Phosphorylation |
| HSF1 | LDHA | activation | Transcription regulation |
| Oct-1 | GPX3 | inhibition | Transcription regulation |
| Ubiquitin | DJ-1 | inhibition | Binding |
| GSK3 beta | CSDA | activation | Phosphorylation |
| H-Ras | p21 | activation | Covalent modification |
| DAP3 | FADD | activation | Binding |
| AKAP350 | PRKAR1A | activation | Binding |
| NEDD4L | UBADC1 | Unspecified | Ubiquitination |
| STAT3 | MAP2K5 (MEK5) | activation | Transcription regulation |
| MAPKAPK2 | Vimentin | Unspecified | Phosphorylation |
| Oct-1 | Prosaposin | Unspecified | Transcription regulation |
| XBP1 | RalB | Unspecified | Transcription regulation |
| PBK | Histone H3 | Unspecified | Phosphorylation |
| Casein kinase I delta | NDPK B | Unspecified | Phosphorylation |
| H-Ras | PI3K reg class IA (p85-alpha) | activation | Binding |
| ERR1 | Mitofusin 2 | activation | Transcription regulation |
| PTBP1 | BAG-1 | activation | Binding |
| SNRPD2 (SMD2) | SNRPD1 (SMD1) | activation | Binding |
| DMAP1 | DAXX | activation | Binding |
| HSF1 | SPOP | inhibition | Transcription regulation |
| Ubiquitin | MDM2 | inhibition | Binding |
| FTase | H-Ras | activation | Covalent modification |
| Ubiquitin | Beta-fodrin | inhibition | Binding |
| PARP-1 | HSF1 | inhibition | Covalent modification |
| PP1-cat alpha | IP3R1 | inhibition | Dephosphorylation |
| Desmin | Vimentin | inhibition | Binding |
| GAB1 | SHP-2 | activation | Binding |
| NRF1 | UQCRH | Unspecified | Transcription regulation |
| GGTase-II | Rab-27A | activation | Covalent modification |
| IRF1 | BPAG1 | inhibition | Transcription regulation |
| Ubiquitin | TXNIP (VDUP1) | inhibition | Binding |
| Oct-1 | S100A1 | Unspecified | Transcription regulation |
| TCF7L1 (TCF3) | HES6 | Unspecified | Transcription regulation |
| RRP1B (KIAA0179) | Caspase-7 | activation | co-regulation of transcription |
| HP1 alpha | p21 | Unspecified | co-regulation of transcription |
| ERK2 (MAPK1) | IP3R1 | inhibition | Phosphorylation |
| ERR1 | ATP5C | Unspecified | Transcription regulation |
| Aurora-A | NUDEL | activation | Phosphorylation |
| IRF1 | MMS21 | Unspecified | Transcription regulation |
| TCF7L1 (TCF3) | XBP1 | Unspecified | Transcription regulation |
| HES6 | p21 | activation | Transcription regulation |
| USP16 | Histone H2 | Unspecified | Deubiquitination |
| NRF1 | NDUFA10 | Unspecified | Transcription regulation |
| Ubiquitin | IRF1 | inhibition | Binding |
| STAT3 | IRF1 | activation | Transcription regulation |
| ERR1 | DAP3 | Unspecified | Transcription regulation |
| PKC-zeta | EAR2 | Unspecified | Phosphorylation |
| KLF5 | ILK | activation | Transcription regulation |
| ERK2 (MAPK1) | PARP-1 | activation | Phosphorylation |
| NRF1 | NDUFA4 | Unspecified | Transcription regulation |
| ERR1 | NDUFA4 | Unspecified | Transcription regulation |
| Ubiquitin | SCRIB | inhibition | Binding |
| Caspase-7 | FKBP4 | Unspecified | Cleavage |
| Oct-1 | Frataxin | Unspecified | Transcription regulation |
| Calmodulin | RalB | activation | Binding |
| Tubulin beta 1 | Tubulin (in microtubules) | Unspecified | Group Relation |
| BRM | Osteonectin | Unspecified | co-regulation of transcription |
| Ubiquitin | Cyclin G1 | inhibition | Binding |
| Ubiquitin | Myocardin | inhibition | Binding |
| NRF1 | ATP5G1 | Unspecified | Transcription regulation |
| GSK3 beta | Aurora-A | inhibition | Phosphorylation |
| HIST1H2BG | Histone H2B | Unspecified | Group Relation |
| NRF1 | CD47 | activation | Transcription regulation |
| UBCH7 | Ubiquitin | activation | Binding |
| OSTM1 (grey-lethal) | Ubiquitin | activation | Binding |
| RNPC1 | p21 | activation | Binding |
| NRF1 | NDUFA6 | Unspecified | Transcription regulation |
| LRSAM1 | Ubiquitin | activation | Binding |
| Caspase-7 | PKC-zeta | Unspecified | Cleavage |
| CRP3 (MLP) | ANP | activation | co-regulation of transcription |
| XBP1 | SAR1A | Unspecified | Transcription regulation |
| HCCA3 | DSCR2 | activation | Binding |
| ERK2 (MAPK1) | CDC25C | activation | Phosphorylation |
| SHP-2 | GAB1 | Unspecified | Dephosphorylation |
| GAB1 | PI3K reg class IA (p85-alpha) | activation | Binding |
| eEF1A1 | DARS | activation | Binding |
| ERR1 | ATPK | Unspecified | Transcription regulation |
| ING4 | RPL36AL | Unspecified | co-regulation of transcription |
| Rac1 | HSF1 | activation | Binding |
| MNK1 | eIF4G1 | activation | Phosphorylation |
| ULK1 | APG13 | activation | Phosphorylation |
| Oct-1 | ANP | Unspecified | Transcription regulation |
| p21 | TXNIP (VDUP1) | inhibition | co-regulation of transcription |
| LMTK2 | Histone H2B | Unspecified | Phosphorylation |
| NRF1 | MRPS11 | Unspecified | Transcription regulation |
| ERK2 (MAPK1) | IEX1 | activation | Phosphorylation |
| GGTF-II-beta | GGTase-II | Unspecified | Complex Subunit |
| UBE1 | Ubiquitin | activation | Binding |
| BART1 | STAT3 | activation | Binding |
| ACTB | NRAMP1 | activation | co-regulation of transcription |
| NRF1 | UQCRC1 | Unspecified | Transcription regulation |
| Trp53inp2 | MAP1LC3A | activation | Binding |
| SUMO-1 | GSK3 beta | activation | Binding |
| STAT3 | Beta-fodrin | Unspecified | Transcription regulation |
| Ubiquitin | CLIM1 | inhibition | Binding |
| Ubiquitin | MEK4(MAP2K4) | inhibition | Binding |
| UHRF2 | Ubiquitin | activation | Binding |
| PKC-zeta | AKT3 | inhibition | Binding |
| Oct-1 | Histone H2B | activation | Transcription regulation |
| DAXX | HSF1 | activation | Binding |
| UFD2 | Ubiquitin | activation | Binding |
| PTBP1 | CBF beta | activation | Binding |
| ERR1 | REDD1 | Unspecified | Transcription regulation |
| mTOR | 4E-BP2 | inhibition | Phosphorylation |
| Myocardin | Myocardin | activation | Binding |
| NDPK A | MIF | inhibition | Binding |
| Ubiquitin | SREBP2 precursor | inhibition | Binding |
| STAT3 | p21 | activation | Transcription regulation |
| Ubiquitin | Bim | inhibition | Binding |
| RPL23 | MDM2 | inhibition | Binding |
| Ubiquitin | GSK3 beta | inhibition | Binding |
| PP2C beta | MKK7 (MAP2K7) | inhibition | Dephosphorylation |
| Caspase-7 | Vimentin | inhibition | Cleavage |
| N-CoR | p21 | inhibition | co-regulation of transcription |
| Smac/Diablo | Smac/Diablo | activation | Binding |
| Vimentin | SCRIB | activation | Binding |
| TLK1 | TLK1 | Unspecified | Phosphorylation |
| Rac1 | PKC-zeta | activation | Binding |
| PKC-zeta | PRKCSH | activation | Binding |
| NRF1 | Metallothionein-I | activation | Transcription regulation |
| PP2C alpha | MEK3(MAP2K3) | inhibition | Dephosphorylation |
| Frataxin | ACON | activation | Binding |
| PKC-zeta | HSF1 | inhibition | Phosphorylation |
| FTase | Lamin A/C | Unspecified | Covalent modification |
| REDD1 | Tuberin | activation | Unspecified |
| Adducin | Calmodulin | inhibition | Binding |
| ING4 | p21 | activation | Unspecified |
| Ubiquitin | Vimentin | inhibition | Binding |
| Aurora-A | CENP-A | activation | Phosphorylation |
| APLP2 precursor | APLP2 active fragment | Unspecified | Group Relation |
| Caspase-7 | P4HB | Unspecified | Cleavage |
| ERR1 | NDUFA3 | Unspecified | Transcription regulation |
| Caspase-7 | PARP-1 | inhibition | Cleavage |
| HIPK2 | MDM2 | inhibition | Phosphorylation |
| GSK3 beta | Myocardin | inhibition | Phosphorylation |
| SUMO-1 | IRF1 | inhibition | Binding |
| CaMKK2 | CaMKK2 | activation | Phosphorylation |
| Kid | Tubulin (in microtubules) | activation | Binding |
| STAT3 | CISH | activation | Transcription regulation |
| CDK8 | p21 | activation | co-regulation of transcription |
| ERR1 | ATP5G1 | activation | Transcription regulation |
| FHL2 | HIPK2 | activation | Binding |
| Ubiquitin | Smac/Diablo | inhibition | Binding |
| GSK3 beta | p21 | inhibition | Phosphorylation |
| PARD6A | PKC-zeta | activation | Binding |
| PKC-zeta | ERK2 (MAPK1) | activation | Binding |
| Oct-1 | MRG15 | Unspecified | Transcription regulation |
| RD protein | Histone H2B | Unspecified | Ubiquitination |
| Ubiquitin | IP3R1 | inhibition | Binding |
| eIF4G1 | MNK2(GPRK7) | activation | Binding |
| STAT3 | KLF5 | activation | Transcription regulation |
| NRF1 | NDUFA5 | activation | Transcription regulation |
| PLAGL2 | LDHA | activation | Transcription regulation |
| PP1-cat alpha | Vimentin | Unspecified | Dephosphorylation |
| S100A1 | Desmin | inhibition | Binding |
| NRF1 | ATP5J | Unspecified | Transcription regulation |
| MAFbx | Cardiac MyBP-C | inhibition | Ubiquitination |
| Caspase-7 | PRDX2 | Unspecified | Cleavage |
| LKB1 | NUAK2 | activation | Phosphorylation |
| IRF1 | GSTP1 | Unspecified | Transcription regulation |
| Gamma adducin | Adducin | Unspecified | Group Relation |
| PKC-zeta | MEK4(MAP2K4) | activation | Binding |
| MMS21 | SUMO-1 | activation | Binding |
| PRA1 | H-Ras | activation | Binding |
| FADD | Acid sphingomyelinase | activation | Unspecified |
| p115 | MIF | activation | Binding |
| HSF1 | MAK10 | activation | Transcription regulation |
| Ubiquitin | HA95 | inhibition | Binding |
| MAPKAPK2 | ARPC5 | Unspecified | Phosphorylation |
| ERK2 (MAPK1) | HSF1 | inhibition | Phosphorylation |
| YKT6 | GS15 | activation | Complex formation |
| NEDD4L | Ubiquitin | activation | Binding |
| STAT3 | MyD88 | activation | Transcription regulation |
| RNF8 | Ubiquitin | activation | Binding |
| C/EBP zeta | Bim | activation | Transcription regulation |
| Caspase-7 | BAG-3 | inhibition | Cleavage |
| STAT3 | Zyxin | Unspecified | Transcription regulation |
| TFIIIA | Tuberin | Unspecified | Transcription regulation |
| SUMO-1 | SREBP2 (nuclear) | inhibition | Binding |
| BTG1 | CAF1 | activation | Binding |
| Rac1 | PP5 | activation | Binding |
| BCKD-kinase | BCKD-kinase | inhibition | Phosphorylation |
| TOPORS | Ubiquitin | activation | Binding |
| Ubiquitin | CD81 | inhibition | Binding |
| Ubiquitin | REDD1 | inhibition | Binding |
| IFNA6_MOUSE | STAT3 | activation | Unspecified |
| Alpha adducin | Adducin | Unspecified | Group Relation |
| C3G | Rap 1 | activation | Transformation |
| ERR1 | KCNH2 | Unspecified | Transcription regulation |
| RNF181 | Ubiquitin | activation | Binding |
| LMTK2 | PP1-cat alpha | inhibition | Phosphorylation |
| NRF1 | COX VIIa-2L | Unspecified | Transcription regulation |
| SFRS2 (SC-35) | PDHA (somatic) | activation | Binding |
| ERR1 | UQCRC2 | Unspecified | Transcription regulation |
| XBP1 | C/EBP zeta | Unspecified | Transcription regulation |
| p120GAP | H-Ras | inhibition | Transformation |
| PP2C beta | MEK3(MAP2K3) | inhibition | Dephosphorylation |
| Myocardin | ACTA2 | activation | co-regulation of transcription |
| G9a | p21 | inhibition | co-regulation of transcription |
| HSF1 | DNAJB6 (Hdj-1) | activation | Transcription regulation |
| ICMT | N-Ras | activation | Covalent modification |
| mTOR | STAT3 | activation | Phosphorylation |
| ERR1 | CDC20 | Unspecified | Transcription regulation |
| Gas6 | STAT3 | activation | Unspecified |
| NUDEL | Dynein 1, cytoplasmic, heavy chain | activation | Binding |
| NEDD4L | AKT3 | Unspecified | Ubiquitination |
| DNAJC3 | HSC70 | activation | Binding |
| Rac1 | STAT3 | activation | Binding |
| NRF1 | ATPAF2 | Unspecified | Transcription regulation |
| NDPK B | ERK2 (MAPK1) | Unspecified | Transcription regulation |
| Bim | Bcl-W | inhibition | Binding |
| SLC31A1 | SLC31A1 | activation | Binding |
| Oct-1 | IEX1 | Unspecified | Transcription regulation |
| mTOR | BCKD-kinase | activation | Unspecified |
| NRF1 | Dynein 1, cytoplasmic, heavy chain | Unspecified | Transcription regulation |
| NMP200 | Ubiquitin | activation | Binding |
| XBP1 | MARCKS | Unspecified | Transcription regulation |
| Tubulin (in microtubules) | HSP90 beta | activation | Binding |
| MyD88 | IRF1 | activation | Binding |
| Caspase-7 | COPS6 | inhibition | Cleavage |
| DDX17 | MDM2 | activation | co-regulation of transcription |
| NRF1 | DAP3 | Unspecified | Transcription regulation |
| UBE1 | E2N(UBC13) | activation | Binding |
| TFIIIA | GM3 synthase | Unspecified | Transcription regulation |
| NRF1 | NDUFB6 | Unspecified | Transcription regulation |
| XBP1 | Vimentin | Unspecified | Transcription regulation |
| mtNOS1 | DAP3 | activation | Binding |
| NRF1 | p115 | Unspecified | Transcription regulation |
| PIAS2 | DJ-1 | activation | Sumoylation |
| TFIIIA | LPL | Unspecified | Transcription regulation |
| SUZ12 | Casein kinase I gamma 2 | Unspecified | co-regulation of transcription |
| IRF1 | RYK | Unspecified | Transcription regulation |
| G9a | Survivin | inhibition | co-regulation of transcription |
| GCP16 | H-Ras | activation | Covalent modification |
| STAT3 | Perforin | activation | Transcription regulation |
| Profilin I | ACTB | activation | Binding |
| Ubiquitin | COPS6 | inhibition | Binding |
| TIF1-beta | 6PGL | Unspecified | co-regulation of transcription |
| PARP-1 | ERK2 (MAPK1) | activation | Binding |
| MM-1 | Tubulin alpha-1B | activation | Binding |
| Saposin C | Acid sphingomyelinase | activation | Binding |
| HP1 alpha | CDC25C | Unspecified | co-regulation of transcription |
| MDM2 | Cyclin G1 | inhibition | Ubiquitination |
| HJURP | CENP-A | activation | Binding |
| Rac1 | PARD6A | activation | Binding |
| HIPK2 | DAXX | activation | Phosphorylation |
| HSC70 | Bim | activation | Binding |
| NRF1 | ATP5C | Unspecified | Transcription regulation |
| EDF1 | LXR-alpha | activation | Binding |
| Ubiquitin | APP-BP1 | inhibition | Binding |
| TOLLIP | SUMO-1 | activation | Binding |
| UCHL1 | Ubiquitin | activation | Cleavage |
| TOPORS | TIF1-beta | Unspecified | Sumoylation |
| Survivin | Caspase-7 | inhibition | Binding |
| Myocardin | p21 | activation | co-regulation of transcription |
| NEDD4L | Hrs | Unspecified | Ubiquitination |
| Saposin D | Acid sphingomyelinase | activation | Binding |
| Histone H3.3 | Histone H3 | Unspecified | Group Relation |
| SUMO-1 | MDM2 | activation | Binding |
| NRF1 | E2F6 | activation | Transcription regulation |
| mTOR | ULK1 | inhibition | Phosphorylation |
| SUMO-1 | ZXDC | activation | Binding |
| XBP1 | Dymeclin | Unspecified | Transcription regulation |
| VRK1 | Histone H2B | Unspecified | Phosphorylation |
| DORFIN | Ubiquitin | activation | Binding |
| ERR1 | SLC12A6 | Unspecified | Transcription regulation |
| CAPZ beta | Alpha-actinin 2 | activation | Binding |
| DJ-1 | PIAS2 | inhibition | Binding |
| ING4 | RPS2 | Unspecified | co-regulation of transcription |
| KLF5 | PARP-1 | inhibition | Binding |
| BAG-3 | HSP20 | activation | Binding |
| MDM2 | RPS7 | Unspecified | Ubiquitination |
| ING4 | MAT2A | Unspecified | co-regulation of transcription |
| SUZ12 | Histone H3.3 | Unspecified | co-regulation of transcription |
| ERR1 | COX10 | Unspecified | Transcription regulation |
| HSF1 | KCTD20 | activation | Transcription regulation |
| Ubiquitin | p21 | inhibition | Binding |
| Ubiquitin | CD151 | inhibition | Binding |
| Casein kinase I delta | ARFGAP1 | activation | Phosphorylation |
| IRF1 | p21 | activation | Transcription regulation |
| Ubiquitin | NRAMP2 | inhibition | Binding |
| MNK1 | MNK1 | inhibition | Unspecified |
| Ubiquitin C | Ubiquitin | Unspecified | Group Relation |
| Ubiquitin | Ubiquitin C | Unspecified | Group Relation |
| XBP1 | DNAJC3 | activation | Transcription regulation |
| LIMK2 | Histone H2 | Unspecified | Phosphorylation |
| TOPORS | eEF1A1 | Unspecified | Sumoylation |
| TLK1 | Histone H3 | Unspecified | Phosphorylation |
| Ubiquitin | FRS2 | inhibition | Binding |
| Oct-1 | Ca-ATPase2 | Unspecified | Transcription regulation |
| Mitofusin 2 | H-Ras | inhibition | Binding |
| PERC | Mitofusin 2 | activation | co-regulation of transcription |
| NRF1 | TOM34 | activation | Transcription regulation |
| ERR1 | IRF1 | activation | Transcription regulation |
| Caspase-7 | NDPK B | Unspecified | Cleavage |
| KLF5 | p21 | inhibition | Transcription regulation |
| FHL2 | SMN1 | inhibition | co-regulation of transcription |
| Oct-1 | FBXL10 | Unspecified | Transcription regulation |
| RPS7 | MDM2 | inhibition | Binding |
| ERR1 | NDUFC1 | Unspecified | Transcription regulation |
| Caspase-7 | Apaf-1 | inhibition | Cleavage |
| PP2C alpha | MKK7 (MAP2K7) | inhibition | Dephosphorylation |
| NRF1 | COX17 | activation | Transcription regulation |
| Rnf135 | Ubiquitin | activation | Binding |
| MDM2 | Histone H2B | inhibition | Ubiquitination |
| ING4 | EGLN1 | activation | Binding |
| NRF1 | DAP13 | Unspecified | Transcription regulation |
| ERR1 | ATP5F1 | Unspecified | Transcription regulation |
| SREBP2 (nuclear) | LPL | Unspecified | Transcription regulation |
| CDC25C | H-Ras | activation | Transformation |
| STAT3 | MRPS34 | Unspecified | Transcription regulation |
| FKBP4 | Tubulin (in microtubules) | activation | Binding |
| ERR1 | TIMM8B | Unspecified | Transcription regulation |
| NRF1 | HP1 alpha | activation | Transcription regulation |
| IRF1 | SMN1 | activation | Transcription regulation |
| E2F6 | ALG8 | Unspecified | Transcription regulation |
| STAT3 | STAT3 | activation | Binding |
| PRMT3 | RPS2 | Unspecified | Covalent modification |
| SREBP2 (nuclear) | ERG1 | activation | Transcription regulation |
| PRAS40 | mTOR | inhibition | Binding |
| AMFR | VCP | activation | Binding |
| Ubiquitin | HIPK2 | inhibition | Binding |
| PSMA7 | XBP1 | inhibition | Binding |
| SREBP2 (nuclear) | SREBP2 precursor | activation | Transcription regulation |
| POLD reg (p12) | BLM | activation | Binding |
| LIMD1 | PKC-zeta | activation | Binding |
| LKB1 | MARK2 | activation | Phosphorylation |
| Ubiquitin | TMP21 | inhibition | Binding |
| hnRNP L | DJ-1 | activation | co-regulation of transcription |
| FBXL10 | Histone H3 | Unspecified | Covalent modification |
| SHP-2 | SHP-2 | Unspecified | Transport |
| ERR1 | FAM102A | Unspecified | Transcription regulation |
| GSK3 beta | HSF1 | inhibition | Phosphorylation |
| TTC3 | AKT3 | inhibition | Ubiquitination |
| PKC-zeta | FADD | inhibition | Phosphorylation |
| HSF1 | RPS14 | activation | Transcription regulation |
| PP2C beta | MEK4(MAP2K4) | inhibition | Dephosphorylation |
| Ubiquitin | GUCY1A3 | inhibition | Binding |
| HSF1 | TXNIP (VDUP1) | activation | Transcription regulation |
| ERR1 | UQCRC1 | Unspecified | Transcription regulation |
| FHL2 | Myocardin | activation | Binding |
| LIMK2 | Histone H3 | Unspecified | Phosphorylation |
| PKC-zeta | LKB1 | activation | Phosphorylation |
| MDM2 | BRD2 | Unspecified | co-regulation of transcription |
| G3P1 | Gapdh | Unspecified | Group Relation |
| MAPKAPK2 | ZFP36(Tristetraprolin) | inhibition | Phosphorylation |
| PI3K reg class IA (p85-alpha) | PKC-zeta | activation | Binding |
| ERR1 | NDUFB8 | Unspecified | Transcription regulation |
| ERK2 (MAPK1) | CSDA | Unspecified | Phosphorylation |
| TCF7L1 (TCF3) | TIF1-beta | Unspecified | Transcription regulation |
| MIF | GFER | activation | Binding |
| PKC-zeta | FLRE | activation | Phosphorylation |
| VRK1 | Histone H3 | Unspecified | Phosphorylation |
| BRM | ALPL | Unspecified | co-regulation of transcription |
| Importin 13 | Mago nashi | Unspecified | Transport |
| Oct-1 | COX VIIa-2L | Unspecified | Transcription regulation |
| HSF1 | MRPL2 | inhibition | Transcription regulation |
| SUMO-1 | HSF1 | activation | Binding |
| P52rIPK | DNAJC3 | inhibition | Binding |
| HSF1 | ACON | Unspecified | Transcription regulation |
| MAPKAPK2 | HSF1 | inhibition | Phosphorylation |
| HSF1 | NRF1 | inhibition | Transcription regulation |
| DAPK2 | DAPK2 | inhibition | Phosphorylation |
| HSF1 | GBAS | activation | Transcription regulation |
| PTBP1 | Cyclin T1 | activation | Binding |
| RIP | Vimentin | Unspecified | Transport |
| Oct-1 | GUCY1A3 | Unspecified | Transcription regulation |
| Tuberin | mTOR | inhibition | Binding |
| XBP1 | UVRAG | Unspecified | Transcription regulation |
| PIAS2 | SUMO-1 | activation | Binding |
| NFIX | LXR-alpha | inhibition | Transcription regulation |
| Ubiquitin | FHL2 | inhibition | Binding |
| ERK2 (MAPK1) | Tuberin | inhibition | Phosphorylation |
| ERR1 | ATP1B1 | activation | Transcription regulation |
| TOPORS | hnRNP L | Unspecified | Sumoylation |
| IRF1 | FCGRT | Unspecified | Transcription regulation |
| DNAJB6 (Hdj-1) | HSC70 | activation | Binding |
| HSF1 | TPR | activation | Binding |
| PIAS2 | SUZ12 | Unspecified | Sumoylation |
| ZFP36(Tristetraprolin) | MLLT11 | inhibition | Binding |
| E2N(UBC13) | RNF8 | activation | Binding |
| Calmodulin | MARCKS | inhibition | Binding |
| STAT3 | VAMP4 | Unspecified | Transcription regulation |
| MEK4(MAP2K4) | MEK4(MAP2K4) | activation | Phosphorylation |
| RBCK1 | Ubiquitin | activation | Binding |
| ERK3 | Rac1 | inhibition | Unspecified |
| Ubiquitin | RNF8 | inhibition | Binding |
| MIB2 | Ubiquitin | activation | Binding |
| ERR1 | CYC1 | Unspecified | Transcription regulation |
| NRF1 | COX VIa-1 | activation | Transcription regulation |
| ERR1 | NDUFS6 | Unspecified | Transcription regulation |
| HIPK2 | STAT3 | Unspecified | Phosphorylation |
| DAXX | MDM2 | activation | Binding |
| HSF1 | FKBP4 | activation | Transcription regulation |
| GSK3 beta | CTP synthase | inhibition | Phosphorylation |
| Oct-1 | BLCAP | Unspecified | Transcription regulation |
| SUMO-1 | SLIM1 | inhibition | Binding |
| Ubiquitin | DAXX | inhibition | Binding |
| SUZ12 | MAFbx | inhibition | co-regulation of transcription |
| Calmodulin | Titin | activation | Binding |
| p200RhoGAP | Rac1 | inhibition | Transformation |
| Caspase-7 | ACTA2 | Unspecified | Cleavage |
| NEDD4L | RPL30 | Unspecified | Ubiquitination |
| eEF1G | Vimentin | activation | Unspecified |
| IRF1 | TfR1 | Unspecified | Transcription regulation |
| TAZ | TEF-4 | activation | Binding |
| Tuberin | Rac1 | activation | Unspecified |
| CDC20 | Thymidylate kinase | inhibition | Binding |
| Calmodulin | Rac1 | activation | Binding |
| Apaf-1 | FEM1B | activation | Binding |
| NEDD4L | ZADH2 | Unspecified | Ubiquitination |
| NRF1 | NDUFB8 | Unspecified | Transcription regulation |
| Rac1 | PRK2 | activation | Binding |
| FRS2 | SHP-2 | activation | Binding |
| TRAF3 | Smac/Diablo | activation | Binding |
| H-Ras | RIN2 | activation | Binding |
| UBE3C | Ubiquitin | activation | Binding |
| MDM2 | DBI | activation | co-regulation of transcription |
| PKC-zeta | MAP2K5 (MEK5) | activation | Binding |
| E2F6 | AFG3L1 | Unspecified | Transcription regulation |
| GAB1 | MAP2K5 (MEK5) | activation | Unspecified |
| MyD88 | MyD88 | activation | Binding |
| Ubiquitin | IDE | inhibition | Binding |
| Ubiquitin | AKT3 | inhibition | Binding |
| TOB2 | CAF1 | activation | Binding |
| CRMP2 | Tubulin (in microtubules) | activation | Binding |
| XBP1 | UFC1 | Unspecified | Transcription regulation |
| Ubiquitin | LXR-alpha | inhibition | Binding |
| ZBTB2 | p21 | inhibition | Transcription regulation |
| Tubulin alpha-1B | Tubulin (in microtubules) | Unspecified | Group Relation |
| MEK2(MAP2K2) | ERK2 (MAPK1) | activation | Phosphorylation |
| ERK2 (MAPK1) | ZFP36(Tristetraprolin) | Unspecified | Phosphorylation |
| Ubiquitin | MAPRPE1(EB1) | inhibition | Binding |
| C/EBP zeta | CARP | inhibition | Transcription regulation |
| ERR1 | ATP5G3 | activation | Transcription regulation |
| PRMT3 | PABP2 | activation | Covalent modification |
| E2N(UBC13) | Ubiquitin | activation | Binding |
| ERK2 (MAPK1) | STAT3 | inhibition | Phosphorylation |
| ICMT | H-Ras | Unspecified | Covalent modification |
| Ubiquitin | AMFR | inhibition | Binding |
| p21 | STAT3 | inhibition | Binding |
| EDF1 | ANP | activation | Transcription regulation |
| IRF1 | MyD88 | activation | Transcription regulation |
| GGTF-II-alpha | GGTase-II | Unspecified | Complex Subunit |
| Ubiquitin | KLF5 | inhibition | Binding |
| Ubiquitin | Tome-1 | inhibition | Binding |
| NRF1 | NDUFA9 | Unspecified | Transcription regulation |
| MAP3K3 | MEK4(MAP2K4) | activation | Phosphorylation |
| FTase-beta | FTase | Unspecified | Complex Subunit |
| ERR1 | COX VIb-1 | Unspecified | Transcription regulation |
| DAXX | USP7 | activation | Binding |
| Calmodulin | Alpha-actinin 2 | inhibition | Competition |
| ERR1 | DAP13 | Unspecified | Transcription regulation |
| PRKCSH | Glucosidase II, alpha subunits | Unspecified | Complex formation |
| E2F6 | AIP | Unspecified | Transcription regulation |
| DJ-1 | DAXX | inhibition | Binding |
| TIF1-beta | LYRM5 | Unspecified | co-regulation of transcription |
| SREBP2 (nuclear) | ABCA7 | activation | Transcription regulation |
| eIF4G1 | MNK1 | activation | Binding |
| Ubiquitin | eIF3S5 | inhibition | Binding |
| NRF1 | CYC1 | Unspecified | Transcription regulation |
| NEDD4L | NRAMP2 | inhibition | Ubiquitination |
| Histone H2B | Histone H2 | Unspecified | Group Relation |
| Ubiquitin | DDX17 | inhibition | Binding |
| Casein kinase I delta | NDPK A | activation | Phosphorylation |
| Reticulon 3 | FADD | activation | Binding |
| Oct-1 | FDPS | Unspecified | Transcription regulation |
| NRF1 | ATP5G2 | Unspecified | Transcription regulation |
| SUMO-1 | PLAGL2 | inhibition | Binding |
| ERR1 | COX VIIa-2L | Unspecified | Transcription regulation |
| ERK2 (MAPK1) | Alpha adducin | Unspecified | Phosphorylation |
| LASP1 | Zyxin | activation | Binding |
| E2F6 | Apaf-1 | inhibition | Transcription regulation |
| PP2A cat (beta) | ERK2 (MAPK1) | inhibition | Dephosphorylation |
| ERK2 (MAPK1) | p21 | inhibition | Phosphorylation |
| Calmodulin | IP3R1 | inhibition | Binding |
| Perforin | Perforin | activation | Binding |
| BRM | Rab-27A | Unspecified | co-regulation of transcription |
| RXRB | SMN1 | Unspecified | Transcription regulation |
| ING4 | Exostosin-1 | activation | co-regulation of transcription |
| Ubiquitin | Kid | inhibition | Binding |
| TIF1-beta | p21 | inhibition | co-regulation of transcription |
| ZFP36(Tristetraprolin) | B4GT3 | inhibition | Binding |
| PERC | ACADM | activation | co-regulation of transcription |
| ERR1 | ATP1A1 | Unspecified | Transcription regulation |
| YKT6 | YKT6 | inhibition | Binding |
| DAAM1 | Profilin I | activation | Binding |
| USP7 | MDM2 | activation | Deubiquitination |
| ILK | GSK3 beta | inhibition | Phosphorylation |
| PKC-zeta | KCNAB2 | activation | Phosphorylation |
| FKBP8 | mTOR | inhibition | Binding |
| VCP | DORFIN | activation | Binding |
| FKBP8 | EGLN1 | inhibition | Binding |
| GSK3 beta | CRMP2 | inhibition | Phosphorylation |
| GSK3 beta | PDHA (somatic) | inhibition | Phosphorylation |
| BLM | POLD reg (p12) | activation | Binding |
| PCGF1 | p21 | inhibition | Transcription regulation |
| MyD88 | FADD | activation | Binding |
| ETFB | ETF complex | Unspecified | Complex Subunit |
| PREP1 | Oct-1 | Unspecified | Dephosphorylation |
| SREBP2 (nuclear) | CTE2 | activation | Transcription regulation |
| RPA1 | BLM | activation | Binding |
| ERK2 (MAPK1) | SREBP2 precursor | activation | Phosphorylation |
| ATP1B1 | ATP1A1 | activation | Binding |
| ERK2 (MAPK1) | TPR | Unspecified | Phosphorylation |
| LXR-alpha | LPL | Unspecified | Transcription regulation |
| SUMO-1 | TIF1-beta | activation | Binding |
| ZBTB2 | MDM2 | activation | Transcription regulation |
| PKC-zeta | PTK9L | Unspecified | Phosphorylation |
| MAP3K3 | MAP2K5 (MEK5) | activation | Phosphorylation |
| DAXX | STAT3 | inhibition | Binding |
| UBE2E2 | Ubiquitin | activation | Binding |
| Apaf-1 | Apaf-1 | activation | Binding |
| Casein kinase I delta | MDM2 | activation | Phosphorylation |
| PABP2 | PRMT3 | inhibition | Binding |
| ICMT | Rac1 | activation | Covalent modification |
| UBXD1 | MDM2 | activation | Binding |
| PARP-1 | CENP-A | Unspecified | Covalent modification |
| TXNDC5 | PLOD2 | Unspecified | Covalent modification |
| STAT3 | HSP90 beta | activation | Transcription regulation |
| HSF1 | CDC20 | inhibition | Binding |
| GSK3 beta | Tuberin | activation | Phosphorylation |
| CENP-A | Histone H3.3 | inhibition | Competition |
| ERK2 (MAPK1) | GSK3 beta | inhibition | Phosphorylation |
| DAXX | DAXX | Unspecified | Transport |
| HSF1 | TCP1-zeta-1 | activation | Transcription regulation |
| ERR1 | ACON | activation | Transcription regulation |
| SUMO-1 | N-CoR | activation | Binding |
| mTOR | LIPIN1 | Unspecified | Phosphorylation |
| ERR1 | UQCR10 | Unspecified | Transcription regulation |
| MIF | ERK2 (MAPK1) | activation | Unspecified |
| mTOR | APG13 | inhibition | Phosphorylation |
| NADE(NGFRAP1) | Smac/Diablo | activation | Binding |
| TOPORS | SUMO-1 | activation | Binding |
| GAK | HSC70 | activation | Binding |
| IP3R1 | FKBP12 | activation | Binding |
| NRF1 | C15orf15 | Unspecified | Transcription regulation |
| UBE1 | Ubiquitin C | activation | Binding |
| N-CoR | LPL | inhibition | Transcription regulation |
| Oct-1 | LPL | Unspecified | Transcription regulation |
| CDCA8 | Survivin | activation | Binding |
| ERK2 (MAPK1) | MNK1 | Unspecified | Phosphorylation |
| VRK1 | VRK1 | Unspecified | Phosphorylation |
| MAP3K3 | MKK7 (MAP2K7) | activation | Phosphorylation |
| PIAS2 | MDM2 | activation | Sumoylation |
| Smac/Diablo | Survivin | inhibition | Binding |
| ERK2 (MAPK1) | Bim | inhibition | Phosphorylation |
| STK39 | STK39 | activation | Phosphorylation |
| eEF1A1 | HSF1 | activation | Binding |
| PKC-zeta | Cardiac MyBP-C | Unspecified | Phosphorylation |
| ETFA | ETF complex | Unspecified | Complex Subunit |
| NDPK A | NDPK A | Unspecified | Phosphorylation |
| Ubiquitin | HSP90 beta | inhibition | Binding |
| Cyclin D3 | Tuberin | inhibition | Binding |
| PERC | LXR-alpha | activation | Binding |
| G-protein alpha-o | Tubulin (in microtubules) | inhibition | Transformation |
| GSK3 beta | NACA | inhibition | Phosphorylation |
| G9a | Histone H3 | Unspecified | Covalent modification |
| Atg101 | APG13 | activation | Binding |
| ERR1 | NDUFA6 | Unspecified | Transcription regulation |
| MAPKAPK2 | Tuberin | activation | Phosphorylation |
| ActRIIB | PI3K reg class IA (p85-alpha) | activation | Binding |
| VRK3 | ERK2 (MAPK1) | inhibition | Binding |
| Ubiquitin | Tuberin | inhibition | Binding |
| p120GAP | N-Ras | inhibition | Transformation |
| GBL | mTOR | activation | Binding |
| FHL2 | ERK2 (MAPK1) | inhibition | Binding |
| Ubiquitin | H-Ras | activation | Binding |
| E2F6 | HP1 alpha | inhibition | Transcription regulation |
| SREBP2 (nuclear) | PEMT | activation | Transcription regulation |
| ERR1 | ATP5B | activation | Transcription regulation |
| E2F6 | CYC1 | inhibition | Transcription regulation |
| SUMO-1 | LIPIN1 | activation | Binding |
| NRF1 | TFB2M | activation | Transcription regulation |
| PDP2 | PDHA (somatic) | activation | Dephosphorylation |
| Oct-1 | Zimp7 | Unspecified | Transcription regulation |
| Ubiquitin | ERK3 | inhibition | Binding |
| ALPL | eEF1A1 | inhibition | Binding |
| STAT3 | PI3K reg class IA (p85-alpha) | activation | Transcription regulation |
| Miz-1 | NRAMP1 | Unspecified | Transcription regulation |
| BAG-1 | HSC70 | inhibition | Binding |
| APLP2 active fragment | APLP2 precursor | Unspecified | Group Relation |
| FHL2 | KCNH2 | activation | Binding |
| LKB1 | SIK | activation | Phosphorylation |
| HSF1 | HSP90 beta | activation | Transcription regulation |
| STAT3 | IEX1 | activation | Transcription regulation |
| IEX1 | ERK2 (MAPK1) | activation | Binding |
| NRF1 | ATP5G3 | Unspecified | Transcription regulation |
| Calmodulin | DAPK2 | activation | Binding |
| PPM1F | ILK | inhibition | Dephosphorylation |
| ERK2 (MAPK1) | DOC-1R | Unspecified | Phosphorylation |
| MAFbx | eIF3S5 | inhibition | Binding |
| TIF1-beta | MDM2 | activation | Binding |
| Aurora-A | GSK3 beta | inhibition | Phosphorylation |
| NDPK B | NDPK complex | Unspecified | Complex Subunit |
| Vimentin | ERK2 (MAPK1) | activation | Binding |
| NEDD4L | WBP-2 | Unspecified | Ubiquitination |
| BAG-3 | HSC70 | inhibition | Binding |
| Ubiquitin | Aurora-A | inhibition | Binding |
| PARP-1 | NRF1 | Unspecified | Covalent modification |
| UBXD1 | VCP | inhibition | Binding |
| GAB1 | PAK4 | activation | Binding |
| NACA | FADD | inhibition | Binding |
| MDM2 | HIPK2 | inhibition | Ubiquitination |
| SUMO-1 | Myocardin | activation | Binding |
| PKC-zeta | MARK2 | inhibition | Phosphorylation |
| CDH1 | Thymidylate kinase | inhibition | Binding |
| SPOP | DAXX | inhibition | Binding |
| PKC-zeta | GSK3 beta | inhibition | Phosphorylation |
| CDK8 | MDM2 | activation | co-regulation of transcription |
| AKAP350 | Casein kinase I delta | activation | Binding |
| Calmodulin | FKBP8 | activation | Binding |
| Caveolin-3 | PFKM | activation | Binding |
| Ubiquitin | KCNH2 | inhibition | Binding |
| ERR1 | NDUFA9 | Unspecified | Transcription regulation |
| ERR1 | ACADM | activation | Transcription regulation |
| PPP4C | SMN1 | activation | Unspecified |
| Ubiquitin | ERR1 | inhibition | Binding |
| mTOR | mTOR | activation | Phosphorylation |
| PP1-cat alpha | SLC12A6 | activation | Dephosphorylation |
| HSF1 | LIPIN1 | activation | Transcription regulation |
| NRF1 | ATPK | Unspecified | Transcription regulation |
| mTOR | PRAS40 | Unspecified | Phosphorylation |
| CD27BP | MDM2 | activation | Binding |
| E2F6 | ASCC1 | Unspecified | Transcription regulation |
| Furin | Dystroglycan | Unspecified | Cleavage |
| PP2C alpha | MEK4(MAP2K4) | inhibition | Dephosphorylation |
| USP22 | Histone H2B | Unspecified | Deubiquitination |
| HBP1 | p21 | inhibition | Transcription regulation |
| MDM2 | p21 | inhibition | Binding |
| ING4 | Rod | Unspecified | co-regulation of transcription |
| BRM | p21 | Unspecified | co-regulation of transcription |
| AMSH | GAB1 | inhibition | Competition |
| UIP5 | Ubiquitin | activation | Binding |
| Oct-1 | Oct-1 | Unspecified | Transcription regulation |
| NRF1 | PARP-1 | activation | Binding |
| SREBP2 (nuclear) | Caspase-7 | activation | Transcription regulation |
| Ubiquitin | ULK1 | inhibition | Binding |
| TCF7L1 (TCF3) | BRM | Unspecified | Transcription regulation |
| NRF1 | COX VIb-1 | activation | Transcription regulation |
| FTase-alpha | FTase | Unspecified | Complex Subunit |
| STAT3 | Vimentin | activation | Transcription regulation |
| TRIP15 | Histone H3 | inhibition | Binding |
| ATRIP | RPA1 | activation | Binding |
| MAPKAPK2 | MDM2 | activation | Phosphorylation |
| Casein kinase I delta | p115 | activation | Phosphorylation |
| Ubiquitin | BAG-1 | inhibition | Binding |
| STAT3 | FKBP8 | Unspecified | Transcription regulation |
| ERR1 | ATP5L | Unspecified | Transcription regulation |
| GSK3 beta | PKC-zeta | inhibition | Phosphorylation |
| STAT3 | HSF1 | activation | Binding |
| FBXW5 | Tuberin | inhibition | Binding |
| XBP1 | MSS4 | Unspecified | Transcription regulation |
| Csp | HSC70 | activation | Binding |
| Oct-1 | MAFbx | inhibition | Transcription regulation |
| TIF1-beta | SUV420H1 | Unspecified | co-regulation of transcription |
| MAP3K3 | MEK3(MAP2K3) | activation | Phosphorylation |
| TBX20 | ANP | inhibition | Transcription regulation |
| N-CoR | p18 | inhibition | co-regulation of transcription |
| HSF1 | BAG-3 | activation | Transcription regulation |
| NEDD4L | KCNAB2 | Unspecified | Ubiquitination |
| STAT3 | Survivin | activation | Transcription regulation |
| SUZ12 | GBL | Unspecified | co-regulation of transcription |
| AMFR | Ubiquitin | activation | Binding |
| BPAG1 | Tubulin (in microtubules) | activation | Binding |
| MDM2 | Ubiquitin | activation | Binding |
| VRK1 | BCRP1 | inhibition | Phosphorylation |
| N-CoR | LXR-alpha | inhibition | Binding |
| TIP30 | NDPK B | activation | Phosphorylation |
| GSK3 beta | Cyclin D3 | inhibition | Phosphorylation |
| HSF1 | HSC70 | Unspecified | Transcription regulation |
| FDPS | H-Ras | activation | Covalent modification |
| Ubiquitin | Survivin | inhibition | Binding |
| DBI | PBR | activation | Binding |
| NRF1 | ERp29 | Unspecified | Transcription regulation |
| CRP3 (MLP) | Cofilin, muscle | activation | Binding |
| FKBP12 | mTOR | inhibition | Binding |
| RPS19 | MIF | inhibition | Binding |
| ERK2 (MAPK1) | ERR1 | activation | Phosphorylation |
| Caspase-7 | Plectin 1 | Unspecified | Cleavage |
| STAT3 | IBP4 | Unspecified | Transcription regulation |
| MyD88 | mTOR | activation | Binding |
| DAPK2 | MLC2 | Unspecified | Phosphorylation |
| GSK3 beta | MARK2 | activation | Phosphorylation |
| Microcephalin | Caspase-7 | activation | co-regulation of transcription |
| Gapdh | G3P1 | Unspecified | Group Relation |
| KLF5 | Survivin | activation | Transcription regulation |
| STAT3 | CD151 | Unspecified | Transcription regulation |
| NRF1 | NDUFS6 | Unspecified | Transcription regulation |
| TTC3 | Ubiquitin | activation | Binding |
| MDM2 | MDM2 | activation | Transport |
| UBCH7 | Ubiquitin C | activation | Binding |
| MAPKAPK2 | CDC25C | inhibition | Phosphorylation |
| PKC-zeta | MARCKS | inhibition | Phosphorylation |
| STAT3 | Metallothionein-I | Unspecified | Transcription regulation |
| Caveolin-3 | G-protein alpha-o | inhibition | Binding |
| ZFP36(Tristetraprolin) | SMIF | activation | Binding |
| PARP-1 | Vimentin | activation | co-regulation of transcription |
| MM-1 | ACTB | activation | Binding |
| BRM | Myocardin | activation | Binding |
| IRF1 | PSMB10 | Unspecified | Transcription regulation |
| GGTase-II | PTP4A2 | activation | Covalent modification |
| Caspase-7 | VCP | inhibition | Cleavage |
| ERR1 | NDUFA10 | Unspecified | Transcription regulation |
| GSK3 beta | ZFP36(Tristetraprolin) | Unspecified | Phosphorylation |
| SUZ12 | CINP | Unspecified | co-regulation of transcription |
| PKC-zeta | Desmin | Unspecified | Phosphorylation |
| H-Ras | MDM2 | inhibition | Binding |
| PERC | NRF1 | activation | Binding |
| C/EBP zeta | ALDX | activation | Transcription regulation |
| NRF1 | ATPI | Unspecified | Transcription regulation |
| Ubiquitin | COPE | inhibition | Binding |
| N-CoR | PI3K reg class IA (p85-alpha) | inhibition | Binding |
| C/EBP zeta | AK1BA | Unspecified | Transcription regulation |
| POMGNT1 | Dystroglycan | Unspecified | Covalent modification |
| p120GAP | Aurora-A | inhibition | Binding |
| AMFR | IP3R1 | inhibition | Binding |
| CIN85 | PI3K reg class IA (p85-alpha) | inhibition | Binding |
| NRF1 | ATP5L | Unspecified | Transcription regulation |
| TOPORS | PARP-1 | Unspecified | Sumoylation |
| PKC-zeta | H-Ras | activation | Binding |
| Miz-1 | p21 | activation | Transcription regulation |
| Ubiquitin | Thymidylate kinase | inhibition | Binding |
| NRAMP1 | p21 | Unspecified | co-regulation of transcription |
| TOPORS | DJ-1 | Unspecified | Sumoylation |
| NRF1 | UQCR10 | Unspecified | Transcription regulation |
| TEF-4 | Dynein 1, cytoplasmic, heavy chain | Unspecified | Transcription regulation |
| LKB1 | STAT3 | inhibition | Binding |
| GAB1 | MAP3K3 | inhibition | Binding |
| IRF1 | NDUFC2 | Unspecified | Transcription regulation |
| PKC-zeta | GSTP1 | activation | Phosphorylation |
| MDM2 | PSMD4 | Unspecified | Ubiquitination |
| ZFP36(Tristetraprolin) | IEX1 | inhibition | Binding |
| NDPK A | NDPK complex | Unspecified | Complex Subunit |
| Alpha-parvin | ILK | activation | Binding |
| MARCKS | Calmodulin | inhibition | Binding |
| E2F6 | ART-27 | inhibition | Transcription regulation |
| GCP16 | N-Ras | activation | Covalent modification |
