# Supplementary material for: Cardiomyocyte Microvesicles Contain DNA/RNA and Convey Biological Messages to Target Cells
Source: PLoS One. 2012 Apr 10;7(4):e34653. doi: 10.1371/journal.pone.0034653 (PMC3323564; doi:10.1371/journal.pone.0034653)
Supplement: Table S3 — Differentially expressed genes in Fb after exosome incubation. Fibroblasts were transfected with microvesicles/exosomes and a clearcut effect on gene expression was found. After ultracentrifugation the microvesicle/exosome-enriched pellet resuspended in fresh DMEM that induced about 70% more changes (65 up and 96 down), here listed, than the microvesicle/exosome-deficient supernatant that induced only 96 changes (21 up, 75 down) when incubated with the fibroblast for 48 h. (DOC) [file pone.0034653.s006.doc]

Supplemental Table 3

| **Differentially expressed genes in Fb after exosome incubation** | | | | |
| --- | --- | --- | --- | --- |
| **SYMBOL** | **DEFINITION** | **Foldchange** | **FbPDiff Pval** | **ONTOLOGY_FUNCTION** |
| Spon2 | Mus musculus spondin 2, extracellular matrix protein (Spon2), mRNA. | 22,53 | 2,362841E-34 | Interacting selectively with any protein or protein complex (a complex of two or more proteins that may include other nonprotein molecules) [goid 5515] [evidence IEA] |
| Sepw1 | Mus musculus selenoprotein W, muscle 1 (Sepw1), mRNA. | 9,39 | 2,362841E-34 | Catalysis of an oxidation-reduction (redox) reaction, a reversible chemical reaction in which the oxidation state of an atom or atoms within a molecule is altered. One substrate acts as a hydrogen or electron donor and becomes oxidized, while the other acts as hydrogen or electron acceptor and becomes reduced [goid 16491] [evidence TAS]; Interacting selectively with selenium (Se) [goid 8430] [evidence IEA] |
| Calr | Mus musculus calreticulin (Calr), mRNA. | 8,94 | 0,000000001535944 | Interacting selectively with any metal ion [goid 46872] [evidence IEA]; Interacting selectively with any mono-, di- or trisaccharide carbohydrate [goid 5529] [evidence IEA]; Interacting selectively with zinc (Zn) ions [goid 8270] [evidence IEA]; Interacting selectively with an unfolded protein [goid 51082] [evidence IEA]; Interacting selectively with calcium ions (Ca2+) [goid 5509] [evidence IDA] |
| Cyp2d22 | Mus musculus cytochrome P450, family 2, subfamily d, polypeptide 22 (Cyp2d22), mRNA. | 7,14 | 0,000006787509 | Interacting selectively with iron (Fe) ions [goid 5506] [evidence IEA]; Interacting selectively with any metal ion [goid 46872] [evidence IEA]; Catalysis of the incorporation of one atom from molecular oxygen into a compound and the reduction of the other atom of oxygen to water [goid 4497] [evidence IEA]; Catalysis of an oxidation-reduction (redox) reaction, a reversible chemical reaction in which the oxidation state of an atom or atoms within a molecule is altered. One substrate acts as a hydrogen or electron donor and becomes oxidized, while the other acts as hydrogen or electron acceptor and becomes reduced [goid 16491] [evidence IEA] |
| Hic1 | Mus musculus hypermethylated in cancer 1 (Hic1), transcript variant 1, mRNA. | 6,90 | 2,362841E-34 | Interacting selectively with zinc (Zn) ions [goid 8270] [evidence IEA]; Interacting selectively with DNA (deoxyribonucleic acid) [goid 3677] [evidence IEA]; Interacting selectively with any metal ion [goid 46872] [evidence IEA]; Interacting selectively with any protein or protein complex (a complex of two or more proteins that may include other nonprotein molecules) [goid 5515] [evidence IEA]; Interacting selectively with any nucleic acid [goid 3676] [evidence IEA] |
| Cyp51 | Mus musculus cytochrome P450, family 51 (Cyp51), mRNA. | 6,78 | 0,000007790308 | Catalysis of the transfer of a methyl group to an acceptor molecule [goid 8168] [evidence IEA]; Interacting selectively with iron (Fe) ions [goid 5506] [evidence IEA]; Interacting selectively with any metal ion [goid 46872] [evidence IEA]; Catalysis of the reaction: obtusifoliol + 3 O2 + 3 NADPH + 3 H+ = 4-alpha-methyl-5-alpha-ergosta-8,14,24(28)-trien-3-beta-ol + formate + 3 NADP+ + 3 H2O [goid 8398] [evidence TAS] |
| Hist1h2ad | Mus musculus histone cluster 1, H2ad (Hist1h2ad), mRNA. | 6,14 | 0,000000006948909 | Interacting selectively with DNA (deoxyribonucleic acid) [goid 3677] [evidence IEA]; Elemental activities, such as catalysis or binding, describing the actions of a gene product at the molecular level. A given gene product may exhibit one or more molecular functions [goid 3674] [evidence ND ] |
| Sqle | Mus musculus squalene epoxidase (Sqle), mRNA. | 5,74 | 0,0000002510038 | Catalysis of an oxidation-reduction (redox) reaction, a reversible chemical reaction in which the oxidation state of an atom or atoms within a molecule is altered. One substrate acts as a hydrogen or electron donor and becomes oxidized, while the other acts as hydrogen or electron acceptor and becomes reduced [goid 16491] [evidence IEA]; Catalysis of the formation of a 2,3-epoxide in squalene [goid 4506] [evidence IEA] |
| Ctgf | Mus musculus connective tissue growth factor (Ctgf), mRNA. | 5,46 | 0,00000004410484 | Interacting selectively with any protein or protein complex (a complex of two or more proteins that may include other nonprotein molecules) [goid 5515] [evidence IEA]; Interacting selectively with an insulin-like growth factor, any member of a group of polypeptides that are structurally homologous to insulin and share many of its biological activities, but are immunologically distinct from it [goid 5520] [evidence IEA]; Interacting selectively with heparin, any member of a group of glycosaminoglycans found mainly as an intracellular component of mast cells and which consist predominantly of alternating alpha1-4-linked D-galactose and N-acetyl-D-glucosamine-6-sulfate residues [goid 8201] [evidence IDA]; Interacting selectively with an integrin [goid 5178] [evidence IDA] |
| Fdps | Mus musculus farnesyl diphosphate synthetase (Fdps), mRNA. | 5,29 | 3,169931E-13 | Catalysis of the transfer of a group, e.g. a methyl group, glycosyl group, acyl group, phosphorus-containing, or other groups, from one compound (generally regarded as the donor) to another compound (generally regarded as the acceptor). Transferase is the systematic name for any enzyme of EC class 2 [goid 16740] [evidence IEA]; Catalysis of the reaction: dimethylallyl diphosphate + isopentenyl diphosphate = diphosphate + geranyl diphosphate [goid 4161] [evidence IEA]; Catalysis of the reaction: geranyl diphosphate + isopentenyl diphosphate = diphosphate + trans,trans-farnesyl diphosphate [goid 4337] [evidence IEA] |
| Asna1 | Mus musculus arsA arsenite transporter, ATP-binding, homolog 1 (bacterial) (Asna1), mRNA. | 5,24 | 2,536671E-10 | Catalysis of the hydrolysis of various bonds, e.g. C-O, C-N, C-C, phosphoric anhydride bonds, etc. Hydrolase is the systematic name for any enzyme of EC class 3 [goid 16787] [evidence IEA]; Interacting selectively with ATP, adenosine 5'-triphosphate, a universally important coenzyme and enzyme regulator [goid 5524] [evidence IEA]; Interacting selectively with a nucleotide, any compound consisting of a nucleoside that is esterified with (ortho)phosphate or an oligophosphate at any hydroxyl group on the ribose or deoxyribose moiety [goid 166] [evidence IEA]; Catalysis of the transfer of a solute or solutes from one side of a membrane to the other according to the reaction: ATP + H2O + arsenite(in) = ADP + phosphate + arsenite(out) [goid 15446] [evidence IEA] |
| Hist2h2ac | Mus musculus histone cluster 2, H2ac (Hist2h2ac), mRNA. | 5,18 | 3,890371E-12 | Interacting selectively with DNA (deoxyribonucleic acid) [goid 3677] [evidence IEA] |
| Tmsb10 | Mus musculus thymosin, beta 10 (Tmsb10), mRNA. | 4,60 | 0,006831828 | Interacting selectively with monomeric or multimeric forms of actin, including actin filaments [goid 3779] [evidence IEA]; Interacting selectively with monomeric actin, also known as G-actin [goid 3785] [evidence TAS] |
| Hist1h2ao | Mus musculus histone cluster 1, H2ao (Hist1h2ao), mRNA. | 4,49 | 0,000000001164407 | Interacting selectively with DNA (deoxyribonucleic acid) [goid 3677] [evidence IEA] |
| LOC100046120 | PREDICTED: Mus musculus similar to clusterin (LOC100046120), mRNA. | 4,48 | 0,0009037415 |  |
| Hist1h2ah | Mus musculus histone cluster 1, H2ah (Hist1h2ah), mRNA. | 4,19 | 0,00000003075581 | Interacting selectively with DNA (deoxyribonucleic acid) [goid 3677] [evidence IEA] |
| Tpm1 | Mus musculus tropomyosin 1, alpha (Tpm1), mRNA. | 4,14 | 1,278905E-11 | Interacting selectively with monomeric or multimeric forms of actin, including actin filaments [goid 3779] [evidence IEA]; The action of a molecule that contributes to the structural integrity of a cytoskeletal structure [goid 5200] [evidence TAS] |
| Mvd | Mus musculus mevalonate (diphospho) decarboxylase (Mvd), mRNA. | 4,04 | 0,007484708 | Catalysis of the cleavage of C-C, C-O, C-N and other bonds by other means than by hydrolysis or oxidation, or conversely adding a group to a double bond. They differ from other enzymes in that two substrates are involved in one reaction direction, but only one in the other direction. When acting on the single substrate, a molecule is eliminated and this generates either a new double bond or a new ring [goid 16829] [evidence IEA]; Catalysis of the transfer of a phosphate group, usually from ATP, to a substrate molecule [goid 16301] [evidence IEA]; Interacting selectively with ATP, adenosine 5'-triphosphate, a universally important coenzyme and enzyme regulator [goid 5524] [evidence IEA]; Catalysis of the reaction: ATP + (R)-5-diphosphomevalonate = ADP + phosphate + isopentenyl diphosphate + CO2 [goid 4163] [evidence IEA] |
| Igfbp6 | Mus musculus insulin-like growth factor binding protein 6 (Igfbp6), mRNA. | 3,87 | 4,217387E-12 | Interacting selectively with any growth factor, proteins or polypeptides that stimulate a cell or organism to grow or proliferate [goid 19838] [evidence IEA]; Interacting selectively with an insulin-like growth factor, any member of a group of polypeptides that are structurally homologous to insulin and share many of its biological activities, but are immunologically distinct from it [goid 5520] [evidence IEA] |
| Rbp1 |  | 3,61 | 0,01423251 | Interacting selectively with retinoids, any member of a class of isoprenoids that contain or are derived from four prenyl groups linked head-to-tail. Retinoids include retinol and retinal and structurally similar natural derivatives or synthetic compounds, but need not have vitamin A activity [goid 5501] [evidence TAS]; Interacting selectively with retinol, vitamin A1, 2,6,6-trimethyl-1-(9'-hydroxy-3',7'-dimethylnona-1',3',5',7'-tetraenyl)cyclohex-1-ene, one of the three components that makes up vitamin A. Retinol is an intermediate in the vision cycle and it also plays a role in growth and differentiation [goid 19841] [evidence IEA]; Interacting selectively with retinal, one of the forms of vitamin A. Retinal plays an important role in the visual process in most vertebrates, combining with opsins to form visual pigments in the retina [goid 16918] [evidence IEA]; Enables the directed movement of substances (such as macromolecules, small molecules, ions) into, out of, within or between cells [goid 5215] [evidence IEA]; The selective, often stoichiometric, interaction of a molecule with one or more specific sites on another molecule [goid 5488] [evidence IEA]; Interacting selectively with a lipid [goid 8289] [evidence IEA] |
| Hist1h2ag | Mus musculus histone cluster 1, H2ag (Hist1h2ag), mRNA. | 3,55 | 0,000003059249 | Elemental activities, such as catalysis or binding, describing the actions of a gene product at the molecular level. A given gene product may exhibit one or more molecular functions [goid 3674] [evidence ND ]; Interacting selectively with DNA (deoxyribonucleic acid) [goid 3677] [evidence IEA] |
| LOC100040592 | PREDICTED: Mus musculus similar to Hmgcs1 protein, transcript variant 1 (LOC100040592), mRNA. | 3,37 | 0,000000001117378 |  |
| Hspa8 | Mus musculus heat shock protein 8 (Hspa8), mRNA. | 3,35 | 0,000001231468 | Interacting selectively with ATP, adenosine 5'-triphosphate, a universally important coenzyme and enzyme regulator [goid 5524] [evidence IEA]; Interacting selectively with a nucleotide, any compound consisting of a nucleoside that is esterified with (ortho)phosphate or an oligophosphate at any hydroxyl group on the ribose or deoxyribose moiety [goid 166] [evidence IEA]; Interacting selectively with any protein or protein complex (a complex of two or more proteins that may include other nonprotein molecules) [goid 5515] [evidence IPI]; Catalysis of the reaction: ATP + H2O = ADP + phosphate to directly drive some other reaction, for example ion transport across a membrane [goid 42623] [evidence IDA]; Interacting selectively with an unfolded protein [goid 51082] [evidence IPI] |
| Gpx1 | Mus musculus glutathione peroxidase 1 (Gpx1), mRNA. | 3,34 | 0,0003078776 | Interacting selectively with selenium (Se) [goid 8430] [evidence IEA]; Catalysis of the reaction: donor + H2O2 = oxidized donor + 2 H2O [goid 4601] [evidence IEA]; Catalysis of an oxidation-reduction (redox) reaction, a reversible chemical reaction in which the oxidation state of an atom or atoms within a molecule is altered. One substrate acts as a hydrogen or electron donor and becomes oxidized, while the other acts as hydrogen or electron acceptor and becomes reduced [goid 16491] [evidence IEA]; Catalysis of the reaction: 2 glutathione + H2O2 = oxidized glutathione + 2 H2O [goid 4602] [evidence IDA]; Catalysis of the reaction: 2 glutathione + H2O2 = oxidized glutathione + 2 H2O [goid 4602] [evidence IMP]; Catalysis of the reaction: 2 glutathione + H2O2 = oxidized glutathione + 2 H2O [goid 4602] [evidence IMP]; Catalysis of the reaction: 2 glutathione + H2O2 = oxidized glutathione + 2 H2O [goid 4602] [evidence IMP] |
| 2700094K13Rik | Mus musculus RIKEN cDNA 2700094K13 gene (2700094K13Rik), transcript variant 2, mRNA. | 3,32 | 0,000002967943 | Interacting selectively with selenium (Se) [goid 8430] [evidence IEA] |
| Mmp2 | Mus musculus matrix metallopeptidase 2 (Mmp2), mRNA. | 3,28 | 0,000001658961 | Interacting selectively with any metal ion [goid 46872] [evidence IEA]; Catalysis of the hydrolysis of various bonds, e.g. C-O, C-N, C-C, phosphoric anhydride bonds, etc. Hydrolase is the systematic name for any enzyme of EC class 3 [goid 16787] [evidence IEA]; Interacting selectively with calcium ions (Ca2+) [goid 5509] [evidence IEA]; Catalysis of the hydrolysis of a peptide bond. A peptide bond is a covalent bond formed when the carbon atom from the carboxyl group of one amino acid shares electrons with the nitrogen atom from the amino group of a second amino acid [goid 8233] [evidence IEA]; Catalysis of the hydrolysis of peptide bonds by a mechanism in which water acts as a nucleophile, one or two metal ions hold the water molecule in place, and charged amino acid side chains are ligands for the metal ions [goid 8237] [evidence IEA]; Interacting selectively with zinc (Zn) ions [goid 8270] [evidence IEA]; Catalysis of a biochemical reaction at physiological temperatures. In biologically catalyzed reactions, the reactants are known as substrates, and the catalysts are naturally occurring macromolecular substances known as enzymes. Enzymes possess specific binding sites for substrates, and are usually composed wholly or largely of protein, but RNA that has catalytic activity (ribozyme) is often also regarded as enzymatic [goid 3824] [evidence IEA]; Catalysis of the hydrolysis of internal, alpha-peptide bonds in a polypeptide chain by a mechanism in which water acts as a nucleophile, one or two metal ions hold the water molecule in place, and charged amino acid side chains are ligands for the metal ions [goid 4222] [evidence IDA] |
| Pcolce | Mus musculus procollagen C-endopeptidase enhancer protein (Pcolce), mRNA. | 3,20 | 0,0000003594087 | Interacting selectively with any protein or protein complex (a complex of two or more proteins that may include other nonprotein molecules) [goid 5515] [evidence IEA] |
| Pttg1ip | Mus musculus pituitary tumor-transforming 1 interacting protein (Pttg1ip), mRNA. | 3,12 | 0,000000003692465 |  |
| F2r | Mus musculus coagulation factor II (thrombin) receptor (F2r), mRNA. | 3,00 | 0,0001119703 | A receptor that binds an extracellular ligand and transmits the signal to a heterotrimeric G-protein complex. These receptors are characteristically seven-transmembrane receptors and are made up of hetero- or homodimers [goid 4930] [evidence IEA]; Combining with an extracellular or intracellular messenger to initiate a change in cell activity [goid 4872] [evidence IEA]; Mediates the transfer of a signal from the outside to the inside of a cell by means other than the introduction of the signal molecule itself into the cell [goid 4871] [evidence IEA]; Combining with thrombin to initiate a change in cell activity [goid 15057] [evidence IEA] |
| Lox | Mus musculus lysyl oxidase (Lox), mRNA. | 2,97 | 0,007005257 | Interacting selectively with any metal ion [goid 46872] [evidence IEA]; Interacting selectively with copper (Cu) ions [goid 5507] [evidence IEA]; Catalysis of an oxidation-reduction (redox) reaction, a reversible chemical reaction in which the oxidation state of an atom or atoms within a molecule is altered. One substrate acts as a hydrogen or electron donor and becomes oxidized, while the other acts as hydrogen or electron acceptor and becomes reduced [goid 16491] [evidence IEA]; Catalysis of the reaction: peptidyl-L-lysyl-peptide + H2O + O2 = peptidyl-allysyl-peptide + NH3 + H2O2 [goid 4720] [evidence IDA]; Interacting selectively with any protein or protein complex (a complex of two or more proteins that may include other nonprotein molecules) [goid 5515] [evidence IPI] |
| Prl2c3 | Mus musculus prolactin family 2, subfamily c, member 3 (Prl2c3), mRNA. | 2,74 | 0,004770062 | The action characteristic of a hormone, any substance formed in very small amounts in one specialized organ or group of cells and carried (sometimes in the bloodstream) to another organ or group of cells in the same organism, upon which it has a specific regulatory action. The term was originally applied to agents with a stimulatory physiological action in vertebrate animals (as opposed to a chalone, which has a depressant action). Usage is now extended to regulatory compounds in lower animals and plants, and to synthetic substances having comparable effects [goid 5179] [evidence IDA]; The function that stimulates a cell to grow or proliferate. Most growth factors have other actions besides the induction of cell growth or proliferation [goid 8083] [evidence IDA] |
| Cfl2 | Mus musculus cofilin 2, muscle (Cfl2), mRNA. | 2,66 | 0,002213368 | Interacting selectively with monomeric or multimeric forms of actin, including actin filaments [goid 3779] [evidence IEA] |
| Wdr1 | Mus musculus WD repeat domain 1 (Wdr1), mRNA. | 2,59 | 0,01962348 | Interacting selectively with monomeric or multimeric forms of actin, including actin filaments [goid 3779] [evidence ISA] |
| Gstm2 | Mus musculus glutathione S-transferase, mu 2 (Gstm2), mRNA. | 2,53 | 0,01142714 | Catalysis of the transfer of a group, e.g. a methyl group, glycosyl group, acyl group, phosphorus-containing, or other groups, from one compound (generally regarded as the donor) to another compound (generally regarded as the acceptor). Transferase is the systematic name for any enzyme of EC class 2 [goid 16740] [evidence IEA]; Catalysis of the reaction: R-X + glutathione = H-X + R-S-glutathione. R may be an aliphatic, aromatic or heterocyclic group; X may be a sulfate, nitrile or halide group [goid 4364] [evidence IEA]; Interacting selectively with any protein or protein complex (a complex of two or more proteins that may include other nonprotein molecules) [goid 5515] [evidence IPI] |
| Rbbp7 | Mus musculus retinoblastoma binding protein 7 (Rbbp7), mRNA. | 2,50 | 0,03631707 | Interacting selectively with any protein or protein complex (a complex of two or more proteins that may include other nonprotein molecules) [goid 5515] [evidence IPI]; Any transcription regulator activity that prevents or downregulates transcription [goid 16564] [evidence IDA] |
| S100a6 | Mus musculus S100 calcium binding protein A6 (calcyclin) (S100a6), mRNA. | 2,50 | 0,0002110647 | The function that stimulates a cell to grow or proliferate. Most growth factors have other actions besides the induction of cell growth or proliferation [goid 8083] [evidence IEA]; Interacting selectively with calcium ions (Ca2+) [goid 5509] [evidence IDA]; Interacting selectively with any protein or protein complex (a complex of two or more proteins that may include other nonprotein molecules) [goid 5515] [evidence IPI]; Interacting selectively with zinc (Zn) ions [goid 8270] [evidence IDA] |
| Apoa1bp | Mus musculus apolipoprotein A-I binding protein (Apoa1bp), mRNA. | 2,36 | 0,03253179 | Interacting selectively with any protein or protein complex (a complex of two or more proteins that may include other nonprotein molecules) [goid 5515] [evidence ISO] |
| Ipo5 | Mus musculus importin 5 (Ipo5), mRNA. | 2,33 | 0,0004816293 | Interacting selectively with any protein or protein complex (a complex of two or more proteins that may include other nonprotein molecules) [goid 5515] [evidence IPI]; Enables the directed movement of proteins into, out of, within or between cells [goid 8565] [evidence IEA]; The selective, often stoichiometric, interaction of a molecule with one or more specific sites on another molecule [goid 5488] [evidence IEA] |
| Gsta4 | Mus musculus glutathione S-transferase, alpha 4 (Gsta4), mRNA. | 2,22 | 0,0003850815 | Catalysis of the transfer of a group, e.g. a methyl group, glycosyl group, acyl group, phosphorus-containing, or other groups, from one compound (generally regarded as the donor) to another compound (generally regarded as the acceptor). Transferase is the systematic name for any enzyme of EC class 2 [goid 16740] [evidence IEA]; Catalysis of the reaction: R-X + glutathione = H-X + R-S-glutathione. R may be an aliphatic, aromatic or heterocyclic group; X may be a sulfate, nitrile or halide group [goid 4364] [evidence TAS]; Catalysis of the reaction: R-X + glutathione = H-X + R-S-glutathione. R may be an aliphatic, aromatic or heterocyclic group; X may be a sulfate, nitrile or halide group [goid 4364] [evidence ISO] |
| Fscn1 | Mus musculus fascin homolog 1, actin bundling protein (Strongylocentrotus purpuratus) (Fscn1), mRNA. | 2,18 | 0,00001686784 | Interacting selectively with monomeric or multimeric forms of actin, including actin filaments [goid 3779] [evidence IEA]; Interacting selectively with two or more protein molecules, or a protein and another macromolecule or complex, simultaneously, thereby physically linking the bound proteins or complexes to each other [goid 30674] [evidence IEA]; Interacting selectively with an actin filament, also known as F-actin, a helical filamentous polymer of globular G-actin subunits [goid 51015] [evidence IEA] |
| Maged2 | Mus musculus melanoma antigen, family D, 2 (Maged2), mRNA. | 2,14 | 0,0008066127 | Elemental activities, such as catalysis or binding, describing the actions of a gene product at the molecular level. A given gene product may exhibit one or more molecular functions [goid 3674] [evidence ND ] |
| LOC100041835 | PREDICTED: Mus musculus similar to H+ ATP synthase (LOC100041835), mRNA. | 2,09 | 0,00002304403 |  |
| Copz1 | Mus musculus coatomer protein complex, subunit zeta 1 (Copz1), mRNA. | 2,06 | 0,00006149477 | Interacting selectively with any protein or protein complex (a complex of two or more proteins that may include other nonprotein molecules) [goid 5515] [evidence IEA] |
| Ssr2 | Mus musculus signal sequence receptor, beta (Ssr2), mRNA. | 1,95 | 0,008792165 |  |
| Ndufa6 | Mus musculus NADH dehydrogenase (ubiquinone) 1 alpha subcomplex, 6 (B14) (Ndufa6), nuclear gene encoding mitochondrial protein, mRNA. | 1,95 | 0,0005866775 |  |
| Mrpl40 | Mus musculus mitochondrial ribosomal protein L40 (Mrpl40), nuclear gene encoding mitochondrial protein, mRNA. | 1,95 | 0,00736038 | Elemental activities, such as catalysis or binding, describing the actions of a gene product at the molecular level. A given gene product may exhibit one or more molecular functions [goid 3674] [evidence ND ] |
| Bloc1s1 | Mus musculus biogenesis of lysosome-related organelles complex-1, subunit 1 (Bloc1s1), mRNA. | 1,94 | 0,008735048 | Interacting selectively with any protein or protein complex (a complex of two or more proteins that may include other nonprotein molecules) [goid 5515] [evidence IPI] |
| LOC100046650 | PREDICTED: Mus musculus similar to PRELI domain containing 1 (LOC100046650), mRNA. | 1,92 | 0,0008070499 |  |
| Pef1 | Mus musculus penta-EF hand domain containing 1 (Pef1), mRNA. | 1,92 | 0,0007115101 | Interacting selectively with calcium ions (Ca2+) [goid 5509] [evidence IEA] |
| Camk1 | Mus musculus calcium/calmodulin-dependent protein kinase I (Camk1), mRNA. | 1,91 | 0,009219905 | Catalysis of a biochemical reaction at physiological temperatures. In biologically catalyzed reactions, the reactants are known as substrates, and the catalysts are naturally occurring macromolecular substances known as enzymes. Enzymes possess specific binding sites for substrates, and are usually composed wholly or largely of protein, but RNA that has catalytic activity (ribozyme) is often also regarded as enzymatic [goid 3824] [evidence IEA]; Catalysis of the transfer of a phosphate group, usually from ATP, to a substrate molecule [goid 16301] [evidence IEA]; Interacting selectively with a nucleotide, any compound consisting of a nucleoside that is esterified with (ortho)phosphate or an oligophosphate at any hydroxyl group on the ribose or deoxyribose moiety [goid 166] [evidence IEA]; Catalysis of the transfer of a group, e.g. a methyl group, glycosyl group, acyl group, phosphorus-containing, or other groups, from one compound (generally regarded as the donor) to another compound (generally regarded as the acceptor). Transferase is the systematic name for any enzyme of EC class 2 [goid 16740] [evidence IEA]; Catalysis of the phosphorylation of an amino acid residue in a protein, usually according to the reaction: a protein + ATP = a phosphoprotein + ADP [goid 4672] [evidence IEA]; Interacting selectively with any protein or protein complex (a complex of two or more proteins that may include other nonprotein molecules) [goid 5515] [evidence IPI]; Catalysis of the reaction: ATP + a protein serine/threonine = ADP + protein serine/threonine phosphate [goid 4674] [evidence IEA]; Interacting selectively with calmodulin, a calcium-binding protein with many roles, both in the calcium-bound and calcium-free states [goid 5516] [evidence IEA]; Interacting selectively with ATP, adenosine 5'-triphosphate, a universally important coenzyme and enzyme regulator [goid 5524] [evidence IEA]; Catalysis of the reaction: ATP + a protein serine/threonine = ADP + protein serine/threonine phosphate, dependent on the presence of calcium-bound calmodulin [goid 4683] [evidence IEA] |
| Stx7 | Mus musculus syntaxin 7 (Stx7), mRNA. | 1,90 | 0,001569128 | Interacting selectively with iron (Fe) ions [goid 5506] [evidence IEA]; Acting as a marker to identify a membrane and interacting selectively with one or more SNAREs on another membrane to mediate membrane fusion [goid 5484] [evidence IEA]; Interacting selectively with any protein or protein complex (a complex of two or more proteins that may include other nonprotein molecules) [goid 5515] [evidence IPI] |
| Cd47 | Mus musculus CD47 antigen (Rh-related antigen, integrin-associated signal transducer) (Cd47), mRNA. | 1,88 | 0,0006704471 | Interacting selectively with any protein or protein complex (a complex of two or more proteins that may include other nonprotein molecules) [goid 5515] [evidence IPI] |
| Aurka | Mus musculus aurora kinase A (Aurka), mRNA. | 1,85 | 0,01645386 | Catalysis of the transfer of a phosphate group, usually from ATP, to a substrate molecule [goid 16301] [evidence IEA]; Interacting selectively with a nucleotide, any compound consisting of a nucleoside that is esterified with (ortho)phosphate or an oligophosphate at any hydroxyl group on the ribose or deoxyribose moiety [goid 166] [evidence IEA]; Catalysis of the reaction: ATP + a protein serine/threonine = ADP + protein serine/threonine phosphate [goid 4674] [evidence IEA]; Catalysis of the transfer of a group, e.g. a methyl group, glycosyl group, acyl group, phosphorus-containing, or other groups, from one compound (generally regarded as the donor) to another compound (generally regarded as the acceptor). Transferase is the systematic name for any enzyme of EC class 2 [goid 16740] [evidence IEA]; Interacting selectively with ATP, adenosine 5'-triphosphate, a universally important coenzyme and enzyme regulator [goid 5524] [evidence IEA]; Catalysis of the phosphorylation of an amino acid residue in a protein, usually according to the reaction: a protein + ATP = a phosphoprotein + ADP [goid 4672] [evidence IEA]; Interacting selectively with a ubiquitin protein ligase enzyme, any of the E3 proteins [goid 31625] [evidence IPI] |
| AI662250 | Mus musculus expressed sequence AI662250 (AI662250), mRNA. | 1,84 | 0,04234628 | Interacting selectively with an intermediate filament, a distinct elongated structure, characteristically 10 nm in diameter, that occurs in the cytoplasm of higher eukaryotic cells. Intermediate filaments form a fibrous system, composed of chemically heterogeneous subunits and involved in mechanically integrating the various components of the cytoplasmic space [goid 19215] [evidence ISO] |
| Ugt1a10 | Mus musculus UDP glycosyltransferase 1 family, polypeptide A10 (Ugt1a10), mRNA. | 1,83 | 0,008735048 | Catalysis of the transfer of a group, e.g. a methyl group, glycosyl group, acyl group, phosphorus-containing, or other groups, from one compound (generally regarded as the donor) to another compound (generally regarded as the acceptor). Transferase is the systematic name for any enzyme of EC class 2 [goid 16740] [evidence IEA]; Catalysis of the transfer of a glycosyl group from one compound (donor) to another (acceptor) [goid 16757] [evidence IEA] |
| Naca | Mus musculus nascent polypeptide-associated complex alpha polypeptide (Naca), mRNA. | 1,80 | 0,02601359 | Interacting selectively with DNA (deoxyribonucleic acid) [goid 3677] [evidence IEA]; Interacting selectively with TATA-binding protein (TBP), a component of various transcription factors [goid 17025] [evidence IDA]; The function of a transcription cofactor that activates transcription from a RNA polymerase II promoter; does not bind DNA itself [goid 3713] [evidence IDA] |
| Ly6e | Mus musculus lymphocyte antigen 6 complex, locus E (Ly6e), mRNA. | 1,79 | 0,007190114 |  |
| Ttyh3 | Mus musculus tweety homolog 3 (Drosophila) (Ttyh3), mRNA. | 1,79 | 0,04040768 | Interacting selectively with chloride ions (Cl-) [goid 31404] [evidence IEA]; Interacting selectively with calcium ions (Ca2+) [goid 5509] [evidence IEA]; Catalysis of facilitated diffusion of a chloride (by an energy-independent process) involving passage through a transmembrane aqueous pore or channel without evidence for a carrier-mediated mechanism [goid 5254] [evidence ISO]; Catalysis of facilitated diffusion of an ion (by an energy-independent process) by passage through a transmembrane aqueous pore or channel without evidence for a carrier-mediated mechanism [goid 5216] [evidence IEA] |
| Cmtm7 | Mus musculus CKLF-like MARVEL transmembrane domain containing 7 (Cmtm7), mRNA. | 1,78 | 0,0486142 | Functions to control the survival, growth, differentiation and effector function of tissues and cells [goid 5125] [evidence IEA] |
| Tfg | Mus musculus Trk-fused gene (Tfg), mRNA. | 1,75 | 0,002823327 | Interacting selectively with any protein or protein complex (a complex of two or more proteins that may include other nonprotein molecules) [goid 5515] [evidence IPI]; Interacting selectively with an identical protein or proteins [goid 42802] [evidence IPI] |
| Nmt1 | Mus musculus N-myristoyltransferase 1 (Nmt1), mRNA. | 1,71 | 0,03552753 | Catalysis of the transfer of a group, e.g. a methyl group, glycosyl group, acyl group, phosphorus-containing, or other groups, from one compound (generally regarded as the donor) to another compound (generally regarded as the acceptor). Transferase is the systematic name for any enzyme of EC class 2 [goid 16740] [evidence IEA]; Catalysis of the generalized reaction: acyl-carrier + reactant = acyl-reactant + carrier [goid 8415] [evidence IEA]; Catalysis of the reaction: tetradecanoyl-CoA + glycyl-peptide = CoA + N-tetradecanoylglycyl-peptide [goid 4379] [evidence TAS]; Catalysis of the reaction: tetradecanoyl-CoA + glycyl-peptide = CoA + N-tetradecanoylglycyl-peptide [goid 4379] [evidence ISA]; Catalysis of the reaction: tetradecanoyl-CoA + glycyl-peptide = CoA + N-tetradecanoylglycyl-peptide [goid 4379] [evidence IMP] |
| Arl2 | Mus musculus ADP-ribosylation factor-like 2 (Arl2), mRNA. | 1,66 | 0,01681183 | Interacting selectively with GTP, guanosine triphosphate [goid 5525] [evidence IEA]; Interacting selectively with a nucleotide, any compound consisting of a nucleoside that is esterified with (ortho)phosphate or an oligophosphate at any hydroxyl group on the ribose or deoxyribose moiety [goid 166] [evidence IEA]; Interacting selectively with any protein or protein complex (a complex of two or more proteins that may include other nonprotein molecules) [goid 5515] [evidence IPI] |
| Pdlim7 |  | 1,61 | 0,03243802 | Interacting selectively with any protein or protein complex (a complex of two or more proteins that may include other nonprotein molecules) [goid 5515] [evidence IPI]; Interacting selectively with zinc (Zn) ions [goid 8270] [evidence IEA]; Interacting selectively with any metal ion [goid 46872] [evidence IEA] |
| Jtb | Mus musculus jumping translocation breakpoint (Jtb), mRNA. | 1,57 | 0,01806324 |  |
| Iscu | Mus musculus IscU iron-sulfur cluster scaffold homolog (E. coli) (Iscu), mRNA. | 1,55 | 0,03467682 | Interacting selectively with iron (Fe) ions [goid 5506] [evidence IEA]; Interacting selectively with any metal ion [goid 46872] [evidence IEA]; Interacting selectively with an iron-sulfur cluster, a combination of iron and sulfur atoms [goid 51536] [evidence IEA]; Interacting selectively with any protein or protein complex (a complex of two or more proteins that may include other nonprotein molecules) [goid 5515] [evidence IEA] |
| **Differentially expressed genes in Fb after exosome incubation** | | | | |
| **SYMBOL** | **DEFINITION** | **Foldchange** | **FbPDiff Pval** | **ONTOLOGY_FUNCTION** |
| Nlgn2 | Mus musculus neuroligin 2 (Nlgn2), mRNA. | 0,08 | 2,893683E-13 | Interacting selectively with any protein or protein complex (a complex of two or more proteins that may include other nonprotein molecules) [goid 5515] [evidence IEA] |
| Pdzk1ip1 | Mus musculus PDZK1 interacting protein 1 (Pdzk1ip1), mRNA. | 0,14 | 0,000292739 | Elemental activities, such as catalysis or binding, describing the actions of a gene product at the molecular level. A given gene product may exhibit one or more molecular functions [goid 3674] [evidence ND ] |
| Hdac11 | Mus musculus histone deacetylase 11 (Hdac11), mRNA. | 0,15 | 0,000001570896 | Catalysis of the hydrolysis of acetyl groups from histones, proteins complexed to DNA in chromatin and chromosomes [goid 4407] [evidence TAS]; Catalysis of the hydrolysis of various bonds, e.g. C-O, C-N, C-C, phosphoric anhydride bonds, etc. Hydrolase is the systematic name for any enzyme of EC class 3 [goid 16787] [evidence IEA]; Interacting selectively with a transcription factor, any protein required to initiate or regulate transcription [goid 8134] [evidence TAS]; Interacting selectively with any protein or protein complex (a complex of two or more proteins that may include other nonprotein molecules) [goid 5515] [evidence IPI] |
| Osr1 | Mus musculus odd-skipped related 1 (Drosophila) (Osr1), mRNA. | 0,16 | 0,0000003302154 | Interacting selectively with any metal ion [goid 46872] [evidence IEA]; Interacting selectively with zinc (Zn) ions [goid 8270] [evidence IEA]; Interacting selectively with any nucleic acid [goid 3676] [evidence IEA] |
| Chrd | Mus musculus chordin (Chrd), mRNA. | 0,18 | 0,01036978 | Interacting selectively with any protein or protein complex (a complex of two or more proteins that may include other nonprotein molecules) [goid 5515] [evidence IPI]; Interacting selectively with heparin, any member of a group of glycosaminoglycans found mainly as an intracellular component of mast cells and which consist predominantly of alternating alpha1-4-linked D-galactose and N-acetyl-D-glucosamine-6-sulfate residues [goid 8201] [evidence IDA]; Interacting selectively with syndecan, an integral membrane proteoglycan (250-300 kDa) associated largely with epithelial cells [goid 45545] [evidence IDA] |
| Pcif1 | Mus musculus PDX1 C-terminal inhibiting factor 1 (Pcif1), mRNA. | 0,19 | 0,00000669208 | Interacting selectively with any protein or protein complex (a complex of two or more proteins that may include other nonprotein molecules) [goid 5515] [evidence IEA] |
| Ass1 | Mus musculus argininosuccinate synthetase 1 (Ass1), mRNA. | 0,19 | 0,000008049805 | Interacting selectively with a nucleotide, any compound consisting of a nucleoside that is esterified with (ortho)phosphate or an oligophosphate at any hydroxyl group on the ribose or deoxyribose moiety [goid 166] [evidence IEA]; Catalysis of the ligation of two substances with concomitant breaking of a diphosphate linkage, usually in a nucleoside triphosphate. Ligase is the systematic name for any enzyme of EC class 6 [goid 16874] [evidence IEA]; Interacting selectively with ATP, adenosine 5'-triphosphate, a universally important coenzyme and enzyme regulator [goid 5524] [evidence IEA]; Catalysis of the reaction: ATP + L-citrulline + L-aspartate = AMP + diphosphate + (N(omega)-L-arginino)succinate [goid 4055] [evidence IEA] |
| Vamp2 | Mus musculus vesicle-associated membrane protein 2 (Vamp2), mRNA. | 0,21 | 0,01564976 | Interacting selectively with phospholipids, a class of lipids containing phosphoric acid as a mono- or diester [goid 5543] [evidence IDA]; Interacting selectively with calmodulin, a calcium-binding protein with many roles, both in the calcium-bound and calcium-free states [goid 5516] [evidence IDA]; Interacting selectively with a SNARE (soluble N-ethylmaleimide-sensitive factor attached protein receptor) protein [goid 149] [evidence IDA]; Interacting selectively with a SNARE (soluble N-ethylmaleimide-sensitive factor attached protein receptor) protein [goid 149] [evidence ISO]; Interacting selectively with the SNAP receptor syntaxin-1 [goid 17075] [evidence ISO] |
| Nrbp2 | Mus musculus nuclear receptor binding protein 2 (Nrbp2), mRNA. | 0,22 | 0,000003255524 | Interacting selectively with ATP, adenosine 5'-triphosphate, a universally important coenzyme and enzyme regulator [goid 5524] [evidence IEA]; Catalysis of the phosphorylation of an amino acid residue in a protein, usually according to the reaction: a protein + ATP = a phosphoprotein + ADP [goid 4672] [evidence IEA] |
| C4b | Mus musculus complement component 4B (Childo blood group) (C4b), mRNA. XM_921663 XM_921673 XM_921676 XM_921678 | 0,23 | 3,150456E-34 | Stops, prevents or reduces the activity of an endopeptidase, any enzyme that hydrolyzes nonterminal peptide bonds in polypeptides [goid 4866] [evidence IEA]; Interacting selectively with any protein or protein complex (a complex of two or more proteins that may include other nonprotein molecules) [goid 5515] [evidence IEA] |
| Glis1 | Mus musculus GLIS family zinc finger 1 (Glis1), mRNA. | 0,24 | 1,236268E-16 | Interacting selectively with zinc (Zn) ions [goid 8270] [evidence IEA]; Interacting selectively with any metal ion [goid 46872] [evidence IEA]; Interacting selectively with any nucleic acid [goid 3676] [evidence IEA]; Interacting selectively with DNA (deoxyribonucleic acid) [goid 3677] [evidence IDA]; Functions to enable the transcription of specific, or specific sets, of genes by RNA polymerase II [goid 3704] [evidence IDA] |
| Kcnab2 | Mus musculus potassium voltage-gated channel, shaker-related subfamily, beta member 2 (Kcnab2), mRNA. | 0,24 | 0,0001569241 | Interacting selectively with potassium (K+) ions [goid 30955] [evidence IEA]; Catalysis of the transmembrane transfer of an ion by a voltage-gated channel. An ion is an atom or group of atoms carrying an electric charge by virtue of having gained or lost one or more electrons [goid 5244] [evidence IEA]; Catalysis of facilitated diffusion of an ion (by an energy-independent process) by passage through a transmembrane aqueous pore or channel without evidence for a carrier-mediated mechanism [goid 5216] [evidence IEA]; Catalysis of the transmembrane transfer of a potassium ion by a voltage-gated channel [goid 5249] [evidence IEA]; Catalysis of an oxidation-reduction (redox) reaction, a reversible chemical reaction in which the oxidation state of an atom or atoms within a molecule is altered. One substrate acts as a hydrogen or electron donor and becomes oxidized, while the other acts as hydrogen or electron acceptor and becomes reduced [goid 16491] [evidence IEA] |
| Crabp1 | Mus musculus cellular retinoic acid binding protein I (Crabp1), mRNA. | 0,25 | 0,0002915028 | Interacting selectively with retinol, vitamin A1, 2,6,6-trimethyl-1-(9'-hydroxy-3',7'-dimethylnona-1',3',5',7'-tetraenyl)cyclohex-1-ene, one of the three components that makes up vitamin A. Retinol is an intermediate in the vision cycle and it also plays a role in growth and differentiation [goid 19841] [evidence IEA]; Interacting selectively with retinoids, any member of a class of isoprenoids that contain or are derived from four prenyl groups linked head-to-tail. Retinoids include retinol and retinal and structurally similar natural derivatives or synthetic compounds, but need not have vitamin A activity [goid 5501] [evidence TAS]; Interacting selectively with retinal, one of the forms of vitamin A. Retinal plays an important role in the visual process in most vertebrates, combining with opsins to form visual pigments in the retina [goid 16918] [evidence IEA]; Enables the directed movement of substances (such as macromolecules, small molecules, ions) into, out of, within or between cells [goid 5215] [evidence IEA]; The selective, often stoichiometric, interaction of a molecule with one or more specific sites on another molecule [goid 5488] [evidence IEA]; Interacting selectively with a lipid [goid 8289] [evidence IEA] |
| Arhgap1 | Mus musculus Rho GTPase activating protein 1 (Arhgap1), mRNA. | 0,25 | 0,0002319251 | Increases the activity of a GTPase, an enzyme that catalyzes the hydrolysis of GTP [goid 5096] [evidence IEA]; Interacting selectively with a SH3 domain (Src homology 3) of a protein, small protein modules containing approximately 50 amino acid residues found in a great variety of intracellular or membrane-associated proteins [goid 17124] [evidence IEA]; Increases the rate of GTP hydrolysis by a GTPase of the Rho family [goid 5100] [evidence IDA]; Increases the rate of GTP hydrolysis by a GTPase of the Rho family [goid 5100] [evidence ISO]; Increases the rate of GTP hydrolysis by a GTPase of the Rac family [goid 30675] [evidence IDA] |
| Flii | Mus musculus flightless I homolog (Drosophila) (Flii), mRNA. | 0,25 | 0,002032654 | Interacting selectively with monomeric or multimeric forms of actin, including actin filaments [goid 3779] [evidence IEA]; Interacting selectively with any protein or protein complex (a complex of two or more proteins that may include other nonprotein molecules) [goid 5515] [evidence IEA] |
| Cercam | Mus musculus cerebral endothelial cell adhesion molecule (Cercam), mRNA. | 0,25 | 0,02066335 | Catalysis of the transfer of a group, e.g. a methyl group, glycosyl group, acyl group, phosphorus-containing, or other groups, from one compound (generally regarded as the donor) to another compound (generally regarded as the acceptor). Transferase is the systematic name for any enzyme of EC class 2 [goid 16740] [evidence IEA]; Catalysis of the transfer of a glycosyl group from one compound (donor) to another (acceptor) [goid 16757] [evidence IEA]; Interacting selectively with any protein or protein complex (a complex of two or more proteins that may include other nonprotein molecules) [goid 5515] [evidence IEA]; Elemental activities, such as catalysis or binding, describing the actions of a gene product at the molecular level. A given gene product may exhibit one or more molecular functions [goid 3674] [evidence ND ] |
| Sbf1 | Mus musculus SET binding factor 1 (Sbf1), mRNA. | 0,26 | 0,00004618154 | Catalysis of the hydrolysis of phosphoric monoesters, releasing inorganic phosphate [goid 16791] [evidence IEA]; Maintains the phosphorylation state of certain molecules by associating with them and preventing them from associating with active phosphatases, and thus inhibiting the enzyme activity without interacting with the enzyme. Often pertains to proteins belonging to dual-specificity phosphatase family but lacking critical active site residues [goid 1691] [evidence TAS] |
| Evi5l | Mus musculus ecotropic viral integration site 5 like (Evi5l), mRNA. | 0,27 | 5,344403E-21 | Increases the rate of GTP hydrolysis by a GTPase of the Rab family [goid 5097] [evidence ISO] |
| Ccnd1 | Mus musculus cyclin D1 (Ccnd1), mRNA. | 0,28 | 0,0006181179 | Interacting selectively with any protein or protein complex (a complex of two or more proteins that may include other nonprotein molecules) [goid 5515] [evidence IPI]; Catalysis of the transfer of a phosphate group, usually from ATP, to a substrate molecule [goid 16301] [evidence IDA]; Catalysis of the phosphorylation of an amino acid residue in a protein, usually according to the reaction: a protein + ATP = a phosphoprotein + ADP [goid 4672] [evidence IDA]; Modulates the activity of a cyclin-dependent protein kinase, enzymes of the protein kinase family that are regulated through association with cyclins and other proteins [goid 16538] [evidence IDA] |
| Uaca | Mus musculus uveal autoantigen with coiled-coil domains and ankyrin repeats (Uaca), mRNA. | 0,29 | 0,00000001053338 | Interacting selectively with any protein or protein complex (a complex of two or more proteins that may include other nonprotein molecules) [goid 5515] [evidence IPI] |
| Gng8 | Mus musculus guanine nucleotide binding protein (G protein), gamma 8 (Gng8), mRNA. | 0,29 | 2,248117E-20 | Mediates the transfer of a signal from the outside to the inside of a cell by means other than the introduction of the signal molecule itself into the cell [goid 4871] [evidence IEA] |
| Fgfrl1 | Mus musculus fibroblast growth factor receptor-like 1 (Fgfrl1), mRNA. | 0,30 | 6,973772E-21 | A receptor belonging to the FGFR family but lacking the tyrosine kinase domain [goid 1571] [evidence ISA]; Combining with an extracellular or intracellular messenger to initiate a change in cell activity [goid 4872] [evidence IEA]; Interacting selectively with any protein or protein complex (a complex of two or more proteins that may include other nonprotein molecules) [goid 5515] [evidence IPI] |
| Pak4 | Mus musculus p21 (CDKN1A)-activated kinase 4 (Pak4), mRNA. | 0,32 | 0,000000861473 | Catalysis of the reaction: ATP + a protein serine/threonine = ADP + protein serine/threonine phosphate [goid 4674] [evidence IEA]; Catalysis of the transfer of a group, e.g. a methyl group, glycosyl group, acyl group, phosphorus-containing, or other groups, from one compound (generally regarded as the donor) to another compound (generally regarded as the acceptor). Transferase is the systematic name for any enzyme of EC class 2 [goid 16740] [evidence IEA]; Catalysis of the transfer of a phosphate group, usually from ATP, to a substrate molecule [goid 16301] [evidence IEA]; Interacting selectively with ATP, adenosine 5'-triphosphate, a universally important coenzyme and enzyme regulator [goid 5524] [evidence IEA]; Interacting selectively with a nucleotide, any compound consisting of a nucleoside that is esterified with (ortho)phosphate or an oligophosphate at any hydroxyl group on the ribose or deoxyribose moiety [goid 166] [evidence IEA]; Catalysis of the phosphorylation of an amino acid residue in a protein, usually according to the reaction: a protein + ATP = a phosphoprotein + ADP [goid 4672] [evidence IEA] |
| Igfbp4 |  | 0,32 | 1,774952E-17 | Interacting selectively with any growth factor, proteins or polypeptides that stimulate a cell or organism to grow or proliferate [goid 19838] [evidence IEA]; Interacting selectively with an insulin-like growth factor, any member of a group of polypeptides that are structurally homologous to insulin and share many of its biological activities, but are immunologically distinct from it [goid 5520] [evidence IPI] |
| Txnip | Mus musculus thioredoxin interacting protein (Txnip), transcript variant 1, mRNA. | 0,32 | 3,921178E-13 | Stops, prevents or reduces the activity of an enzyme [goid 4857] [evidence IDA]; Interacting selectively with any protein or protein complex (a complex of two or more proteins that may include other nonprotein molecules) [goid 5515] [evidence IPI] |
| Rpl3 | Mus musculus ribosomal protein L3 (Rpl3), mRNA. | 0,32 | 0,0008353189 | The action of a molecule that contributes to the structural integrity of the ribosome [goid 3735] [evidence IEA]; Elemental activities, such as catalysis or binding, describing the actions of a gene product at the molecular level. A given gene product may exhibit one or more molecular functions [goid 3674] [evidence ND ] |
| Sdc3 | Mus musculus syndecan 3 (Sdc3), mRNA. | 0,32 | 1,074389E-26 | Interacting selectively with any protein component of any cytoskeleton (actin, microtubule, or intermediate filament cytoskeleton) [goid 8092] [evidence IEA] |
| Slc25a12 | Mus musculus solute carrier family 25 (mitochondrial carrier, Aralar), member 12 (Slc25a12), mRNA. | 0,32 | 0,006514703 | Interacting selectively with calcium ions (Ca2+) [goid 5509] [evidence IEA]; Enables the directed movement of substances (such as macromolecules, small molecules, ions) into, out of, within or between cells [goid 5215] [evidence IEA]; The selective, often stoichiometric, interaction of a molecule with one or more specific sites on another molecule [goid 5488] [evidence IEA] |
| Ap3m2 | Mus musculus adaptor-related protein complex 3, mu 2 subunit (Ap3m2), mRNA. | 0,33 | 0,00000007588607 | Interacting selectively with any protein or protein complex (a complex of two or more proteins that may include other nonprotein molecules) [goid 5515] [evidence IEA] |
| Csk | Mus musculus c-src tyrosine kinase (Csk), mRNA. | 0,33 | 0,000000006944882 | Catalysis of the transfer of a group, e.g. a methyl group, glycosyl group, acyl group, phosphorus-containing, or other groups, from one compound (generally regarded as the donor) to another compound (generally regarded as the acceptor). Transferase is the systematic name for any enzyme of EC class 2 [goid 16740] [evidence TAS]; Interacting selectively with ATP, adenosine 5'-triphosphate, a universally important coenzyme and enzyme regulator [goid 5524] [evidence TAS]; Catalysis of the transfer of a phosphate group, usually from ATP, to a substrate molecule [goid 16301] [evidence IEA]; Interacting selectively with a nucleotide, any compound consisting of a nucleoside that is esterified with (ortho)phosphate or an oligophosphate at any hydroxyl group on the ribose or deoxyribose moiety [goid 166] [evidence IEA]; Catalysis of the reaction: ATP + a protein tyrosine = ADP + protein tyrosine phosphate [goid 4713] [evidence IEA]; Catalysis of the phosphorylation of an amino acid residue in a protein, usually according to the reaction: a protein + ATP = a phosphoprotein + ADP [goid 4672] [evidence IEA]; Catalysis of the reaction: ATP + a protein-L-tyrosine = ADP + a protein-L-tyrosine phosphate [goid 4715] [evidence IEA]; Interacting selectively with any protein or protein complex (a complex of two or more proteins that may include other nonprotein molecules) [goid 5515] [evidence IPI] |
| Aplp2 | Mus musculus amyloid beta (A4) precursor-like protein 2 (Aplp2), transcript variant 3, mRNA. | 0,34 | 0,0005866775 | Interacting selectively with DNA (deoxyribonucleic acid) [goid 3677] [evidence IEA]; The selective, often stoichiometric, interaction of a molecule with one or more specific sites on another molecule [goid 5488] [evidence IEA]; Interacting selectively with any protein or protein complex (a complex of two or more proteins that may include other nonprotein molecules) [goid 5515] [evidence IPI] |
| Jun | Mus musculus Jun oncogene (Jun), mRNA. | 0,35 | 0,03483095 | Interacting selectively with any protein or protein complex (a complex of two or more proteins that may include other nonprotein molecules) [goid 5515] [evidence IPI]; Interacting selectively with DNA of a specific nucleotide composition, e.g. GC-rich DNA binding, or with a specific sequence motif or type of DNA e.g. promotor binding or rDNA binding [goid 43565] [evidence IEA]; Interacting selectively with DNA (deoxyribonucleic acid) [goid 3677] [evidence IDA]; The function of binding to a specific DNA sequence in order to modulate transcription. The transcription factor may or may not also interact selectively with a protein or macromolecular complex [goid 3700] [evidence IEA]; The formation of a protein dimer, a macromolecular structure consists of two noncovalently associated identical or nonidentical subunits [goid 46983] [evidence IEA]; Interacting selectively with DNA (deoxyribonucleic acid) [goid 3677] [evidence IDA] |
| Cnksr3 | Mus musculus Cnksr family member 3 (Cnksr3), mRNA. | 0,36 | 0,0359533 | Interacting selectively with any protein or protein complex (a complex of two or more proteins that may include other nonprotein molecules) [goid 5515] [evidence IEA] |
| Ank | Mus musculus progressive ankylosis (Ank), mRNA. | 0,36 | 0,002325801 | Catalysis of the transfer of phosphate (PO4 3-) ions from one side of a membrane to the other [goid 15114] [evidence IEA]; Catalysis of the transfer of inorganic diphosphate across a membrane [goid 30504] [evidence IDA] |
| Itpk1 | Mus musculus inositol 1,3,4-triphosphate 5/6 kinase (Itpk1), mRNA. | 0,37 | 0,0002653154 | Catalysis of the hydrolysis of various bonds, e.g. C-O, C-N, C-C, phosphoric anhydride bonds, etc. Hydrolase is the systematic name for any enzyme of EC class 3 [goid 16787] [evidence IEA]; Catalysis of the transfer of a phosphate group, usually from ATP, to a substrate molecule [goid 16301] [evidence IEA]; Catalysis of the geometric or structural changes within one molecule. Isomerase is the systematic name for any enzyme of EC class 5 [goid 16853] [evidence IEA]; Interacting selectively with magnesium (Mg) ions [goid 287] [evidence IEA]; Interacting selectively with a nucleotide, any compound consisting of a nucleoside that is esterified with (ortho)phosphate or an oligophosphate at any hydroxyl group on the ribose or deoxyribose moiety [goid 166] [evidence IEA]; Catalysis of the transfer of a group, e.g. a methyl group, glycosyl group, acyl group, phosphorus-containing, or other groups, from one compound (generally regarded as the donor) to another compound (generally regarded as the acceptor). Transferase is the systematic name for any enzyme of EC class 2 [goid 16740] [evidence IEA]; Interacting selectively with any metal ion [goid 46872] [evidence IEA]; Interacting selectively with ATP, adenosine 5'-triphosphate, a universally important coenzyme and enzyme regulator [goid 5524] [evidence IEA]; Catalysis of the reaction: 1D-myo-inositol 3,4,5,6-tetrakisphosphate + ATP = 1D-myo-inositol 1,3,4,5,6-pentakisphosphate + ADP [goid 47325] [evidence IEA]; Catalysis of a biochemical reaction at physiological temperatures. In biologically catalyzed reactions, the reactants are known as substrates, and the catalysts are naturally occurring macromolecular substances known as enzymes. Enzymes possess specific binding sites for substrates, and are usually composed wholly or largely of protein, but RNA that has catalytic activity (ribozyme) is often also regarded as enzymatic [goid 3824] [evidence IEA]; Catalysis of the reactions: ATP + 1D-myo-inositol 1,3,4-trisphosphate = ADP + 1D-myo-inositol 1,3,4,5-tetrakisphosphate and ATP + 1D-myo-inositol 1,3,4-trisphosphate = ADP + 1D-myo-inositol 1,3,4,6-tetrakisphosphate [goid 35300] [evidence IEA] |
| Rasl11b | Mus musculus RAS-like, family 11, member B (Rasl11b), mRNA. | 0,37 | 0,001818824 | Interacting selectively with GTP, guanosine triphosphate [goid 5525] [evidence IEA]; Interacting selectively with a nucleotide, any compound consisting of a nucleoside that is esterified with (ortho)phosphate or an oligophosphate at any hydroxyl group on the ribose or deoxyribose moiety [goid 166] [evidence IEA] |
| Rabep2 | Mus musculus rabaptin, RAB GTPase binding effector protein 2 (Rabep2), mRNA. | 0,37 | 0,01187323 | Increases the activity of a GTPase, an enzyme that catalyzes the hydrolysis of GTP [goid 5096] [evidence IEA]; The function that stimulates a cell to grow or proliferate. Most growth factors have other actions besides the induction of cell growth or proliferation [goid 8083] [evidence IEA] |
| Rnpepl1 | Mus musculus arginyl aminopeptidase (aminopeptidase B)-like 1 (Rnpepl1), mRNA. | 0,38 | 0,0000185594 | Catalysis of the hydrolysis of N-terminal amino acid residues from in a polypeptide chain [goid 4177] [evidence IEA] |
| Atxn7l2 | Mus musculus ataxin 7-like 2 (Atxn7l2), mRNA. | 0,38 | 0,002951776 | Elemental activities, such as catalysis or binding, describing the actions of a gene product at the molecular level. A given gene product may exhibit one or more molecular functions [goid 3674] [evidence ND ] |
| Slc1a3 | Mus musculus solute carrier family 1 (glial high affinity glutamate transporter), member 3 (Slc1a3), mRNA. | 0,38 | 0,0000003536137 | Enables the active transport of a solute across a membrane by a mechanism whereby two or more species are transported together in the same direction in a tightly coupled process not directly linked to a form of energy other than chemiosmotic energy [goid 15293] [evidence IEA]; Catalysis of the transfer of a solute or solutes from one side of a membrane to the other according to the reaction: dicarboxylate(out) + Na+(out) = dicarboxylate(in) + Na+(in) [goid 17153] [evidence IEA]; Interacting selectively with an amino acid, organic acids containing one or more amino substituents [goid 16597] [evidence IDA]; Interacting selectively with glutamate, the anion of 2-aminopentanedioic acid [goid 16595] [evidence IDA]; Catalysis of the transfer of a solute or solutes from one side of a membrane to the other according to the reaction: glutamate(out) + H+(out) = glutamate(in) + H+(in) [goid 5314] [evidence IDA]; Interacting selectively with any protein or protein complex (a complex of two or more proteins that may include other nonprotein molecules) [goid 5515] [evidence IPI] |
| Trove2 | Mus musculus TROVE domain family, member 2 (Trove2), mRNA. | 0,39 | 0,0000114425 | Interacting selectively with any protein or protein complex (a complex of two or more proteins that may include other nonprotein molecules) [goid 5515] [evidence ISO]; Interacting selectively with an RNA molecule or a portion thereof [goid 3723] [evidence ISO]; Interacting selectively with any nucleic acid [goid 3676] [evidence IEA] |
| Cdk5rap2 | Mus musculus CDK5 regulatory subunit associated protein 2 (Cdk5rap2), mRNA. | 0,39 | 0,006851834 | Elemental activities, such as catalysis or binding, describing the actions of a gene product at the molecular level. A given gene product may exhibit one or more molecular functions [goid 3674] [evidence ND ] |
| Plekhg5 | Mus musculus pleckstrin homology domain containing, family G (with RhoGef domain) member 5 (Plekhg5), mRNA. | 0,40 | 3,37529E-13 | Stimulates the exchange of guanyl nucleotides by a GTPase of the Rho family. Under normal cellular physiological conditions, the concentration of GTP is higher than that of GDP, favoring the replacement of GDP by GTP in association with the GTPase [goid 5089] [evidence IDA]; Interacting selectively with any protein or protein complex (a complex of two or more proteins that may include other nonprotein molecules) [goid 5515] [evidence IPI] |
| Prkd2 | Mus musculus protein kinase D2 (Prkd2), mRNA. | 0,40 | 0,03602055 | Catalysis of the transfer of a phosphate group, usually from ATP, to a substrate molecule [goid 16301] [evidence IEA]; Interacting selectively with a nucleotide, any compound consisting of a nucleoside that is esterified with (ortho)phosphate or an oligophosphate at any hydroxyl group on the ribose or deoxyribose moiety [goid 166] [evidence IEA]; Catalysis of the transfer of a group, e.g. a methyl group, glycosyl group, acyl group, phosphorus-containing, or other groups, from one compound (generally regarded as the donor) to another compound (generally regarded as the acceptor). Transferase is the systematic name for any enzyme of EC class 2 [goid 16740] [evidence IEA]; Catalysis of the phosphorylation of an amino acid residue in a protein, usually according to the reaction: a protein + ATP = a phosphoprotein + ADP [goid 4672] [evidence IEA]; Interacting selectively with diacylglycerol, a diester of glycerol and two fatty acids [goid 19992] [evidence IEA]; Catalysis of the reaction: ATP + a protein serine/threonine = ADP + protein serine/threonine phosphate [goid 4674] [evidence IEA]; Interacting selectively with zinc (Zn) ions [goid 8270] [evidence IEA]; Interacting selectively with any metal ion [goid 46872] [evidence IEA]; Interacting selectively with ATP, adenosine 5'-triphosphate, a universally important coenzyme and enzyme regulator [goid 5524] [evidence IEA]; Catalysis of the reaction: ATP + a protein = ADP + a phosphoprotein, with a requirement for diacylglycerol [goid 4697] [evidence IEA] |
| Gas6 | Mus musculus growth arrest specific 6 (Gas6), mRNA. | 0,41 | 0,00000001437847 | Interacting selectively with any metal ion [goid 46872] [evidence IEA]; Interacting selectively with calcium ions (Ca2+) [goid 5509] [evidence IEA] |
| Cnnm2 | Mus musculus cyclin M2 (Cnnm2), mRNA. | 0,41 | 4,980398E-12 | Elemental activities, such as catalysis or binding, describing the actions of a gene product at the molecular level. A given gene product may exhibit one or more molecular functions [goid 3674] [evidence ND ] |
| Acads | Mus musculus acyl-Coenzyme A dehydrogenase, short chain (Acads), nuclear gene encoding mitochondrial protein, mRNA. | 0,42 | 0,0439488 | Catalysis of an oxidation-reduction (redox) reaction, a reversible chemical reaction in which the oxidation state of an atom or atoms within a molecule is altered. One substrate acts as a hydrogen or electron donor and becomes oxidized, while the other acts as hydrogen or electron acceptor and becomes reduced [goid 16491] [evidence IEA]; Interacting selectively with FAD, flavin-adenine dinucleotide, the coenzyme or the prosthetic group of various flavoprotein oxidoreductase enzymes [goid 50660] [evidence IEA]; Catalysis of an oxidation-reduction (redox) reaction in which a CH-CH group acts as a hydrogen or electron donor and reduces a hydrogen or electron acceptor [goid 16627] [evidence IEA]; Catalysis of the reaction: acyl-CoA + acceptor = 2,3-dehydroacyl-CoA + reduced acceptor [goid 3995] [evidence IEA]; Any molecular entity that serves as an electron acceptor and electron donor in an electron transport system [goid 9055] [evidence IEA]; Catalysis of the reaction: butanoyl-CoA + ETF = 2-butenoyl-CoA + reduced ETF [goid 4085] [evidence IEA] |
| Rasl11a | Mus musculus RAS-like, family 11, member A (Rasl11a), mRNA. | 0,42 | 0,01910719 | Interacting selectively with GTP, guanosine triphosphate [goid 5525] [evidence IEA]; Interacting selectively with a nucleotide, any compound consisting of a nucleoside that is esterified with (ortho)phosphate or an oligophosphate at any hydroxyl group on the ribose or deoxyribose moiety [goid 166] [evidence IEA] |
| Cc2d1a | Mus musculus coiled-coil and C2 domain containing 1A (Cc2d1a), mRNA. | 0,42 | 0,0000003104253 | Interacting selectively with DNA (deoxyribonucleic acid) [goid 3677] [evidence IEA] |
| Mfsd10 | Mus musculus major facilitator superfamily domain containing 10 (Mfsd10), mRNA. | 0,42 | 6,011855E-16 | Enables the directed movement of substances (such as macromolecules, small molecules, ions) into, out of, within or between cells [goid 5215] [evidence IEA] |
| Dab2ip | Mus musculus disabled homolog 2 (Drosophila) interacting protein (Dab2ip), mRNA. | 0,42 | 0,006334028 | Increases the activity of a GTPase, an enzyme that catalyzes the hydrolysis of GTP [goid 5096] [evidence IEA]; Interacting selectively with any protein or protein complex (a complex of two or more proteins that may include other nonprotein molecules) [goid 5515] [evidence IPI] |
| Tcf3 | Mus musculus transcription factor 3 (Tcf3), transcript variant 1, mRNA. | 0,42 | 0,01871855 | Interacting selectively with the beta subunit of the catenin complex [goid 8013] [evidence IPI]; Interacting selectively with DNA (deoxyribonucleic acid) [goid 3677] [evidence IDA]; The function of binding to a specific DNA sequence in order to modulate transcription. The transcription factor may or may not also interact selectively with a protein or macromolecular complex [goid 3700] [evidence IDA] |
| Slc19a1 | Mus musculus solute carrier family 19 (sodium/hydrogen exchanger), member 1 (Slc19a1), mRNA. | 0,44 | 0,02255579 | Interacting selectively with folic acid, pteroylglutamic acid. Folic acid is widely distributed as a member of the vitamin B complex and is essential for the synthesis of purine and pyrimidines [goid 5542] [evidence IEA]; Catalysis of the transfer of reduced folate from one side of a membrane to the other, up its concentration gradient. The transporter binds the solute and undergoes a series of conformational changes. Transport works equally well in either direction and is driven by a chemiosmotic source of energy. Chemiosmotic sources of energy include uniport, symport or antiport [goid 8518] [evidence IEA] |
| Stat3 | Mus musculus signal transducer and activator of transcription 3 (Stat3), transcript variant 1, mRNA. | 0,44 | 0,00000003785994 | The function of binding to a specific DNA sequence in order to modulate transcription. The transcription factor may or may not also interact selectively with a protein or macromolecular complex [goid 3700] [evidence IEA]; Interacting selectively with calcium ions (Ca2+) [goid 5509] [evidence IEA]; Mediates the transfer of a signal from the outside to the inside of a cell by means other than the introduction of the signal molecule itself into the cell [goid 4871] [evidence IEA]; Interacting selectively with any protein or protein complex (a complex of two or more proteins that may include other nonprotein molecules) [goid 5515] [evidence IPI]; Interacting selectively with DNA (deoxyribonucleic acid) [goid 3677] [evidence IDA]; Any transcription regulator activity required for initiation or upregulation of transcription [goid 16563] [evidence IDA]; The formation of a protein dimer, a macromolecular structure consists of two noncovalently associated identical or nonidentical subunits [goid 46983] [evidence IPI]; Interacting selectively with DNA of a specific nucleotide composition, e.g. GC-rich DNA binding, or with a specific sequence motif or type of DNA e.g. promotor binding or rDNA binding [goid 43565] [evidence IDA] |
| Ahi1 | Mus musculus Abelson helper integration site (Ahi1), mRNA. | 0,45 | 0,03287045 | Elemental activities, such as catalysis or binding, describing the actions of a gene product at the molecular level. A given gene product may exhibit one or more molecular functions [goid 3674] [evidence ND ] |
| Bat3 | Mus musculus HLA-B-associated transcript 3 (Bat3), mRNA. | 0,45 | 0,000002802382 | Interacting selectively with any protein or protein complex (a complex of two or more proteins that may include other nonprotein molecules) [goid 5515] [evidence IPI] |
| Col6a1 |  | 0,48 | 0,0147667 | Interacting selectively with any protein or protein complex (a complex of two or more proteins that may include other nonprotein molecules) [goid 5515] [evidence IEA]; The action of a molecule that contributes to the structural integrity of a complex or assembly within or outside a cell [goid 5198] [evidence IEA]; Interacting selectively with platelet-derived growth factor [goid 48407] [evidence ISO] |
| Bag3 | Mus musculus BCL2-associated athanogene 3 (Bag3), mRNA. | 0,49 | 9,147752E-12 | Interacting selectively with any protein or protein complex (a complex of two or more proteins that may include other nonprotein molecules) [goid 5515] [evidence IPI] |
| Morc2a | Mus musculus microrchidia 2A (Morc2a), mRNA. | 0,49 | 0,000000001861762 | Interacting selectively with any metal ion [goid 46872] [evidence IEA]; Interacting selectively with zinc (Zn) ions [goid 8270] [evidence IEA]; Interacting selectively with ATP, adenosine 5'-triphosphate, a universally important coenzyme and enzyme regulator [goid 5524] [evidence IEA] |
| Mib2 | Mus musculus mindbomb homolog 2 (Drosophila) (Mib2), mRNA. | 0,49 | 0,00005306462 | Catalysis of the reaction: ATP + ubiquitin + protein lysine = AMP + diphosphate + protein N-ubiquityllysine [goid 4842] [evidence IDA]; Interacting selectively with monomeric or multimeric forms of actin, including actin filaments [goid 3779] [evidence IEA]; Interacting selectively with zinc (Zn) ions [goid 8270] [evidence IEA]; Interacting selectively with any metal ion [goid 46872] [evidence IEA]; Catalysis of the ligation of two substances with concomitant breaking of a diphosphate linkage, usually in a nucleoside triphosphate. Ligase is the systematic name for any enzyme of EC class 6 [goid 16874] [evidence IEA]; Interacting selectively with any protein or protein complex (a complex of two or more proteins that may include other nonprotein molecules) [goid 5515] [evidence IEA] |
| Lamb2 | Mus musculus laminin, beta 2 (Lamb2), mRNA. | 0,49 | 5,846789E-10 | Interacting selectively with any protein or protein complex (a complex of two or more proteins that may include other nonprotein molecules) [goid 5515] [evidence IEA] |
| Vars | Mus musculus valyl-tRNA synthetase (Vars), nuclear gene encoding mitochondrial protein, mRNA. | 0,50 | 0,02647614 | Catalysis of the formation of aminoacyl-tRNA from ATP, amino acid, and tRNA with the release of pyrophosphate and AMP [goid 4812] [evidence IEA]; Catalysis of the ligation of two substances with concomitant breaking of a diphosphate linkage, usually in a nucleoside triphosphate. Ligase is the systematic name for any enzyme of EC class 6 [goid 16874] [evidence IEA]; Interacting selectively with ATP, adenosine 5'-triphosphate, a universally important coenzyme and enzyme regulator [goid 5524] [evidence IEA]; Interacting selectively with a nucleotide, any compound consisting of a nucleoside that is esterified with (ortho)phosphate or an oligophosphate at any hydroxyl group on the ribose or deoxyribose moiety [goid 166] [evidence IEA]; Catalysis of the reaction: ATP + L-valine + tRNA(Val) = AMP + diphosphate + L-valyl-tRNA(Val) [goid 4832] [evidence IEA] |
| Tubg1 | Mus musculus tubulin, gamma 1 (Tubg1), mRNA. | 0,50 | 0,0000006050007 | Interacting selectively with a nucleotide, any compound consisting of a nucleoside that is esterified with (ortho)phosphate or an oligophosphate at any hydroxyl group on the ribose or deoxyribose moiety [goid 166] [evidence IEA]; Interacting selectively with GTP, guanosine triphosphate [goid 5525] [evidence IEA]; Catalysis of the reaction: GTP + H2O = GDP + phosphate [goid 3924] [evidence IEA]; The action of a molecule that contributes to the structural integrity of a cytoskeletal structure [goid 5200] [evidence TAS] |
| Akr7a5 | Mus musculus aldo-keto reductase family 7, member A5 (aflatoxin aldehyde reductase) (Akr7a5), mRNA. | 0,51 | 0,001017411 | Catalysis of an oxidation-reduction (redox) reaction, a reversible chemical reaction in which the oxidation state of an atom or atoms within a molecule is altered. One substrate acts as a hydrogen or electron donor and becomes oxidized, while the other acts as hydrogen or electron acceptor and becomes reduced [goid 16491] [evidence IEA] |
| Zyx | Mus musculus zyxin (Zyx), mRNA. | 0,51 | 0,000004127798 | Interacting selectively with any protein or protein complex (a complex of two or more proteins that may include other nonprotein molecules) [goid 5515] [evidence IPI]; Interacting selectively with zinc (Zn) ions [goid 8270] [evidence IEA]; Interacting selectively with any metal ion [goid 46872] [evidence IEA] |
| Ncoa5 | Mus musculus nuclear receptor coactivator 5 (Ncoa5), mRNA. | 0,51 | 0,00000008966942 | Combining with an extracellular or intracellular messenger to initiate a change in cell activity [goid 4872] [evidence IEA] |
| Zmym3 | Mus musculus zinc finger, MYM-type 3 (Zmym3), mRNA. | 0,52 | 0,00001670147 | Interacting selectively with any metal ion [goid 46872] [evidence IEA]; Interacting selectively with zinc (Zn) ions [goid 8270] [evidence IEA] |
| Ints4 | Mus musculus integrator complex subunit 4 (Ints4), mRNA. | 0,53 | 0,00001524028 | The selective, often stoichiometric, interaction of a molecule with one or more specific sites on another molecule [goid 5488] [evidence IEA] |
| Pitpna | Mus musculus phosphatidylinositol transfer protein, alpha (Pitpna), mRNA. | 0,53 | 0,01068643 | Interacting selectively with a lipid [goid 8289] [evidence ISS]; Interacting selectively with phospholipids, a class of lipids containing phosphoric acid as a mono- or diester [goid 5543] [evidence ISO] |
| Ddx27 | Mus musculus DEAD (Asp-Glu-Ala-Asp) box polypeptide 27 (Ddx27), mRNA. | 0,54 | 0,005607701 | Catalysis of the hydrolysis of various bonds, e.g. C-O, C-N, C-C, phosphoric anhydride bonds, etc. Hydrolase is the systematic name for any enzyme of EC class 3 [goid 16787] [evidence IEA]; Catalysis of the reaction: NTP + H2O = NDP + phosphate to drive the unwinding of a DNA or RNA helix [goid 4386] [evidence IEA]; Interacting selectively with a nucleotide, any compound consisting of a nucleoside that is esterified with (ortho)phosphate or an oligophosphate at any hydroxyl group on the ribose or deoxyribose moiety [goid 166] [evidence IEA]; Interacting selectively with any nucleic acid [goid 3676] [evidence IEA]; Catalysis of the reaction: ATP + H2O = ADP + phosphate to drive the unwinding of a DNA or RNA helix [goid 8026] [evidence IEA]; Interacting selectively with ATP, adenosine 5'-triphosphate, a universally important coenzyme and enzyme regulator [goid 5524] [evidence IEA] |
| Slc25a45 | Mus musculus solute carrier family 25, member 45 (Slc25a45), mRNA. | 0,54 | 0,00004145977 | Enables the directed movement of substances (such as macromolecules, small molecules, ions) into, out of, within or between cells [goid 5215] [evidence IEA]; The selective, often stoichiometric, interaction of a molecule with one or more specific sites on another molecule [goid 5488] [evidence IEA] |
| 6330569M22Rik | Mus musculus RIKEN cDNA 6330569M22 gene (6330569M22Rik), mRNA. | 0,55 | 0,000000861473 | Elemental activities, such as catalysis or binding, describing the actions of a gene product at the molecular level. A given gene product may exhibit one or more molecular functions [goid 3674] [evidence ND ] |
| Pnpla2 | Mus musculus patatin-like phospholipase domain containing 2 (Pnpla2), mRNA. | 0,55 | 0,002761953 | Catalysis of the hydrolysis of various bonds, e.g. C-O, C-N, C-C, phosphoric anhydride bonds, etc. Hydrolase is the systematic name for any enzyme of EC class 3 [goid 16787] [evidence IEA]; Catalysis of the reaction: triacylglycerol + H2O = diacylglycerol + a fatty acid anion [goid 4806] [evidence IMP] |
| Prpf19 | Mus musculus PRP19/PSO4 pre-mRNA processing factor 19 homolog (S. cerevisiae) (Prpf19), mRNA. | 0,55 | 0,000004430484 | Catalysis of the reaction: Catalysis of the reaction: ATP + ubiquitin + protein N-ubiquityl(n)lysine = AMP + diphosphate + protein N-ubiquityl(n+1)lysine, i.e. the ligation of ubiquitin moieties, via isopeptide bonds, to extend the length of the multiubiquitin chain attached to a substrate protein [goid 34450] [evidence ISO]; Interacting selectively with DNA (deoxyribonucleic acid) [goid 3677] [evidence IEA]; Catalysis of the reaction: ATP + ubiquitin + protein lysine = AMP + diphosphate + protein N-ubiquityllysine [goid 4842] [evidence ISO]; Interacting selectively with any protein or protein complex (a complex of two or more proteins that may include other nonprotein molecules) [goid 5515] [evidence IPI] |
| 2610024G14Rik |  | 0,56 | 0,01374538 | Elemental activities, such as catalysis or binding, describing the actions of a gene product at the molecular level. A given gene product may exhibit one or more molecular functions [goid 3674] [evidence ND ]; Interacting selectively with GTP, guanosine triphosphate [goid 5525] [evidence IEA] |
| Dnmt3l | Mus musculus DNA (cytosine-5-)-methyltransferase 3-like (Dnmt3l), transcript variant 2, mRNA. | 0,57 | 0,007127667 | Any transcription regulator activity that prevents or downregulates transcription [goid 16564] [evidence ISS]; Catalysis of the transfer of a group, e.g. a methyl group, glycosyl group, acyl group, phosphorus-containing, or other groups, from one compound (generally regarded as the donor) to another compound (generally regarded as the acceptor). Transferase is the systematic name for any enzyme of EC class 2 [goid 16740] [evidence IEA]; Catalysis of the transfer of a methyl group to an acceptor molecule [goid 8168] [evidence IEA]; Interacting selectively with any metal ion [goid 46872] [evidence IEA]; Interacting selectively with zinc (Zn) ions [goid 8270] [evidence IEA]; Increases the activity of an enzyme [goid 8047] [evidence IDA]; Increases the activity of an enzyme [goid 8047] [evidence ISO]; Interacting selectively with any enzyme [goid 19899] [evidence ISS]; Interacting selectively with any protein or protein complex (a complex of two or more proteins that may include other nonprotein molecules) [goid 5515] [evidence IPI] |
| 3110056O03Rik | Mus musculus RIKEN cDNA 3110056O03 gene (3110056O03Rik), mRNA. | 0,57 | 0,00003841332 | Catalysis of the hydrolysis of a peptide bond. A peptide bond is a covalent bond formed when the carbon atom from the carboxyl group of one amino acid shares electrons with the nitrogen atom from the amino group of a second amino acid [goid 8233] [evidence IEA]; Catalysis of the hydrolysis of various bonds, e.g. C-O, C-N, C-C, phosphoric anhydride bonds, etc. Hydrolase is the systematic name for any enzyme of EC class 3 [goid 16787] [evidence IEA]; Catalysis of the hydrolysis of internal, alpha-peptide bonds in a polypeptide chain by a mechanism in which a water molecule bound by the side chains of aspartic residues at the active center acts as a nucleophile [goid 4190] [evidence IEA] |
| Rhbdf1 | Mus musculus rhomboid family 1 (Drosophila) (Rhbdf1), mRNA. | 0,58 | 0,00000001228887 | Combining with an extracellular or intracellular messenger to initiate a change in cell activity [goid 4872] [evidence IEA] |
| Grit | Mus musculus Rho GTPase-activating protein (Grit), mRNA. | 0,58 | 0,004810738 | Increases the activity of a GTPase, an enzyme that catalyzes the hydrolysis of GTP [goid 5096] [evidence ISO] |
| Usp22 | Mus musculus ubiquitin specific peptidase 22 (Usp22), mRNA. | 0,59 | 0,006094475 | Interacting selectively with any metal ion [goid 46872] [evidence IEA]; Catalysis of the hydrolysis of various bonds, e.g. C-O, C-N, C-C, phosphoric anhydride bonds, etc. Hydrolase is the systematic name for any enzyme of EC class 3 [goid 16787] [evidence IEA]; Interacting selectively with zinc (Zn) ions [goid 8270] [evidence IEA]; Catalysis of the hydrolysis of a peptide bond. A peptide bond is a covalent bond formed when the carbon atom from the carboxyl group of one amino acid shares electrons with the nitrogen atom from the amino group of a second amino acid [goid 8233] [evidence IEA]; Catalysis of the hydrolysis of peptide bonds in a polypeptide chain by a mechanism in which the sulfhydryl group of a cysteine residue at the active center acts as a nucleophile [goid 8234] [evidence IEA]; Catalysis of the reaction: ubiquitin C-terminal thiolester + H2O = ubiquitin + a thiol. Hydrolysis of esters, including those formed between thiols such as dithiothreitol or glutathione and the C-terminal glycine residue of the polypeptide ubiquitin, and AMP-ubiquitin [goid 4221] [evidence IEA]; Catalysis of the hydrolysis of various forms of polymeric ubiquitin sequences. Will remove ubiquitin from larger leaving groups [goid 4843] [evidence IDA] |
| Ptdss2 | Mus musculus phosphatidylserine synthase 2 (Ptdss2), mRNA. | 0,60 | 0,008428572 | Catalysis of the transfer of a group, e.g. a methyl group, glycosyl group, acyl group, phosphorus-containing, or other groups, from one compound (generally regarded as the donor) to another compound (generally regarded as the acceptor). Transferase is the systematic name for any enzyme of EC class 2 [goid 16740] [evidence IEA]; Catalysis of the reaction: CDP-diacylglycerol + L-serine = CMP + O-sn-phosphatidyl-L-serine [goid 3882] [evidence IMP] |
| Adck5 | Mus musculus aarF domain containing kinase 5 (Adck5), mRNA. | 0,60 | 0,000988367 | Catalysis of the reaction: ATP + a protein serine/threonine = ADP + protein serine/threonine phosphate [goid 4674] [evidence IEA]; Catalysis of the transfer of a group, e.g. a methyl group, glycosyl group, acyl group, phosphorus-containing, or other groups, from one compound (generally regarded as the donor) to another compound (generally regarded as the acceptor). Transferase is the systematic name for any enzyme of EC class 2 [goid 16740] [evidence IEA]; Catalysis of the transfer of a phosphate group, usually from ATP, to a substrate molecule [goid 16301] [evidence IEA]; Interacting selectively with ATP, adenosine 5'-triphosphate, a universally important coenzyme and enzyme regulator [goid 5524] [evidence IEA]; Catalysis of the phosphorylation of an amino acid residue in a protein, usually according to the reaction: a protein + ATP = a phosphoprotein + ADP [goid 4672] [evidence IEA] |
| Stk11 | Mus musculus serine/threonine kinase 11 (Stk11), mRNA. | 0,60 | 0,03389045 | Interacting selectively with manganese (Mn) ions [goid 30145] [evidence IEA]; Interacting selectively with a nucleotide, any compound consisting of a nucleoside that is esterified with (ortho)phosphate or an oligophosphate at any hydroxyl group on the ribose or deoxyribose moiety [goid 166] [evidence IEA]; Catalysis of the reaction: ATP + a protein serine/threonine = ADP + protein serine/threonine phosphate [goid 4674] [evidence IEA]; Interacting selectively with any metal ion [goid 46872] [evidence IEA]; Interacting selectively with ATP, adenosine 5'-triphosphate, a universally important coenzyme and enzyme regulator [goid 5524] [evidence IEA]; Catalysis of the phosphorylation of an amino acid residue in a protein, usually according to the reaction: a protein + ATP = a phosphoprotein + ADP [goid 4672] [evidence IEA]; Catalysis of the transfer of a phosphate group, usually from ATP, to a substrate molecule [goid 16301] [evidence IEA]; Interacting selectively with magnesium (Mg) ions [goid 287] [evidence IEA]; Catalysis of the transfer of a group, e.g. a methyl group, glycosyl group, acyl group, phosphorus-containing, or other groups, from one compound (generally regarded as the donor) to another compound (generally regarded as the acceptor). Transferase is the systematic name for any enzyme of EC class 2 [goid 16740] [evidence IEA] |
| Axl | Mus musculus AXL receptor tyrosine kinase (Axl), mRNA. | 0,62 | 0,000001485075 | Interacting selectively with ATP, adenosine 5'-triphosphate, a universally important coenzyme and enzyme regulator [goid 5524] [evidence IEA]; Catalysis of the reaction: ATP + a protein tyrosine = ADP + protein tyrosine phosphate [goid 4713] [evidence IEA]; Catalysis of the reaction: ATP + a protein-L-tyrosine = ADP + a protein-L-tyrosine phosphate, to initiate a change in cell activity [goid 4714] [evidence IEA]; Catalysis of the transfer of a phosphate group, usually from ATP, to a substrate molecule [goid 16301] [evidence IEA]; Interacting selectively with a nucleotide, any compound consisting of a nucleoside that is esterified with (ortho)phosphate or an oligophosphate at any hydroxyl group on the ribose or deoxyribose moiety [goid 166] [evidence IEA]; Catalysis of the transfer of a group, e.g. a methyl group, glycosyl group, acyl group, phosphorus-containing, or other groups, from one compound (generally regarded as the donor) to another compound (generally regarded as the acceptor). Transferase is the systematic name for any enzyme of EC class 2 [goid 16740] [evidence IEA]; Combining with an extracellular or intracellular messenger to initiate a change in cell activity [goid 4872] [evidence IEA]; Catalysis of the phosphorylation of an amino acid residue in a protein, usually according to the reaction: a protein + ATP = a phosphoprotein + ADP [goid 4672] [evidence IEA]; Interacting selectively with any protein or protein complex (a complex of two or more proteins that may include other nonprotein molecules) [goid 5515] [evidence IPI] |
| Map3k3 | Mus musculus mitogen-activated protein kinase kinase kinase 3 (Map3k3), mRNA. | 0,62 | 0,03017709 | Catalysis of the transfer of a phosphate group, usually from ATP, to a substrate molecule [goid 16301] [evidence IEA]; Interacting selectively with magnesium (Mg) ions [goid 287] [evidence IEA]; Interacting selectively with a nucleotide, any compound consisting of a nucleoside that is esterified with (ortho)phosphate or an oligophosphate at any hydroxyl group on the ribose or deoxyribose moiety [goid 166] [evidence IEA]; Catalysis of the reaction: ATP + a protein serine/threonine = ADP + protein serine/threonine phosphate [goid 4674] [evidence IEA]; Catalysis of the transfer of a group, e.g. a methyl group, glycosyl group, acyl group, phosphorus-containing, or other groups, from one compound (generally regarded as the donor) to another compound (generally regarded as the acceptor). Transferase is the systematic name for any enzyme of EC class 2 [goid 16740] [evidence IEA]; Interacting selectively with ATP, adenosine 5'-triphosphate, a universally important coenzyme and enzyme regulator [goid 5524] [evidence IEA]; Interacting selectively with any protein or protein complex (a complex of two or more proteins that may include other nonprotein molecules) [goid 5515] [evidence IPI]; Catalysis of the phosphorylation and activation of MAP kinase kinases; each MAP kinase kinase can be phosphorylated by any of several MAP kinase kinase kinases [goid 4709] [evidence IEA]; Catalysis of the phosphorylation of an amino acid residue in a protein, usually according to the reaction: a protein + ATP = a phosphoprotein + ADP [goid 4672] [evidence ISO]; Interacting selectively with any metal ion [goid 46872] [evidence IEA] |
| Dhx38 | Mus musculus DEAH (Asp-Glu-Ala-His) box polypeptide 38 (Dhx38), mRNA. | 0,62 | 0,001857969 | Catalysis of the hydrolysis of various bonds, e.g. C-O, C-N, C-C, phosphoric anhydride bonds, etc. Hydrolase is the systematic name for any enzyme of EC class 3 [goid 16787] [evidence IEA]; Interacting selectively with a nucleotide, any compound consisting of a nucleoside that is esterified with (ortho)phosphate or an oligophosphate at any hydroxyl group on the ribose or deoxyribose moiety [goid 166] [evidence IEA]; Interacting selectively with ATP, adenosine 5'-triphosphate, a universally important coenzyme and enzyme regulator [goid 5524] [evidence IEA]; Catalysis of the reaction: NTP + H2O = NDP + phosphate to drive the unwinding of a DNA or RNA helix [goid 4386] [evidence IEA] |
| Pcyox1 |  | 0,62 | 0,00001617027 | Catalysis of an oxidation-reduction (redox) reaction, a reversible chemical reaction in which the oxidation state of an atom or atoms within a molecule is altered. One substrate acts as a hydrogen or electron donor and becomes oxidized, while the other acts as hydrogen or electron acceptor and becomes reduced [goid 16491] [evidence IEA]; Catalysis of an oxidation-reduction (redox) reaction in which a sulfur-containing group acts as a hydrogen or electron donor and reduces oxygen [goid 16670] [evidence IEA]; Any molecular entity that serves as an electron acceptor and electron donor in an electron transport system [goid 9055] [evidence IEA]; Catalysis of the reaction: S-prenyl-L-cysteine + O2 + H20 = a prenal + L-cysteine + H202 [goid 1735] [evidence IMP] |
| Top3b | Mus musculus topoisomerase (DNA) III beta (Top3b), mRNA. | 0,62 | 0,04455505 | Catalysis of the transient cleavage and passage of individual DNA strands or double helices through one another, resulting a topological transformation in double-stranded DNA [goid 3916] [evidence IEA]; Catalysis of the geometric or structural changes within one molecule. Isomerase is the systematic name for any enzyme of EC class 5 [goid 16853] [evidence IEA]; Interacting selectively with DNA (deoxyribonucleic acid) [goid 3677] [evidence IEA]; Catalysis of a DNA topological transformation by transiently cleaving one DNA strand at a time to allow passage of another strand; changes the linking number by +1 per catalytic cycle [goid 3917] [evidence IEA]; Interacting selectively with any nucleic acid [goid 3676] [evidence IEA] |
| Appl2 | Mus musculus adaptor protein, phosphotyrosine interaction, PH domain and leucine zipper containing 2 (Appl2), mRNA. | 0,63 | 0,01017436 | Interacting selectively with Rab protein, any member of the Rab subfamily of the Ras superfamily of monomeric GTPases [goid 17137] [evidence ISO] |
| Mrps34 | Mus musculus mitochondrial ribosomal protein S34 (Mrps34), nuclear gene encoding mitochondrial protein, mRNA. | 0,65 | 0,01009873 | Elemental activities, such as catalysis or binding, describing the actions of a gene product at the molecular level. A given gene product may exhibit one or more molecular functions [goid 3674] [evidence ND ] |
| Gtpbp2 | Mus musculus GTP binding protein 2 (Gtpbp2), mRNA. | 0,66 | 0,006721411 | Interacting selectively with a nucleotide, any compound consisting of a nucleoside that is esterified with (ortho)phosphate or an oligophosphate at any hydroxyl group on the ribose or deoxyribose moiety [goid 166] [evidence IEA]; Catalysis of the reaction: GTP + H2O = GDP + phosphate [goid 3924] [evidence IEA]; Interacting selectively with GTP, guanosine triphosphate [goid 5525] [evidence ISS] |
| Atp1b1 | Mus musculus ATPase, Na+/K+ transporting, beta 1 polypeptide (Atp1b1), mRNA. | 0,66 | 0,0007140686 | Interacting selectively with any protein or protein complex (a complex of two or more proteins that may include other nonprotein molecules) [goid 5515] [evidence IPI]; Interacting selectively with potassium (K+) ions [goid 30955] [evidence IEA]; Interacting selectively with sodium ions (Na+) [goid 31402] [evidence IEA]; Catalysis of the transfer of a solute or solutes from one side of a membrane to the other according to the reaction: ATP + H2O + Na+(in) + K+(out) = ADP + phosphate + Na+(out) + K+(in) [goid 5391] [evidence IEA] |
| Gak | Mus musculus cyclin G associated kinase (Gak), mRNA. | 0,67 | 0,0007115101 | Catalysis of the transfer of a phosphate group, usually from ATP, to a substrate molecule [goid 16301] [evidence IEA]; Interacting selectively with a nucleotide, any compound consisting of a nucleoside that is esterified with (ortho)phosphate or an oligophosphate at any hydroxyl group on the ribose or deoxyribose moiety [goid 166] [evidence IEA]; Catalysis of the reaction: ATP + a protein serine/threonine = ADP + protein serine/threonine phosphate [goid 4674] [evidence IEA]; Catalysis of the transfer of a group, e.g. a methyl group, glycosyl group, acyl group, phosphorus-containing, or other groups, from one compound (generally regarded as the donor) to another compound (generally regarded as the acceptor). Transferase is the systematic name for any enzyme of EC class 2 [goid 16740] [evidence IEA]; Interacting selectively with ATP, adenosine 5'-triphosphate, a universally important coenzyme and enzyme regulator [goid 5524] [evidence IEA]; Interacting selectvely with a heat shock protein, any protein synthesized or activated in response to heat shock [goid 31072] [evidence IEA]; Catalysis of the phosphorylation of an amino acid residue in a protein, usually according to the reaction: a protein + ATP = a phosphoprotein + ADP [goid 4672] [evidence IEA]; Interacting selectively with any protein or protein complex (a complex of two or more proteins that may include other nonprotein molecules) [goid 5515] [evidence IPI] |
